# Supplementary material for: Axial length to corneal radius of curvature ratio and refractive error in Chinese preschoolers aged 4–6 years: a retrospective cross-sectional study
Source: BMJ Open. 2023 Dec 30;13(12):e075115. doi: 10.1136/bmjopen-2023-075115 (PMC10759075; doi:10.1136/bmjopen-2023-075115)
Supplement: Supplementary data [file bmjopen-2023-075115supp006.pdf]

Supplemental Table 4. Summary of studies reporting the AL, CRC, and AL/CRC ratio.

Origin	N	Age (years)	SER (D)	AL/CRC	AL	CRC
Australia [15]	1765	5.5-8.4	+1.26	2.906	22.61	7.70
Iran [17]	4820	40-64	-	3.033	23.14	7.63
China [19]	4411	7-10	-0.13	2.97	23.27	7.82
Singapore [28]	349	3	+0.88	2.81	21.73	7.72
Nigerian [34]	70	20-39	-0.24	3.03	23.74	7.84
Myanmar [35]	1498	40-70	-1.22	2.99	22.76	7.62
Japan [43]	457	4-6	+0.13	2.88	22.35	7.76
Current study	1024	4-6	+0.90	2.92	22.59	7.74

SER, spherical equivalent refractive error; AL, axial length; CRC, corneal radius of curvature; AL/CRC ratio, axial length to corneal radius of curvature ratio.
