# Supplementary material for: Axial length to corneal radius of curvature ratio and refractive error in Chinese preschoolers aged 4–6 years: a retrospective cross-sectional study
Source: BMJ Open. 2023 Dec 30;13(12):e075115. doi: 10.1136/bmjopen-2023-075115 (PMC10759075; doi:10.1136/bmjopen-2023-075115)
Supplement: Supplementary data [file bmjopen-2023-075115supp005.pdf]

Supplemental Table 3. Effect of optimal cut-off value of AL/CRC ratio for the lack of hyperopia reserve assessment and true myopia assessment.

	Criterion	Total Data	True assessment	Error assessment (%)
Lack of hyperopia reserve assessment	AL/CRC ratio ≥2.955	252	196	56 (22.22%)
True myopia assessment	AL/CRC ratio ≥2.975	85	79	6 (7.06%)

AL/CRC ratio, axial length to corneal radius of curvature ratio. Error assessment (%), using optimal cut-off value of AL/CRC ratio to assess the probability of errors in lack of hyperopia reserve or true myopia.
