# Supplementary material for: Axial length to corneal radius of curvature ratio and refractive error in Chinese preschoolers aged 4–6 years: a retrospective cross-sectional study
Source: BMJ Open. 2023 Dec 30;13(12):e075115. doi: 10.1136/bmjopen-2023-075115 (PMC10759075; doi:10.1136/bmjopen-2023-075115)
Supplement: Supplementary data [file bmjopen-2023-075115supp004.pdf]

Supplemental Table 2. Association between AL/CRC ratio, AL, and CRC with SER.

Variables	Correlation coefficient	P value	Univariate OR (95% CI)	P value	Gender and age-adjusted Multivariate OR (95% CI)				
					Unstandardised coefficient	95% CI	Standardised coefficient	P value	R^2
AL	-0.52	<0.001	-0.86 (-0.94 to -0.79)	<0.001	-0.869	-0.942 to -0.795	-0.590	<0.001	0.345
CRC	-0.03	0.33	0.15 (-0.14 to 0.43)	0.31	0.148	-0.135 to 0.430	0.032	0.306	0.002
AL/CRC	-0.66	<0.001	-11.73 (-12.27 to -11.19)	<0.001	-11.876	-12.418 to -11.334	-0.809	<0.001	0.645

SER, spherical equivalent refractive error; AL, axial length; CRC, corneal radius of curvature; AL/CRC ratio, axial length to corneal radius of curvature ratio. OR, odds ratio.
