# Supplementary material for: Axial length to corneal radius of curvature ratio and refractive error in Chinese preschoolers aged 4–6 years: a retrospective cross-sectional study
Source: BMJ Open. 2023 Dec 30;13(12):e075115. doi: 10.1136/bmjopen-2023-075115 (PMC10759075; doi:10.1136/bmjopen-2023-075115)
Supplement: Supplementary data [file bmjopen-2023-075115supp003.pdf]

Supplemental Table 1. Demographic and biometric data of included participants

Parameters	Total, N=1024		Boys, N=537	Girls, N=487	P value
	Mean±SD	Range	Mean±SD	Mean±SD	
Age (years)	5.77±0.47	4-6	5.85±0.35	5.68±0.56	<0.001
SER (D)	0.90±1.11	-6.38 to 8.50	0.90±1.09	0.90±1.13	0.922
AL (mm)	22.59±0.76	20.24 to 25.28	22.62±0.77	22.57±0.74	0.350
CRC (mm)	7.74±0.24	6.98 to 8.54	7.74±0.24	7.74±0.24	0.805
AL/CRC	2.92±0.08	2.65 to 3.17	2.92±0.08	2.92±0.08	0.141
LP (D)	24.09±1.35	19.68 to 28.68	24.02±1.34	24.16±1.35	0.117
ACD (mm)	3.37±0.27	2.09 to 5.37	3.38±0.27	3.34±0.27	0.028

SER, spherical equivalent refractive error; AL, axial length; CRC, corneal radius of curvature; AL/CRC, axial length to corneal radius of curvature ratio; LP, lens power; ACD, anterior chamber depth; D, diopter.
