# Supplementary material for: Axial length to corneal radius of curvature ratio and refractive error in Chinese preschoolers aged 4–6 years: a retrospective cross-sectional study
Source: BMJ Open. 2023 Dec 30;13(12):e075115. doi: 10.1136/bmjopen-2023-075115 (PMC10759075; doi:10.1136/bmjopen-2023-075115)
Supplement: Supplementary data [file bmjopen-2023-075115supp002.pdf]

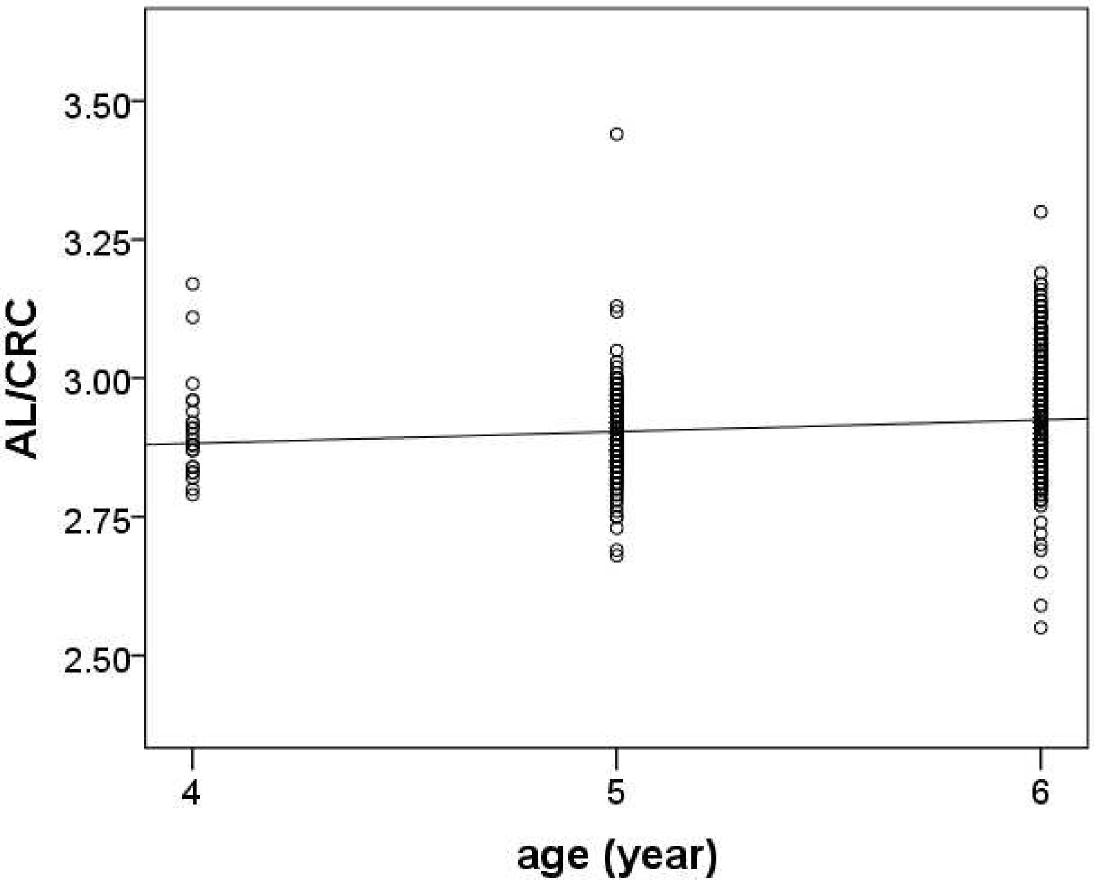

Supplemental Figure 2. Scatterplot of AL/CRC ratio and age. AL/CRC, axial length to corneal radius of curvature ratio.
