# Supplementary material for: Axial length to corneal radius of curvature ratio and refractive error in Chinese preschoolers aged 4–6 years: a retrospective cross-sectional study
Source: BMJ Open. 2023 Dec 30;13(12):e075115. doi: 10.1136/bmjopen-2023-075115 (PMC10759075; doi:10.1136/bmjopen-2023-075115)
Supplement: Author's manuscript [file bmjopen-2023-075115.draft_revisions.pdf]

BMJ Open is committed to open peer review. As part of this commitment we make the peer review history of every article we publish publicly available.

When an article is published we post the peer reviewers' comments and the authors' responses online. We also post the versions of the paper that were used during peer review. These are the versions that the peer review comments apply to.

The versions of the paper that follow are the versions that were submitted during the peer review process. They are not the versions of record or the final published versions. They should not be cited or distributed as the published version of this manuscript.

BMJ Open is an open access journal and the full, final, typeset and author-corrected version of record of the manuscript is available on our site with no access controls, subscription charges or pay-per-view fees (http://bmjopen.bmj.com).

If you have any questions on BMJ Open's open peer review process please email info.bmjopen@bmj.com

BMJ Open

Axial length to corneal radius of curvature ratio and refractive error in 4- to 6-year-old Chinese preschoolers: a cross-sectional study

Journal:	*BMJ Open*
Manuscript ID	bmjopen-2023-075115
Article Type:	Original research
Date Submitted by the Author:	04-May-2023
Complete List of Authors:	Tang, Tao; Peking University, Institute of Medical Technology<br>Zhao, Heng; Peking University, Institute of Medical Technology<br>Liu, Duanke; Peking University, Institute of Medical Technology<br>Li, Xuewei; Peking University, Institute of Medical Technology<br>Wang, Kai; Peking University People's Hospital, Department of Ophthalmology & Clinical Centre of Optometry, Peking University People's Hospital<br>Li, Yan; Peking University People's Hospital, Department of Ophthalmology & Clinical Centre of Optometry, Peking University People's Hospital<br>Zhao, Mingwei; Peking University People's Hospital, Department of Ophthalmology & Clinical Centre of Optometry, Peking University People's Hospital
Keywords:	OTOLARYNGOLOGY, Paediatric ophthalmology < OPHTHALMOLOGY, Medical ophthalmology < OPHTHALMOLOGY

SCHOLARONE™
Manuscripts

BMJ

I, the Submitting Author has the right to grant and does grant on behalf of all authors of the Work (as defined in the below author licence), an exclusive licence and/or a non-exclusive licence for contributions from authors who are: i) UK Crown employees; ii) where BMJ has agreed a CC-BY licence shall apply, and/or iii) in accordance with the terms applicable for US Federal Government officers or employees acting as part of their official duties; on a worldwide, perpetual, irrevocable, royalty-free basis to BMJ Publishing Group Ltd ("BMJ") its licensees and where the relevant Journal is co-owned by BMJ to the co-owners of the Journal, to publish the Work in this journal and any other BMJ products and to exploit all rights, as set out in our licence.

The Submitting Author accepts and understands that any supply made under these terms is made by BMJ to the Submitting Author unless you are acting as an employee on behalf of your employer or a postgraduate student of an affiliated institution which is paying any applicable article publishing charge ("APC") for Open Access articles. Where the Submitting Author wishes to make the Work available on an Open Access basis (and intends to pay the relevant APC), the terms of reuse of such Open Access shall be governed by a Creative Commons licence – details of these licences and which Creative Commons licence will apply to this Work are set out in our licence referred to above.

Other than as permitted in any relevant BMJ Author's Self Archiving Policies, I confirm this Work has not been accepted for publication elsewhere, is not being considered for publication elsewhere and does not duplicate material already published. I confirm all authors consent to publication of this Work and authorise the granting of this licence.

For peer review only

Title Page:

Axial length to corneal radius of curvature ratio and refractive error in 4- to 6-year-old Chinese preschoolers: a cross-sectional study

Tao Tang[1,2,3,4,5], Heng Zhao[1,2,3,4,5]

, Duanke Liu[1,2,3,4,5], Xuewei Li[1,2,3,4,5], Kai Wang[1,2,3,4,5*], Yan Li[2,3,4,5*], & Mingwei Zhao[1,2,3,4,5]

[1]Institute of Medical Technology, Peking University Health Science Center, Beijing, China

[2]Department of Ophthalmology & Clinical Centre of Optometry, Peking University People's Hospital, Beijing, China

[3]College of Optometry, Peking University Health Science Center, Beijing, China

[4]Eye Disease and Optometry Institute, Peking University People's Hospital, Beijing, China

[5]Beijing Key Laboratory of the Diagnosis and Therapy of Retinal and Choroid Diseases, Beijing, China

For peer review only

Author Affiliations:

First author: Tao Tang [1,2,3,4,5]

[1]Institute of Medical Technology, Peking University Health Science Center, Beijing, China

[2]Department of Ophthalmology & Clinical Centre of Optometry, Peking University People's Hospital, Beijing, China

[3]College of Optometry, Peking University Health Science Center, Beijing, China

[4]Eye Disease and Optometry Institute, Peking University People's Hospital, Beijing, China

[5]Beijing Key Laboratory of the Diagnosis and Therapy of Retinal and Choroid Diseases, Beijing, China

For peer review only - http://bmjopen.bmj.com/site/about/guidelines.xhtml

Heng Zhao [1, 2, 3, 4, 5]

[1]Institute of Medical Technology, Peking University Health Science Center, Beijing, China
[2]Department of Ophthalmology & Clinical Centre of Optometry, Peking University People's Hospital, Beijing, China
[3]College of Optometry, Peking University Health Science Center, Beijing, China
[4]Eye Disease and Optometry Institute, Peking University People's Hospital, Beijing, China
[5]Beijing Key Laboratory of the Diagnosis and Therapy of Retinal and Choroid Diseases, Beijing, China

Duanke Liu [1, 2, 3, 4, 5]

[1]Institute of Medical Technology, Peking University Health Science Center, Beijing, China
[2]Department of Ophthalmology & Clinical Centre of Optometry, Peking University People's Hospital, Beijing, China
[3]College of Optometry, Peking University Health Science Center, Beijing, China
[4]Eye Disease and Optometry Institute, Peking University People's Hospital, Beijing, China
[5]Beijing Key Laboratory of the Diagnosis and Therapy of Retinal and Choroid Diseases, Beijing, China

Xuewei Li [1, 2, 3, 4, 5]

[1]Institute of Medical Technology, Peking University Health Science Center, Beijing, China
[2]Department of Ophthalmology & Clinical Centre of Optometry, Peking University People's Hospital, Beijing, China
[3]College of Optometry, Peking University Health Science Center, Beijing, China
[4]Eye Disease and Optometry Institute, Peking University People's Hospital, Beijing, China
[5]Beijing Key Laboratory of the Diagnosis and Therapy of Retinal and Choroid Diseases, Beijing, China

Corresponding author: Kai Wang [1, 2, 3, 4, 5*]

For peer review only

[1]Institute of Medical Technology, Peking University Health Science Center, Beijing, China
[2]Department of Ophthalmology & Clinical Centre of Optometry, Peking University People's Hospital, Beijing, China
[3]College of Optometry, Peking University Health Science Center, Beijing, China
[4]Eye Disease and Optometry Institute, Peking University People's Hospital, Beijing, China
[5]Beijing Key Laboratory of the Diagnosis and Therapy of Retinal and Choroid Diseases, Beijing, China

Corresponding author: Yan Li [2,3,4,5 *]
[2]Department of Ophthalmology & Clinical Centre of Optometry, Peking University People's Hospital, Beijing, China
[3]College of Optometry, Peking University Health Science Center, Beijing, China
[4]Eye Disease and Optometry Institute, Peking University People's Hospital, Beijing, China
[5]Beijing Key Laboratory of the Diagnosis and Therapy of Retinal and Choroid Diseases, Beijing, China

Mingwei Zhao [1 , 2 , 3. 4 , 5]
[1]Institute of Medical Technology, Peking University Health Science Center, Beijing, China
[2]Department of Ophthalmology & Clinical Centre of Optometry, Peking University People's Hospital, Beijing, China
[3]College of Optometry, Peking University Health Science Center, Beijing, China
[4]Eye Disease and Optometry Institute, Peking University People's Hospital, Beijing, China
[5]Beijing Key Laboratory of the Diagnosis and Therapy of Retinal and Choroid Diseases, Beijing, China

Short Title: AL/CRC ratio and refractive error in Chinese preschoolers

[*]**Corresponding authors:**
Kai Wang, Department of Ophthalmology & Clinical Centre of Optometry, Peking University People' Hospital; College of Optometry, Peking University Health Science Center. Beijing, China.
E-mail: wang_kai@bjmu.edu.cn **Cell:** 0086-13810097881, **Tel.:** 0086-010-88325413.

For peer review only

Yan Li, Department of Ophthalmology & Clinical Centre of Optometry, Peking University People' Hospital; College of Optometry, Peking University Health Science Center. Beijing, China.
E-mail: 13801153660@163.com **Cell:** 0086-13801153660

Number of Table: three tables.
Number of Figure: two figures.
Number of Supplemental Table: two Supplemental Tables
Number of Supplemental Figure: one Supplemental Figure

Fundings: This work was supported by: Capital's Funds for Health Improvement and Research (No. 2022-1G-4083), National Natural Science Foundation of China (Grant Nos. 82171092 and 81870684), the National Key R&D Program of China (No.2020YFC2008200 and No.2021YFC2702100).

For peer review only

ABSTRACT

Objective To investigate the associations of axial length to corneal radius of curvature (AL/CRC) ratio with refractive error and to determine the effect of AL/CRC ratio on hyperopia reserve and myopia assessment among Chinese preschoolers.

Methods This was a cross-sectional study evaluated subjects aged 4-6 years. AL and CRC were obtained using a noncontact ocular biometer. Correlation analysis were performed to explore the associations of AL/CRC ratio with spherical equivalent refractive error (SER). The accuracy of AL/CRC ratio for hyperopia reserve and myopia assessment were analysed using cycloplegic refraction by drawing receiver operating characteristic (ROC) curves.

Results The analysis included 1024 participants (537 boys, 52.4%). The mean AL/CRC ratios in hyperopes, emmetropes and myopes were 2.90±0.06, 2.95±0.05 and 3.08±0.07, respectively. The SER was found to be more strongly correlated with AL/CRC ratio (ρ=-0.66, p<0.001) than either AL or CRC alone (ρ=-0.52, p<0.001; ρ=-0.03, p=0.33, respectively). AL/CRC was correlated with SER in hyperopes (ρ=-0.54, p<0.001), emmetropes (ρ=-0.33, p<0.001) and myopes (r=-0.67, p<0.001).

For low hyperopia reserve assessment, the area under the ROC curves of AL/CRC ratio was 0.861 (95% CI: 0.829-0.892), the optimal cut-off value of the AL/CRC ratio was ≥2.955. For myopia assessment, the area under the ROC curves of AL/CRC ratio was 0.954 (95% CI: 0.925-0.982), the optimal cut-off value of the AL/CRC ratio was ≥2.975.

Conclusions The SER showed a better correlation with AL/CRC ratio than either AL or CRC alone, especially in myopes, among 4- to 6-year-old children. These findings indicate that when cycloplegic refraction is unavailable, AL/CRC ratio can be used as an alternative indicator for monitoring hyperopia reserve and myopia progression among preschoolers, helping clinicians and parents screen children with low hyperopia reserve before primary school in a timely manner.

Key words: myopia, hyperopia reserve, spherical equivalent refractive error, axial length, axial length to corneal radius of curvature ratio

WHAT IS ALREADY KNOWN ON THIS TOPIC

Existing studies have addressed the correlation between the AL/CRC ratio and refractive error in school-aged children and adult population and found that SER showed a better correlation with the AL/CRC ratio than either AL or CRC alone. Few studies have evaluated the effect of the AL/CRC ratio on hyperopia reserve and myopia assessment among preschool children.

WHAT THIS STUDY ADDS

AL/CRC ratio was correlated with SER in hyperopes, emmetropes and myopes, while AL showed correlation with SER in hyperopes and myopes, but not in emmetropes. SER showed a better correlation with the AL/CRC ratio than either AL or CRC alone in different refractive statuses, especially in myopic children, among 4- to 6-year-old Chinese children. Therefore, AL/CRC ratio might be used as a reference for refractive development. For low hyperopia reserve assessment, the optimal cut-off value of the AL/CRC ratio was ≥2.955. For myopia assessment, the optimal cut-off value of the AL/CRC ratio was ≥2.975 among preschool children.

HOW THIS STUDY MIGHT AFFECT RESEARCH, PRACTICE OR POLICY

Clinicians and parents should be aware of children with low hyperopia reserve and monitor the development of refractive error before primary school. The application of the AL/CRC ratio reference as an indicator of refractive errors has the effect of simplifying the examination procedure and reducing unnecessary cycloplegia, especially when cycloplegic refraction is difficult to perform in 4- to 6-year-old children.

For peer review only

INTRODUCTION

Myopia has become a global public health problem worldwide, especially in East Asian countries such as China, Singapore and South Korea [1-3]. It is predicted that, by 2050, 49.8% of the global population will have myopia and 19.7% will suffer high myopia [4]. It is worth noting that the incidence of myopia has shown a trend toward increasing in those who are younger in recent years. In children aged 5-14 years old, the overall prevalence of myopia was 36.7% in Beijing [5]. In Hong Kong, myopia was 18.3% in children aged 6 years and increased to 61.5% in those aged 12 years [6]. In France, the myopic rate was 19.6% in children aged 0 to 9 years [7]. For most myopic patients, refractive error tends to stabilize in adulthood, while for a significant proportion of the myopic population, refraction continues to progress in adulthood and develops into pathologic myopia, resulting in various vision-threatening complications such as retinal detachment, glaucoma and cataracts [8-10]. Early monitoring of children's refractive development and timely control of myopia progression will reduce the risk of myopia-related complications in the future life.

Ocular biometrics including axial length, corneal curvature and lens power are among the most important factors affecting refractive status of the eyes [11]. During infancy and young childhood, most children have a physiological hyperopia of approximately +2.00 diopters (D), which is defined as hyperopia reserve [12,13]. Balancing changes in axial length and ocular refractive components, including the cornea and the lens, leads to emmetropization [14]. As children grow older, the refraction of the eyes become orthopedic, and the hyperopia reserve will gradually decrease. Myopia is an ocular disorder characterized by excessive ocular elongation in relation to total ocular power. It has been widely accepted that the myopia shift in refraction in schoolchildren is due to axial elongation [15,16]. When the rate of axial elongation outpaces the changes in optical power of the cornea and the lens, there is a tendency towards myopia onset [17]. For young children, an early reduction in hyperopic reserve might be a risk factor for future myopia development [18]. Zadnik et al. found that children in the United States in grade 1 with less than +0.75 D of hyperopia are at increased risk of myopia formation [18]. Yue et al. also reported that the cut point for myopia for grade 1 students was +0.31 D in the Chinese population [19]. Myopia develops fastest at the age of 6-7 years, and tends to slow down after 11 years [20]. Thus, to prevent the formation of myopia at an early age, clinicians and parents should pay attention to children with low hyperopia reserve and begin to monitor the development of refractive error before primary school.

Typically, cycloplegic refraction is the gold standard for paediatric refractive error diagnosis [21-23], especially in younger children [22,23], largely due to an active accommodation response. However, considering that the side effects of cycloplegia such as allergic conjunctivitis, photophobia and poor near visual acuity [24-26], cause parents or children to be reluctant to undergo cycloplegic refraction. Furthermore, for clinicians who lack regulatory approval for the use of cycloplegics and some patients who are unable to use cycloplegia, cycloplegic refraction may not be performed in such situations. Effective strategies are needed to examine ocular refractive development and diagnose refractive error in young children. Scheiman M et al. reported that the axial length to corneal radius of curvature (AL/CRC) ratio could be a better marker of myopia progression than AL alone [27]. The correlation between refraction and the AL/CRC ratio was stronger than that between refraction

For peer review only

and AL [27-29], and the AL/CRC ratio could explain the total variance in refractive error better than AL alone [30,31]. Moreover, the measurement of AL and CRC is noninvasive, and it is easily accepted by children [29]. Thus, although cycloplegic refraction cannot be replaced by any other measurements for the diagnosis of refractive error in children, as an alternative, the AL/CRC ratio can be used as an approximated surrogate for refractive error estimation.

Although many studies have addressed the correlation between the AL/CRC ratio and refractive error in school-aged children and adult population [17,32-35], few population-based studies to date have been performed to focus on the correlation between the AL/CRC ratio and refractive error and evaluate the effect of the AL/CRC ratio for hyperopia reserve and myopia assessment among preschool children in China. Therefore, in this study, we aimed to report the AL/CRC ratio in a Chinese preschool student population. In addition, we examined the correlation between the AL/CRC ratio and refractive error and evaluated the effectiveness of the AL/CRC ratio for refractive error assessment in 4- to 6-year-old Chinese preschoolers.

METHODS

Setting and participants

The study consisting of participants who visited the Peking University People's Hospital optometry centre between January 2020 and September 2022 due to refractive error, was conducted to investigate AL/CRC ratio and refractive error. All the subjects underwent a comprehensive ophthalmological examination and 1024 subjects fulfilled the following inclusion criteria were enrolled in the retrospective study: (1) age of 4 to 6 years old; (2) astigmatism and anisometropia of 1.50 D or less; (3) the monocular best corrected visual acuity (VA) of 0.00 logMAR (6/6) or better; (4) the absence of any ocular and systemic diseases, such as cataracts, glaucoma, and strabismus. This study was approved by the institutional research ethics committee of Peking University People's Hospital (2021PHB322-001). All examinations and management were performed in accordance with the Declaration of Helsinki, and written informed consent was obtained from the parents.

Measures

Refraction and ocular biometry examinations were performed for all subjects. First, measurement of ocular biometric parameters was performed with an optical biometer. AL and anterior chamber depth (ACD) was measured with noncontact partial-coherence laser interferometry (IOL Master; Carl Zeiss Meditec, Oberkochen, Germany). A corneal topography system (the Sirius, Italy) was used to obtain the mean K reading (Kmean) and ACD. Five valid readings were taken and averaged. The measurements were taken by the same experienced technical staff, and the mean value of these measurements was taken for statistical analysis. The ocular parameters presented here are the mean ± standard deviation (SD).

After the biometry measurements, all subjects underwent noncycloplegic refraction with an autorefractor (KP8800; Topcon Corp., Tokyo, Japan). This instrument generated five reliable readings of refraction in both eyes; the median reading was used for analysis. Then, cycloplegic refraction was performed for each subject with

For peer review only

three drops of 1% cyclopentolate, the first two drops were administered 5 minutes apart, and the third drop was administered 20 minutes later. Cycloplegia was considered complete if the pupil dilated to 6 mm or more and there was no pupillary reflex. The average of five readings automatically performed by the autorefractor was used for analyses. Slit-lamp examination and fundus examination were performed.

Definitions

Refraction was defined as spherical equivalent refraction (SER; SER = spherical power + cylinder power/2). Myopia was defined as SER<-0.50 D, emmetropia as –0.50 D≤SER≤+0.50 D and hyperopia as SER>+0.50 D. Signals from the tear film and retinal pigment epithelium (RPE) are used by noncontact partial-coherence laser interferometry (IOL Master; Carl Zeiss Meditec, Oberkochen, Germany) for axial length measurements. Anterior chamber depth was defined as the distance from the anterior corneal surface to the anterior lens surface. The mean K reading (Kmean = (flat K reading + steep K reading)/2) was the average of the steepest and flattest meridians. The corneal radius of curvature (CRC) was converted from the Kmean data using the formula CRC=0.3375/Kmean (D)×1000. The axial length to corneal radius of curvature ratio (AL/CRC ratio) was defined as the axial length divided by the mean corneal radius of curvature. Lens power was calculated using the Bennett-Rabbetts method [36] with unknown lens thickness, using measured values for SER, ACD, CRC and AL. The equation was as follows by the formula of Bennett.

$$P_{L,BR} = \frac{L(S_{cv} + K) - 1000n}{(L - ACD - c_{BR})\left(\frac{ACD + c_{BR}}{1000n}(S_{CV} + K) - 1\right)}$$

In this formula, $P_{L,BR}$ is the lens power using the Bennett-Rabbetts method, L is the axial length, S is the spherical equivalent refraction at the corneal vertex, $S_{CV}=S/(1-0.014S)$, K is the mean corneal power, n=1.336, and $c_{BR}=2.891\pm0.778$ mm.

Analysis

Statistical analysis was performed using the SPSS statistical software package (Version 22.0, IBM Corp., US). Descriptive statistics were calculated. Continuous data are expressed as the mean ± standard deviation (mean±SD). As biometric data for the right and left eyes were highly correlated (Pearson correlation coefficient, r=0.91), analyses were performed using data for the right eye only. The AL, CRC, AL/CRC ratio, and SER were analysed as continuous variables, and the Kolmogorov–Smirnov test was performed to verify the normality of these variables. To show the correlations between SER and AL, CRC, and the AL/CRC ratio, Spearman's correlation analyses were performed. The relationship between the AL/CRC ratio and other variables was assessed using Spearman's correlation. SER,

For peer review only - http://bmjopen.bmj.com/site/about/guidelines.xhtml

AL, CRC, AL/CRC ratio, ACD and LP were compared between groups using independent sample one-way ANOVA. Separate linear regression models were constructed with SER as the dependent variable and AL/CRC ratio, AL, and CRC as the main covariate, respectively. The sensitivity, specificity, Youden index, true positive rate, and false positive rate of the AL/CRC ratio and AL for hyperopia reserve and myopia assessment were analysed. The effectiveness of the AL/CR ratio, CRC and AL for hyperopia reserve and myopia assessment was evaluated using receiver operating characteristic (ROC) curves and the area under the curve (AUC) of the ROC curves. All p values were two sided, and a p value of < 0.05 was indicated statistical significance.

RESULTS

Demographic characteristics

A total of 1024 children (537 boys and 487 girls) aged 4-6 years (mean 5.77±0.47 years) were included in this study. The average ages of the boys and girls were 5.79 and 5.76 years, respectively. There was no significant difference in sex distribution (χ^2 = 2.441, p=0.118). The mean SER was 0.90±1.11 D, mean AL was 22.59±0.76 mm, mean CRC was 7.74±0.24 mm and mean AL/CR ratio was 2.92±0.08. The detailed demographic characteristics of the subjects are shown in **Supplemental Table 1**.

The histograms of SER, AL, CRC and AL/CRC ratio are shown in **Supplemental Figure 1**. The Kolmogorov–Smirnov test indicated that the frequency distribution was nonnormal for SER, AL, CRC and the AL/CRC ratio (all p<0.05). The overall prevalence rates of myopia and high myopia were 7.3% and 0.1%, respectively. The prevalence rates of myopia in subjects aged 4, 5, and 6 years were 4.2% (1/24), 5.4% (10/186), and 7.9% (64/814), respectively.

Table 1 shows the distribution of ocular biometry and the AL/CRC ratio according to the categories of refractive status of subjects. Axial length was significantly longer (p<0.001) in myopes (23.74 ± 0.65 mm) than in emmetropes (22.86 ± 0.63 mm) and hyperopes (22.42 ± 0.67 mm) (p<0.001). The AL/CRC ratio was also significantly higher in myopes (3.08±0.07) than in emmetropes (2.95±0.05) and hyperopes (2.90±0.06) (p<0.001), while CRC and ACD tended to stabilize in different refractive statuses. Emmetropes have the largest LP, while myopes have the smallest LP.

Table 1. Values of ocular parameters and correlations between AL, CRC, AL/CRC ratio and SER according to refractive status

For peer review only

Parameters	Hyperopes (n=772)	Emmetropes (n=174)	Myopes (n=78)	p value
SER	+1.32±0.73	+0.18±0.30	-1.70±1.05	<0.001
AL	22.42±0.67	22.86±0.63	23.74±0.65	<0.001
CRC	7.74±0.24	7.76±0.26	7.72±0.21	0.678
AL/CRC	2.90±0.06	2.95±0.05	3.08±0.07	<0.001
ACD	3.35±0.27	3.46±0.23	3.30±0.26	0.140
LP	24.18±1.27	24.31±1.32	22.62±1.28	<0.001
Spearman's correlation coefficients				
SER and AL	-0.41*	-0.08	-0.37*	
SER and CRC	-0.06	0.10	-0.15	
SER and AL/CRC	-0.54*	-0.33*	-0.67*	

SER, spherical equivalent refractive error; AL, axial length; CRC, corneal radius of curvature; AL/CRC ratio, axial length to corneal radius of curvature ratio; LP, lens power; ACD, anterior chamber depth; D, diopter. Significant differences are masked by asterisks.

Correlation analysis between AL, CRC, AL/CRC ratio and SER

Figure 1 shows the scatter plots between AL, CRC, AL/CRC ratio and SER. The SER showed a better correlation with the AL/CRC ratio (Spearman's correlation coefficient, ρ=-0.66, p<0.001) than either AL (ρ=-0.52, p<0.001) or CRC (ρ=-0.03, p=0.33) (**Table 2**). The Spearman's correlation coefficient between SER and the

For peer review only

AL/CRC ratio was the highest in myopes (ρ=-0.67, $p<0.001$), followed by hyperopes (ρ=-0.54, $p<0.001$) and emmetropes (ρ=-0.33, $p<0.001$). There was a significant correlation between SER and AL in hyperopes (ρ=-0.41, $p<0.001$) and myopes (ρ=-0.37, $p<0.001$), but not in emmetropes (ρ=-0.08, p=0.28). There was no statistical significance for the correlation between SER and CRC in all refractive groups (all $p>0.05$). Compared to either AL or CRC, the AL/CRC ratio showed better correlation with SER in all refractive groups (**Table 1**).

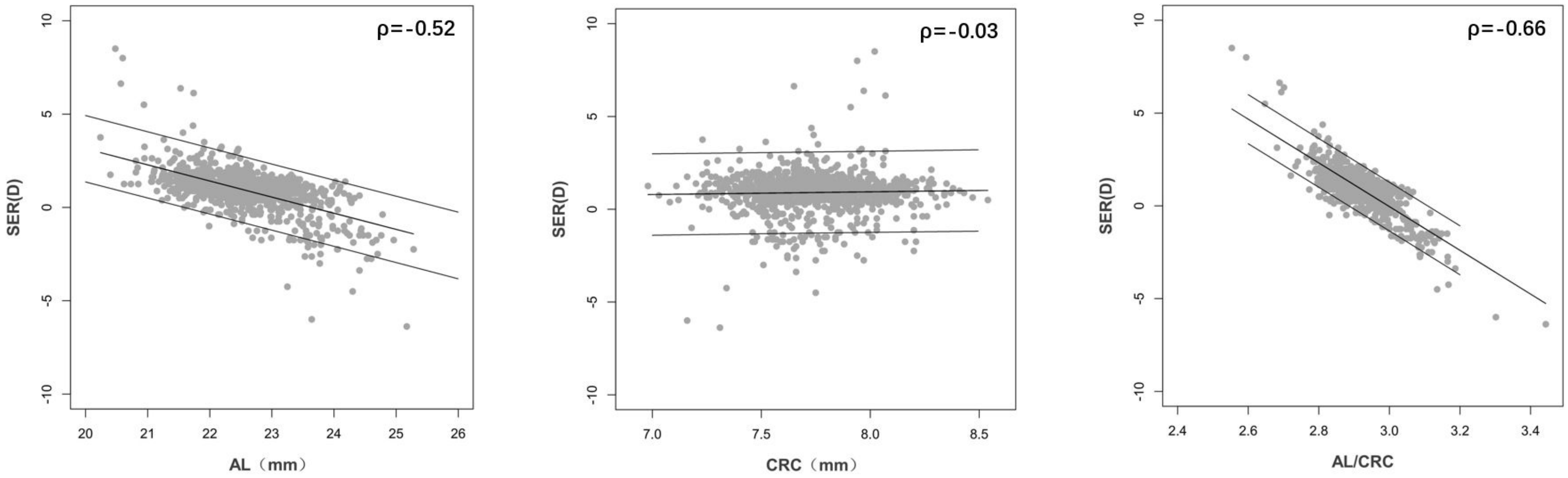

Figure 1. Correlation analysis between AL, CRC, AL/CRC ratio and SER. SER, spherical equivalent refractive error; AL, axial length; CRC, corneal radius of curvature; AL/CRC ratio, axial length to corneal radius of curvature ratio, D, diopter.

Regression analysis between AL, CRC, AL/CRC ratio and SER

Univariate and multiple linear regression analyses were performed in this study (**Table 2**). For SER variance, AL explained 34.5%, whereas the AL/CRC ratio explained 64.5% of the data after adjusting for age and sex. Multiple linear regression adjusted for age and sex showed that a 0.1 increase in the AL/CRC ratio was associated with a -1.19 D change in SER and a 1 mm increase in AL was associated with a -0.87 D change in SER.

Table 2. Association between AL/CRC ratio, AL, and CRC with SER.

Variables	Correlation coefficient	P value	Univariate OR (95% CI)	P value	Gender and age-adjusted Multivariate OR (95% CI)				
					Unstandardised coefficient	95% CI	Standardised coefficient	P value	R2
AL	-0.52	<0.001	-0.86 (-0.94 to -0.79)	<0.001	-0.869	-0.942 to -0.795	-0.590	<0.001	0.345
CRC	-0.03	0.33	0.15 (-0.14 to 0.43)	0.31	0.148	-0.135 to 0.430	0.032	0.306	0.002
AL/CRC	-0.66	<0.001	-11.73 (-12.27 to -11.19)	<0.001	-11.876	-12.418 to -11.334	-0.809	<0.001	0.645

SER, spherical equivalent refractive error; AL, axial length; CRC, corneal radius of curvature; AL/CRC ratio, axial length to corneal radius of curvature ratio. OR, odds ratio.

Effectiveness of AL, CRC and the AL/CR ratio for hyperopia reserve assessment and myopia assessment

Taking cycloplegic refraction SER ≤ +0.50 D as the standard for diagnosis of the lack of hyperopia reserve [37], the effectiveness of AL, CRC and AL/CR ratio for myopia assessment were analysed (**Figure 2A**). The ROC curves were drawn using AL, CRC and the AL/CR ratio as the index for hyperopia reserve assessment, and the AUCs of the ROC curves were 0.777 (95% confidence interval, CI: 0.741-0.812), 0.494(95% CI: 0.450-0.539) and 0.861 (95% CI: 0.829-0.892), respectively. If AL was used for hyperopia reserve assessment, the optimal cut-off point of the ROC curve was ≥22.615 mm, with a specificity, sensitivity, Youden index, true positive rate, and false positive rate of 0.785, 0.604, 0.389, 0.604 and 0.215, respectively. If the AL/CR ratio was used for myopia assessment, the optimal cut-off point of the ROC curve was ≥2.955, with a specificity, sensitivity, Youden index, true positive rate, and false positive rate of 0.732, 0.863, 0.595, 0.863, and 0.268, respectively (**Table 3**).

For peer review only

Taking cycloplegic refraction SER ≤ −0.50 D as the gold standard for diagnosis of myopia [19], the effectiveness of AL, CRC and AL/CR ratio for myopia assessment were analysed (**Figure 2B**). The ROC curves were drawn using AL, CRC and the AL/CR ratio as the index for myopia assessment, and the AUCs of the ROC curves were 0.898 (95% CI: 0.864-0.932), 0.493(95% CI: 0.434-0.553) and 0.954 (95% CI: 0.925-0.982), respectively. If AL was used for myopia assessment, the optimal cut-off point of the ROC curve was ≥23.235 mm, with a specificity, sensitivity, Youden index, true positive rate, and false positive rate of 0.862, 0.800, 0.662, 0.800 and 0.138, respectively. If the AL/CR ratio was used for myopia assessment, the optimal cut-off point of the ROC curve was ≥2.975, with a specificity, sensitivity, Youden index, true positive rate, and false positive rate of 0.883, 0.894, 0.777, 0.894, and 0.117, respectively (**Table 3**).

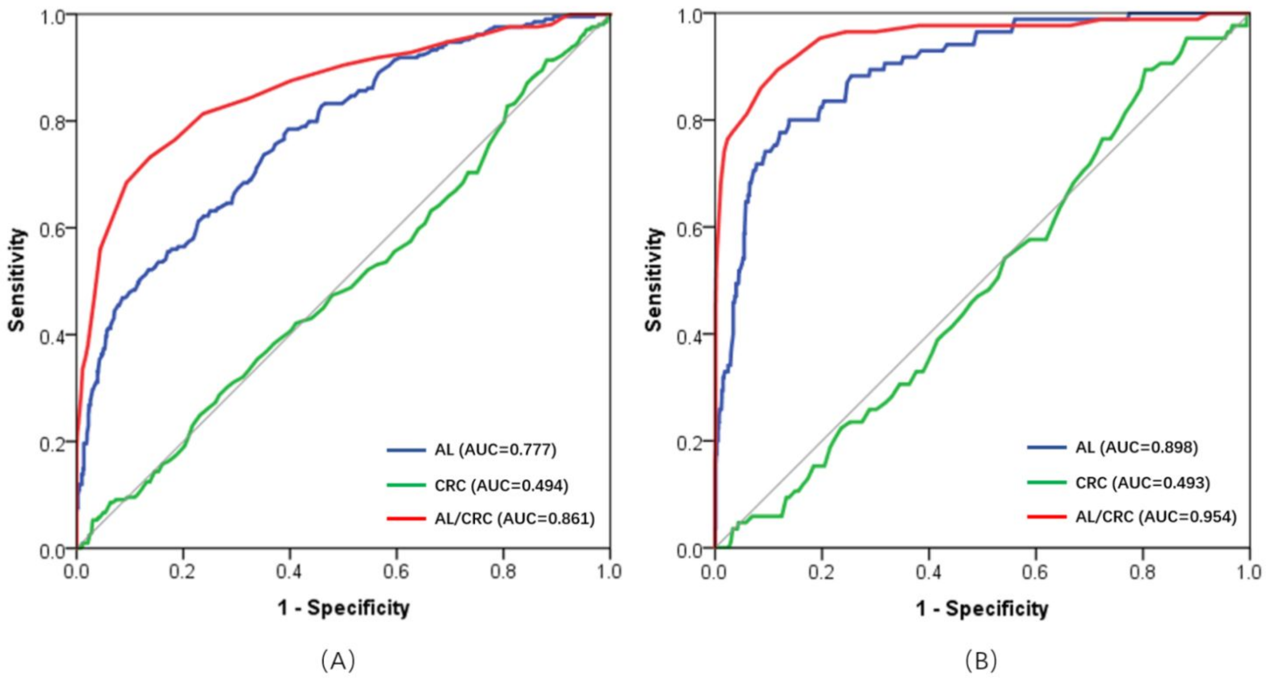

Figure 2. Comparison of the effectiveness of AL, CRC and the AL/CR ratio for hyperopia reserve assessment (A) and myopia assessment (B). AL, axial length; CRC, corneal radius of curvature; AL/CRC ratio, axial length to corneal radius of curvature ratio; AUC, area under curve.

Table 3. The sensitivity, specificity, and Youden index of AL and the AL/CRC ratio for hyperopia reserve assessment and myopia assessment.

	Criterion	Cut-off value	Sensitivity	Specificity	Youden index
Hyperopia reserve assessment	AL (mm)	22.615	0.785	0.604	0.389
	AL/CRC ratio	2.955	0.732	0.863	0.595
Myopia assessment	AL (mm)	23.235	0.800	0.862	0.662
	AL/CRC ratio	2.975	0.894	0.883	0.777

AL, axial length; AL/CRC ratio, axial length to corneal radius of curvature ratio.

DISCCUSION

This study found that AL/CRC ratio was correlated with SER in hyperopes, emmetropes and myopes, while AL showed correlation with SER in hyperopes and myopes, but not in emmetropes. The SER showed a better correlation with the AL/CRC ratio than either AL or CRC alone in different refractive statuses, especially in myopes, among 4- to 6-year-old children. The effect of the AL/CR ratio for low hyperopia reserve and myopia assessment was higher than that of either AL or CRC alone. These findings indicate that when cycloplegic refraction is unavailable, AL/CRC ratio can be used as an alternative indicator for monitoring low hyperopia reserve and myopia progression among preschoolers.

Myopia is a common disorder of the eyes and has become a global public health concern [38,39]. During the past several decades, the prevalence of myopia in children has increased rapidly, and the age at onset of myopia has decreased. Chinese children tend to have a higher prevalence of myopia than children in other countries and of other ethnicities. The prevalence of myopia exceeds 60% among 12 years of age in China after primary school, reaches nearly 80% at 16 years of age after junior high school, and surpasses 90% in university students [1,4,13]. Notably, the incidence of myopia increased among preschool children in recent years. The prevalence of myopia was 17.6% in children aged 6 years in Hong Kong [40]. A study in Shanghai of 7166 Chinese children aged 4 to 6 years reported that the prevalence of myopia ranged from 5.7% to 7.1% [41]. In this study, the prevalence of myopia in preschoolers increased from 4.2% at 4 years of age to 7.9% at 6 years of age. Although myopia tends to stabilize after adulthood, children with a younger age at myopia onset are prone to develop high myopia and have a high risk of suffering from vision-threatening complications. In view of this condition, it is urgent to carry out early vision screening and refractive error assessment among preschool children. Ocular biometrics have been used to estimate the progression of refractive error [29,34]. The AL/CRC ratio summarizes the overall relationship between AL and CRC and minimizes variability in AL and CRC, thus, it has been considered as a more robust index to evaluate the refractive status of the eye [17]. Moreover, the AL/CRC ratio also correlates with refractive error more strongly than AL or CRC alone [28,31]. Therefore, the AL/CRC ratio could be useful in

For peer review only

determining refractive error and monitoring myopia progression.

Changes in AL and CRC are important ocular biometrics affecting refractive status [34]. Previous studies have indicated that CRC may be actively modulated to regulate refractive development, and the onset of myopia is due to a failure of corneal compensation for axial elongation [11,33]. In this study, we found that there was no correlation between CRC and refractive error, while AL, or more strikingly, the AL/CRC ratio had a significant correlation with refractive error. This finding suggests that AL has a larger effect on refractive error than CRC. Grosvenor et al. was the first to report the correlation between the AL/CRC ratio and refractive error and studied the role of the AL/CRC ratio in determining the refractive state of the eye [42]. They demonstrated that the AL/CRC ratio was the most important biometric factor in myopia. Some researchers further confirmed Grosvenor's findings [29,43,44]. The distribution of AL/CRC ratio has been reported in different countries and age populations. A summary of AL/CRC ratio in other studies is shown in **Supplemental Table 2**. The average values reported for the AL/CRC ratio range from 2.81 to 3.13. Differences in age, race, included subjects, measurement methods and techniques may account for the disagreement in the various studies. The AL/CRC ratio has been proposed as a calculated biometric parameter that correlates better with SER than AL, and the AL/CRC ratio could explain the total variance in SER better than AL alone [31]. This is because the AL/CRC ratio provides a rough measure of the degree of matching between AL and corneal power. In this study, we also found a better correlation between the AL/CRC ratio and SER in children aged 4-6 years compared to that of AL or CRC alone, especially in children with myopia. It is possibly suggested that the AL/CRC ratio is a more useful marker of progress towards myopic refractions than absolute ocular biometric values such as AL or CRC. Moreover, the value of the AL/CRC ratio was negatively correlated with SER. As demonstrated in this study, the AL/CRC ratio was significantly higher in myopes than in emmetropes and hyperopes, which was consistent with studies performed by Foo et al. and Guo et al. [28,31]. This finding indicates that the greater the myopic refraction, the larger the AL/CRC ratio.

A previous study reported that a high AL/CRC ratio of no less than 3.0 has been considered an indicator for the onset of myopia [42]. High axial length to corneal radius ratio is a risk factor in the development of myopia. When the AL/CRC ratio was higher than 3.0, it was considered that other ocular parameters were not able to compensate for the change in refraction caused by the elongation of AL, thereby inducing myopia. As shown in this study, the area under the ROC of the AL/CR ratio for myopia assessment was 0.954, and the optimal cut-off value is ≥2.975, which was smaller than that in the study performed by Mu et al. [29]. This result indicates that for children aged 4 to 6 years, the risk of myopia is likely to occur when the AL/CRC ratio is greater than 2.975. We also conducted an evaluation of the effectiveness of the AL/CRC ratio, CRC and AL for hyperopia reserve assessment in this population. Previous study has reported that hyperopia reserve is a potential indicator of myopia [19]. There is a progressive shift in mean refraction from roughly + 2.00 D at 3 months of age to approximately + 1.00 D at 6.5 years of age [45]. Then emmetropization continues at a slower rate after 6 years of age. Zadnik et al. revealed that children in the United States in grade 1 with less than +0.75 D of hyperopia are at increased risk of myopia onset [18]. Yue et al. also reported that the cut point for myopia for grade 1 students was +0.31 D in the Chinese population [19]. Li et al.'s [41] findings showed that the 5-year cumulative incidence of myopia was 4.6%, 26.3%, 52.3%, 78.6%, 92.6% and 94.3%, respectively, corresponding to students

with a baseline hyperopia reserve of >+2.00 D, +1.50 D to +2.00 D, +1.00 D to +1.50 D, +0.50 D to +1.00 D, 0.00 D to +0.50 D and -0.50 D to 0.00 D, respectively. The lower the hyperopia reserve in young children, the higher the incidence of myopia onset. In our study, taking cycloplegic refraction SER ≤ +0.50 D as the standard for diagnosis of the lack of hyperopia reserve, the area under the ROC curve of the AL/CR ratio for the lack of hyperopia reserve assessment was greater than that of AL and CRC and the optimal cut-off value of the AL/CRC ratio was ≥2.955. Thus, clinicians and parents can monitor the hyperopia reserve of young children by regularly measuring the axial length and corneal curvature when cycloplegic refraction is unavailable. When the AL/CRC ratio is greater than 2.955 among preschool children, clinicians and parents should pay attention to the children with less hyperopia reserve and begin to take measures to prevent myopia formation.

In the present study, AL alone contributed 34.5% of the variance in SER, whereas the AL/CR ratio accounted for 64.5%. Guo et al. study [31] found that AL alone contributed 18.6% of the variance in SER, whereas the AL/CR ratio accounted for 39.8% in the study population of preschoolers aged 3-6 years old. He et al. [30] reported that AL/CR ratio and AL explained 66.4% and 43.1% of the variance in SER, respectively, in a group of Chinese children 6 to 12 years of age. This suggested that the AL/CRC ratio is more closely related to refractive error than AL or CRC alone. Moreover, we found a linear increase in AL/CRC ratio from hyperopia towards myopia. The corresponding increase in SER of -1.1 9D with every 0.1 increase in the AL/CRC ratio in this study is larger than -0.74 D reported by researchers in Singapore [28] and -0.89D reported among the Nigerian population [33], but slightly lower than -1.21 D reported among northern Iranian subjects [17]. The above findings revealed that in addition to AL and CRC, there are other potential factors that influence SER results. Thus, the AL/CRC ratio cannot replace cycloplegic refraction in determining refractive error in children. However, cycloplegic refraction is easily influenced by the cooperation and compliance of the examinees, especially in younger children, which is not feasible for large-scale myopia assessment and long-term refractive development follow-up. For this reason, the AL/CRC ratio could be used as an alternative indicator for refraction estimation, and it may be more accurate than AL. In addition, measurement of AL and CRC and determination of the AL/CRC ratio are relatively easy and can be conducted by a technician using noncontact, objective, and automated instruments. The application of the AL/CRC ratio reference as an indicator of refractive errors has the effect of simplifying the examination procedure and reducing unnecessary cycloplegia, especially when cycloplegic refraction is difficult to perform in 4- to 6-year-old children.

There were some limitations in this study. First, because this study is cross-sectional and does not pinpoint the temporal correlations between refractive error and the AL/CRC ratio, it is impossible to rule out the possibility of observational and inclusionary biases due to the retrospective study design. Admittedly, longitudinal studies would be needed to conduct to evaluate intraindividual discrepancy. In addition, the data from Beijing may not be representative for the rural areas and other regions in China. Last, the sample size of the 4-year-old participants (N=24) was relatively small. Although our findings are applicable to this population as well, there is potential for discrepancy. We will further increase the sample size of this population to increase the accuracy and reliability of the results of this study.

In conclusion, our study found that SER showed a better correlation with the AL/CRC ratio than either AL or CRC alone in different refractive statuses, especially in myopic children, among 4- to 6-year-old Chinese children. The AL/CRC ratio could explain the total variance in refractive error better than AL alone. The effect of

For peer review only

the AL/CR ratio for assessing the lack of hyperopia reserve and for the myopia assessment was higher than that of AL and CRC. These findings indicate that when cycloplegic refraction is unavailable, the AL/CRC ratio can be used as a reference for refractive development and an alternative indicator for monitoring hyperopia reserve and myopia progression among preschoolers, helping clinicians and parents screen children with low hyperopia reserve before primary school in a timely manner. In the future, longitudinal cohort designs will be conducted for optimal study of the effect of the AL/CRC ratio on hyperopia reserve and myopia assessment in preschool children.

For peer review only

Declarations

Authors' information:

Author and Affiliations

[1]Institute of Medical Technology, Peking University Health Science Center, Beijing, China

[2]Department of Ophthalmology & Clinical Centre of Optometry, Peking University People's Hospital, Beijing, China

[3]College of Optometry, Peking University Health Science Center, Beijing, China

[4]Eye Disease and Optometry Institute, Peking University People's Hospital, Beijing, China

[5]Beijing Key Laboratory of the Diagnosis and Therapy of Retinal and Choroid Diseases, Beijing, China

Tao Tang[1,2,3,4,5], Heng Zhao[1,2,3,4,5], Duanke Liu[1,2,3,4,5], Xuewei Li[1,2,3,4,5], Kai Wang[1,2,3,4,5]*, Yan Li [2,3,4,5]*, & Mingwei Zhao[1,2,3,4,5]

Authors' contributions

T.T. developed the concept, collected data, performed the experiments and data analysis, wrote the main manuscript text and prepared Figures 1-4 and Tables 1-5; Z.H. collected data and prepared Figure 3; L.D.K. and L.X.W collected data; W.K. and L.Y. developed the concept and designed the experiments; Z.M.W. developed the study conception. All authors reviewed the manuscript.

Funding

This work was supported by: Capital's Funds for Health Improvement and Research (No. 2022-1G-4083), National Natural Science Foundation of China (Grant Nos. 82171092 and 81870684), and the National Key R&D Program of China (No.2020YFC2008200 and No.2021YFC2702100).

Competing interests

The authors declare that they have no conflicts of interest.

Patient consent for publication

Not applicable.

Ethics approval

This study was approved by the institutional research ethics committee of Peking University People's Hospital (2021PHB322-001). All examinations and management were performed in accordance with the Declaration of Helsinki, and written informed consent was obtained from the parents.

Availability of data and materials

All data generated or analysed during this study are included in this published article.

Acknowledgements

None.

Corresponding author

Correspondence to Kai Wang and Yan Li

Data Availability Statement

All data generated or analysed during this study are included in this article and its online supplementary material. Further inquiries can be directed to the corresponding author.

References

1. Ma Y, Qu X, Zhu X, et al. Age-Specific Prevalence of Visual Impairment and Refractive Error in Children Aged 3-10 Years in Shanghai, China. Invest

For peer review only

Ophthalmol Vis Sci. 2016;57(14):6188-6196.
2. Koh V, Yang A, Saw SM, et al. Differences in Prevalence of Refractive Errors in Young Asian Males in Singapore between 1996–1997 and 2009–2010. Ophthalmic Epidemiology. 2014;21(4):247-255.
3. Jung SK, Lee JH, Kakizaki H, Jee D. Prevalence of myopia and its association with body stature and educational level in 19-year-old male conscripts in seoul, South Korea. Invest Ophthalmol Vis Sci. 2012 Aug 15;53(9):5579-83.
4. Holden BA, Fricke TR, Wilson DA, et al. Global Prevalence of Myopia and High Myopia and Temporal Trends from 2000 through 2050. Ophthalmology. 2016;123(5):1036-1042.
5. Lyu Y, Zhang H, Gong Y, et al. Prevalence of and factors associated with myopia in primary school students in the Chaoyang District of Beijing, China. Jpn J Ophthalmol. 2015;59(6):421-429.
6. Choy BNK, You Q, Zhu MM, Lai JSM, Ng ALK, Wong IYH. Prevalence and associations of myopia in Hong Kong primary school students. Jpn J Ophthalmol. 2020;64(4):437-449.
7. Matamoros E, Ingrand P, Pelen F, et al. Prevalence of Myopia in France: A Cross-Sectional Analysis. Medicine (Baltimore). 2015;94(45):e1976.
8. Lee JY, Sung KR, Han S, Na JH. Effect of myopia on the progression of primary open-angle glaucoma. Invest Ophthalmol Vis Sci. 2015;56(3):1775-1781.
9. Coppe AM, Ripandelli G, Parisi V, Varano M, Stirpe M. Prevalence of asymptomatic macular holes in highly myopic eyes. Ophthalmology. 2005;112(12):2103-2109.
10. Haarman AEG, Enthoven CA, Tideman JWL, Tedja MS, Verhoeven VJM, Klaver CCW. The Complications of Myopia: A Review and Meta-Analysis. Invest Ophthalmol Vis Sci. 2020;61(4):49.
11. Mutti DO, Mitchell GL, Jones LA, et al. Axial growth and changes in lenticular and corneal power during emmetropization in infants. Invest Ophthalmol Vis Sci. 2005;46(9):3074-3080.
12. Flitcroft DI. Emmetropisation and the aetiology of refractive errors. Eye (Lond). 2014;28(2):169-179.
13. Song Y, Zhu S, Yang B, et al. Accommodation and binocular vision changes after wearing orthokeratology lens in 8- to 14-year-old myopic children. Graefes Arch Clin Exp Ophthalmol. 2021;259(7):2035-2045.
14. Rozema J, Dankert S, Iribarren R. Emmetropization and nonmyopic eye growth. Surv Ophthalmol. 2023.
15. Ojaimi E, Rose KA, Morgan IG, et al. Distribution of ocular biometric parameters and refraction in a population-based study of Australian children. Invest Ophthalmol Vis Sci. 2005;46(8):2748-2754.
16. Ip JM, Huynh SC, Kifley A, et al. Variation of the contribution from axial length and other oculometric parameters to refraction by age and ethnicity. Invest

For peer review only

Ophthalmol Vis Sci. 2007;48(10):4846-4853.

17. Hashemi H, Khabazkhoob M, Miraftab M, Emamian MH, Shariati M, Abdolahi-Nia T, et al. Axial length to corneal radius of curvature ratio and refractive errors. J Ophthalmic Vis Res. 2013 Jul;8(3):220-6.

18. Zadnik K, Sinnott LT, Cotter SA, et al. Prediction of Juvenile-Onset Myopia. JAMA Ophthalmol. 2015;133(6):683-689.

19. Yue Y, Liu X, Yi S, Liu B, Yi H, Li H. High prevalence of myopia and low hyperopia reserve in 4411 Chinese primary school students and associated risk factors. BMC Ophthalmol. 2022;22(1):212.

20. Hyman L, Gwiazda J, Hussein M, Norton TT, Wang Y, Marsh-Tootle W, et al. Relationship of age, sex, and ethnicity with myopia progression and axial elongation in the correction of myopia evaluation trial. Arch Ophthalmol. 2005 Jul;123(7):977-87.

21. Morgan IG, Iribarren R, Fotouhi A, Grzybowski A. Cycloplegic refraction is the gold standard for epidemiological studies. Acta Ophthalmol. 2015;93(6):581-585.

22. Li T, Zhou X, Zhu J, Tang X, Gu X. Effect of cycloplegia on the measurement of refractive error in Chinese children. Clin Exp Optom. 2019;102(2):160-165.

23. Hu YY, Wu JF, Lu TL, et al. Effect of cycloplegia on the refractive status of children: the Shandong children eye study. PLoS One. 2015;10(2):e0117482.

24. Chia A, Chua WH, Cheung YB, et al. Atropine for the treatment of childhood myopia: safety and efficacy of 0.5%, 0.1%, and 0.01% doses (Atropine for the Treatment of Myopia 2). Ophthalmology. 2012;119(2):347-354.

25. Gong Q, Janowski M, Luo M, et al. Efficacy and Adverse Effects of Atropine in Childhood Myopia: A Meta-analysis. JAMA Ophthalmol. 2017;135(6):624-630.

26. Wei S, Li SM, An W, et al. Safety and Efficacy of Low-Dose Atropine Eyedrops for the Treatment of Myopia Progression in Chinese Children: A Randomized Clinical Trial. JAMA Ophthalmol. 2020;138(11):1178-1184.

27. Scheiman M, Gwiazda J, Zhang Q, et al. Longitudinal changes in corneal curvature and its relationship to axial length in the Correction of Myopia Evaluation Trial (COMET) cohort. J Optom. 2016;9(1):13-21.

28. Foo VH, Verkicharla PK, Ikram MK, Chua SY, Cai S, Tan CS, et al. Axial Length/Corneal Radius of Curvature Ratio and Myopia in 3-Year-Old Children. Transl Vis Sci Technol. 2016 Feb 9;5(1):5.

29. Mu J, Zeng D, Fan J, et al. The accuracy of the axial length and axial length/corneal radius ratio for myopia assessment among Chinese children. Front Pediatr. 2022;10:859944.

30. He X, Zou H, Lu L, et al. Axial length/corneal radius ratio: association with refractive state and role on myopia detection combined with visual acuity in Chinese schoolchildren. PLoS One. 2015;10(2):e0111766.

For peer review only

31. Guo X, Fu M, Ding X, Morgan IG, Zeng Y, He M. Significant Axial Elongation with Minimal Change in Refraction in 3- to 6-Year-Old Chinese Preschoolers: The Shenzhen Kindergarten Eye Study. Ophthalmology. 2017;124(12):1826-1838.
32. Jong M, Sankaridurg P, Naduvilath TJ, Li W, He M. The Relationship between Progression in Axial Length/Corneal Radius of Curvature Ratio and Spherical Equivalent Refractive Error in Myopia. Optom Vis Sci. 2018;95(10):921-929.
33. Badmus SA, Ajaiyeoba AI, Adegbehingbe BO, Onakpoya OH, Adeoye AO. Axial length/corneal radius of curvature ratio and refractive status in an adult Nigerian population. Niger J Clin Pract. 2017;20(10):1328-1334.
34. Iyamu E, Iyamu J, Obiakor CI. The role of axial length-corneal radius of curvature ratio in refractive state categorization in a nigerian population. ISRN Ophthalmol. 2011;2011:138941.
35. Warrier S, Wu HM, Newland HS, et al. Ocular biometry and determinants of refractive error in rural Myanmar: the Meiktila Eye Study. Br J Ophthalmol. 2008;92(12):1591-1594.
36. Rozema JJ, Atchison DA, Tassignon MJ. Comparing methods to estimate the human lens power. Invest Ophthalmol Vis Sci. 2011;52(11):7937-7942.
37. Li SM, Wei S, Atchison DA, et al. Annual Incidences and Progressions of Myopia and High Myopia in Chinese Schoolchildren Based on a 5-Year Cohort Study. Invest Ophthalmol Vis Sci. 2022;63(1):8.
38. Foster PJ, Jiang Y. Epidemiology of myopia. Eye (Lond). 2014;28(2):202-208.
39. Dolgin E. The myopia boom. Nature. 2015;519(7543):276-8.
40. Lam CS, Lam CH, Cheng SC, Chan LY. Prevalence of myopia among Hong Kong Chinese schoolchildren: changes over two decades. Ophthalmic Physiol Opt. 2012;32(1):17-24.
41. Li T, Zhou X, Chen X, Qi H, Gao Q. Refractive Error in Chinese Preschool Children: The Shanghai Study. Eye Contact Lens. 2019;45(3):182-187.
42. Grosvenor T. High axial length/corneal radius ratio as a risk factor in the development of myopia. Am J Optom Physiol Opt. 1988 Sep;65(9):689-96.
43. Matsumura S, Dannoue K, Kawakami M, et al. Prevalence of Myopia and Its Associated Factors Among Japanese Preschool Children. Front Public Health. 2022;10:901480.
44. González Blanco F, Sanz Fernández JC, Muńoz Sanz MA. Axial length, corneal radius, and age of myopia onset. Optom Vis Sci. 2008 Feb;85(2):89-96.
45. Mutti DO, Sinnott LT, Lynn Mitchell G, et al. Ocular Component Development during Infancy and Early Childhood. Optom Vis Sci. 2018;95(11):976-985.

For peer review only

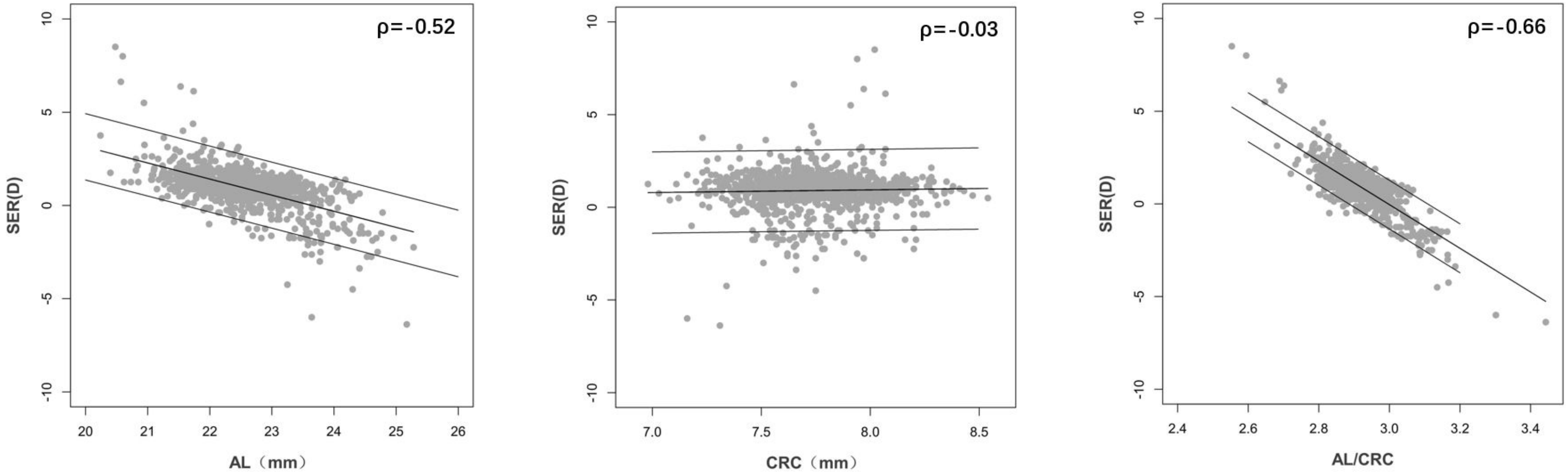

Figure 1. Correlation analysis between AL, CRC, AL/CRC ratio and SER. SER, spherical equivalent refractive error; AL, axial length; CRC, corneal radius of curvature; AL/CRC ratio, axial length to corneal radius of curvature ratio, D, diopter.

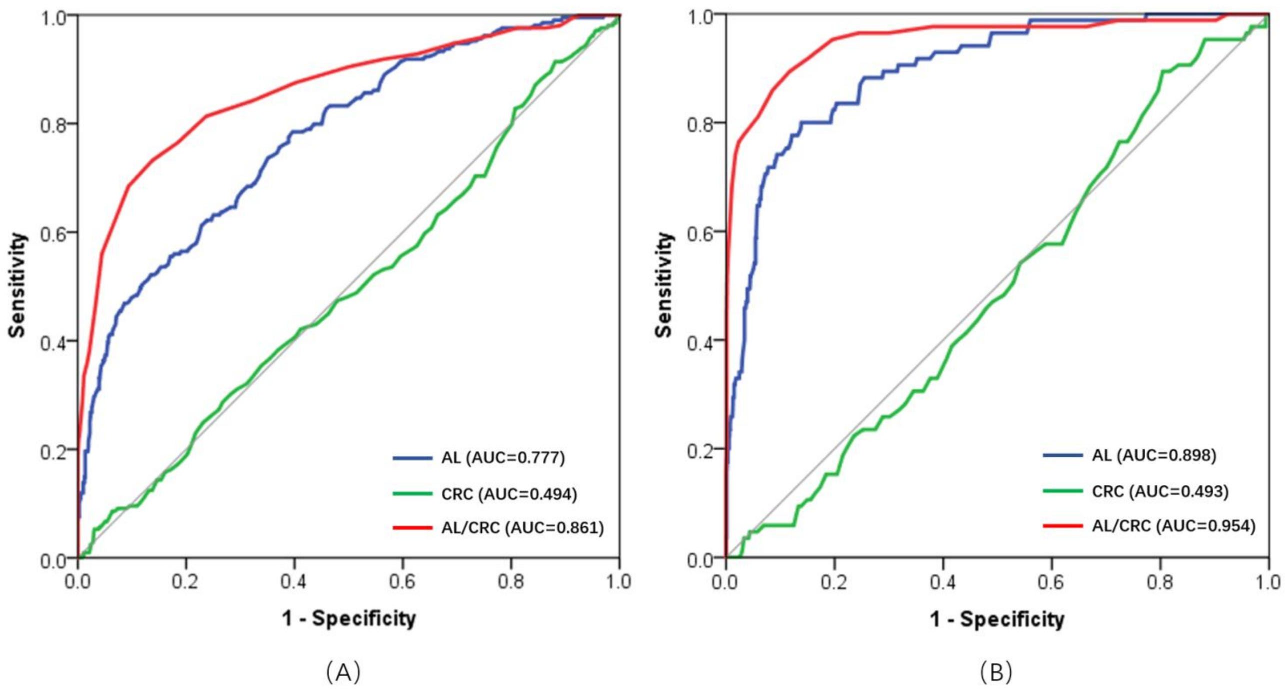

Figure 2. Comparison of the effectiveness of AL, CRC and the AL/CR ratio for hyperopia reserve assessment (A) and myopia assessment (B). AL, axial length; CRC, corneal radius of curvature; AL/CRC ratio, axial length to corneal radius of curvature ratio; AUC, area under curve.

For peer review only - http://bmjopen.bmj.com/site/about/guidelines.xhtml

Table 1. Values of ocular parameters and correlations between AL, CRC, AL/CRC ratio and SER according to refractive status

Parameters	Hyperopes (n=772)	Emmetropes (n=174)	Myopes (n=78)	p value
SER	+1.32±0.73	+0.18±0.30	-1.70±1.05	<0.001
AL	22.42±0.67	22.86±0.63	23.74±0.65	<0.001
CRC	7.74±0.24	7.76±0.26	7.72±0.21	0.678
AL/CRC	2.90±0.06	2.95±0.05	3.08±0.07	<0.001
ACD	3.35±0.27	3.46±0.23	3.30±0.26	0.140
LP	24.18±1.27	24.31±1.32	22.62±1.28	<0.001
Spearman's correlation coefficients				
SER and AL	-0.41*	-0.08	-0.37*	
SER and CRC	-0.06	0.10	-0.15	
SER and AL/CRC	-0.54*	-0.33*	-0.67*	

SER, spherical equivalent refractive error; AL, axial length; CRC, corneal radius of curvature; AL/CRC ratio, axial length to corneal radius of curvature ratio; LP, lens power; ACD, anterior chamber depth; D, diopter. Significant differences are masked by asterisks.

For peer review only

Table 2. Association between AL/CRC ratio, AL, and CRC with SER.

Variables	Correlation coefficient	P value	Univariate OR (95% CI)	P value	Gender and age-adjusted Multivariate OR (95% CI)				
					Unstandardised coefficient	95% CI	Standardised coefficient	P value	R^2
AL	-0.52	<0.001	-0.86 (-0.94 to -0.79)	<0.001	-0.869	-0.942 to -0.795	-0.590	<0.001	0.345
CRC	-0.03	0.33	0.15 (-0.14 to 0.43)	0.31	0.148	-0.135 to 0.430	0.032	0.306	0.002
AL/CRC	-0.66	<0.001	-11.73 (-12.27 to -11.19)	<0.001	-11.876	-12.418 to -11.334	-0.809	<0.001	0.645

SER, spherical equivalent refractive error; AL, axial length; CRC, corneal radius of curvature; AL/CRC ratio, axial length to corneal radius of curvature ratio. OR, odds ratio.

For peer review only

Table 3. The sensitivity, specificity, and Youden index of AL and the AL/CRC ratio for hyperopia reserve assessment and myopia assessment.

	Criterion	Cut-off value	Sensitivity	Specificity	Youden index
Hyperopia reserve assessment	AL (mm)	22.615	0.785	0.604	0.389
	AL/CRC ratio	2.955	0.732	0.863	0.595
Myopia assessment	AL (mm)	23.235	0.800	0.862	0.662
	AL/CRC ratio	2.975	0.894	0.883	0.777

AL, axial length; AL/CRC ratio, axial length to corneal radius of curvature ratio.

For peer review only

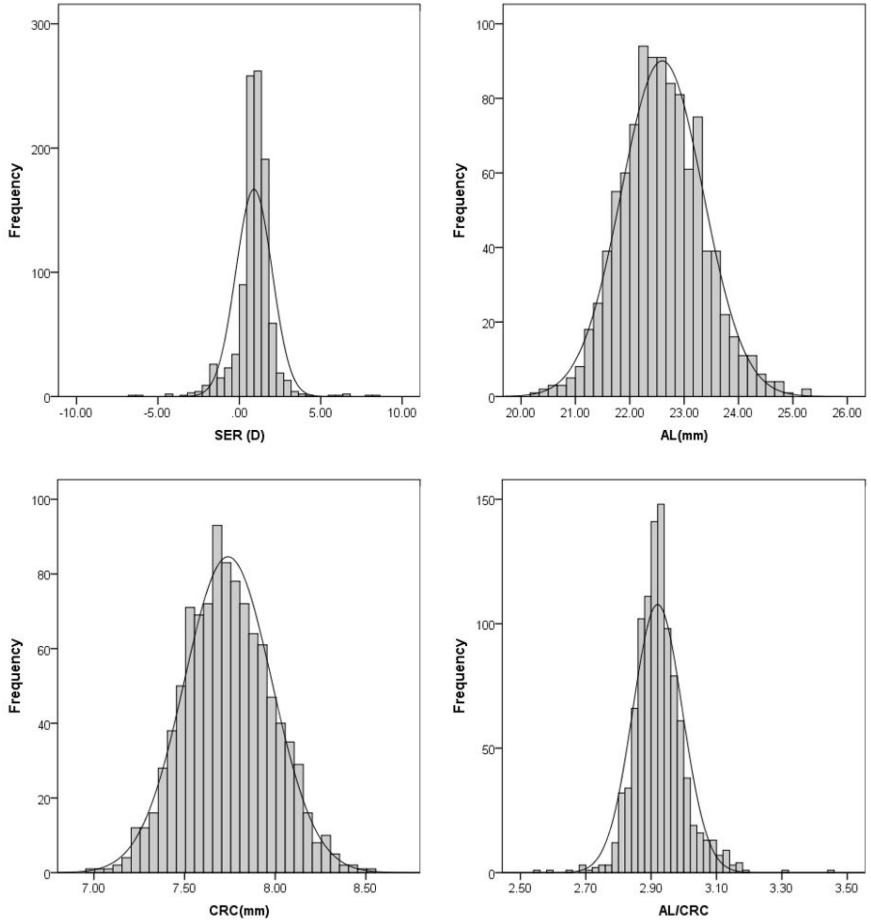

Supplemental Figure 1. Histograms showing distributions of SER, AL, CRC and AL/CRC ratio. SER, spherical equivalent refractive error; AL, axial length; CRC, corneal radius of curvature; AL/CRC ratio, axial length to corneal radius of curvature ratio; D, diopter.

Supplemental Table 1. Demographic and biometric data of included participants

Parameters	Total, N=1024		Boys, N=537	Girls, N=487	P value
	Mean±SD	Range	Mean±SD	Mean±SD	
Age (years)	5.77±0.47	4-6	5.85±0.35	5.68±0.56	<0.001
SER (D)	0.90±1.11	-6.38 to 8.50	0.90±1.09	0.90±1.13	0.922
AL (mm)	22.59±0.76	20.24 to 25.28	22.62±0.77	22.57±0.74	0.350
CRC (mm)	7.74±0.24	6.98 to 8.54	7.74±0.24	7.74±0.24	0.805
AL/CRC	2.92±0.08	2.65 to 3.17	2.92±0.08	2.92±0.08	0.141
LP (D)	24.09±1.35	19.68 to 28.68	24.02±1.34	24.16±1.35	0.117
ACD (mm)	3.37±0.27	2.09 to 5.37	3.38±0.27	3.34±0.27	0.028

SER, spherical equivalent refractive error; AL, axial length; CRC, corneal radius of curvature; AL/CRC, axial length to corneal radius of curvature ratio; LP, lens power; ACD, anterior chamber depth; D, diopter.

For peer review only

Supplemental Table 2. Summary of studies reporting the AL, CRC, and AL/CRC ratio.

Origin	N	Age (years)	SER (D)	AL/CRC	AL	CRC
China [19]	4411	7-10	-0.13	2.97	23.27	7.82
Singapore [28]	349	3	+0.88	2.81	21.73	7.72
Nigerian [34]	70	20-39	-0.24	3.03	23.74	7.84
Iran [17]	4820	40-64	-	3.033	23.14	7.63
Japan [43]	457	4-6	0.13	2.88	22.35	7.76
Australia [15]	1765	5.5-8.4	+1.26	2.906	22.61	7.70
Myanmar [35]	1498	40-70	-1.22	2.99	22.76	7.62
Current study	1024	4-6	+0.90	2.92	22.59	7.74

SER, spherical equivalent refractive error; AL, axial length; CRC, corneal radius of curvature; AL/CRC ratio, axial length to corneal radius of curvature ratio.

For peer review only

STROBE Statement—Checklist of items that should be included in reports of ***cross-sectional studies***

	Item No	Recommendation	Page No
**Title and abstract**	1	(*a*) Indicate the study's design with a commonly used term in the title or the abstract	P1
		(*b*) Provide in the abstract an informative and balanced summary of what was done and what was found	P4-5
**Introduction**			
Background/rationale	2	Explain the scientific background and rationale for the investigation being reported	P5-6
Objectives	3	State specific objectives, including any prespecified hypotheses	P6
**Methods**			
Study design	4	Present key elements of study design early in the paper	P6
Setting	5	Describe the setting, locations, and relevant dates, including periods of recruitment, exposure, follow-up, and data collection	P6-7
Participants	6	(*a*) Give the eligibility criteria, and the sources and methods of selection of participants	P6-7
Variables	7	Clearly define all outcomes, exposures, predictors, potential confounders, and effect modifiers. Give diagnostic criteria, if applicable	P7
Data sources/ measurement	8*	For each variable of interest, give sources of data and details of methods of assessment (measurement). Describe comparability of assessment methods if there is more than one group	*P7*
Bias	9	Describe any efforts to address potential sources of bias	P7
Study size	10	Explain how the study size was arrived at	P7
Quantitative variables	11	Explain how quantitative variables were handled in the analyses. If applicable, describe which groupings were chosen and why	P7
Statistical methods	12	(*a*) Describe all statistical methods, including those used to control for confounding	P8
		(*b*) Describe any methods used to examine subgroups and interactions	
		(*c*) Explain how missing data were addressed	P8
		(*d*) If applicable, describe analytical methods taking account of sampling strategy	
		(*e*) Describe any sensitivity analyses	
**Results**			
Participants	13*	(a) Report numbers of individuals at each stage of study—eg numbers potentially eligible, examined for eligibility, confirmed eligible, included in the study, completing follow-up, and analysed	P8
		(b) Give reasons for non-participation at each stage	
		(c) Consider use of a flow diagram	
Descriptive data	14*	(a) Give characteristics of study participants (eg demographic, clinical, social) and information on exposures and potential confounders	P8
		(b) Indicate number of participants with missing data for each variable of interest	
Outcome data	15*	Report numbers of outcome events or summary measures	P8
Main results	16	(*a*) Give unadjusted estimates and, if applicable, confounder-adjusted estimates and their precision (eg, 95% confidence interval). Make clear which confounders were adjusted for and why they were included	P8-14

For peer review only

		(*b*) Report category boundaries when continuous variables were categorized	
		(*c*) If relevant, consider translating estimates of relative risk into absolute risk for a meaningful time period	
Other analyses	17	Report other analyses done—eg analyses of subgroups and interactions, and sensitivity analyses	
**Discussion**			
Key results	18	Summarise key results with reference to study objectives	P15-17
Limitations	19	Discuss limitations of the study, taking into account sources of potential bias or imprecision. Discuss both direction and magnitude of any potential bias	P17-18
Interpretation	20	Give a cautious overall interpretation of results considering objectives, limitations, multiplicity of analyses, results from similar studies, and other relevant evidence	P18
Generalisability	21	Discuss the generalisability (external validity) of the study results	P18
**Other information**			
Funding	22	Give the source of funding and the role of the funders for the present study and, if applicable, for the original study on which the present article is based	P19

*Give information separately for exposed and unexposed groups.

Note: An Explanation and Elaboration article discusses each checklist item and gives methodological background and published examples of transparent reporting. The STROBE checklist is best used in conjunction with this article (freely available on the Web sites of PLoS Medicine at http://www.plosmedicine.org/, Annals of Internal Medicine at http://www.annals.org/, and Epidemiology at http://www.epidem.com/). Information on the STROBE Initiative is available at www.strobe-statement.org.

For peer review only

Axial length to corneal radius of curvature ratio and refractive error in 4- to 6-year-old Chinese preschoolers: a retrospective cross-sectional study

Journal:	*BMJ Open*
Manuscript ID	bmjopen-2023-075115.R1
Article Type:	Original research
Date Submitted by the Author:	20-Oct-2023
Complete List of Authors:	Tang, Tao; Peking University, Institute of Medical Technology<br>Zhao, Heng; Peking University, Institute of Medical Technology<br>Liu, Duanke; Peking University, Institute of Medical Technology<br>Li, Xuewei; Peking University, Institute of Medical Technology<br>Wang, Kai; Peking University People's Hospital, Department of Ophthalmology & Clinical Centre of Optometry, Peking University People's Hospital<br>Li, Yan; Peking University People's Hospital, Department of Ophthalmology & Clinical Centre of Optometry, Peking University People's Hospital<br>Zhao, Mingwei; Peking University People's Hospital, Department of Ophthalmology & Clinical Centre of Optometry, Peking University People's Hospital
**Primary Subject Heading**:	Ophthalmology
Secondary Subject Heading:	Paediatrics, Public health
Keywords:	OTOLARYNGOLOGY, Paediatric ophthalmology < OPHTHALMOLOGY, Medical ophthalmology < OPHTHALMOLOGY

SCHOLARONE™
Manuscripts

I, the Submitting Author has the right to grant and does grant on behalf of all authors of the Work (as defined in the below author licence), an exclusive licence and/or a non-exclusive licence for contributions from authors who are: i) UK Crown employees; ii) where BMJ has agreed a CC-BY licence shall apply, and/or iii) in accordance with the terms applicable for US Federal Government officers or employees acting as part of their official duties; on a worldwide, perpetual, irrevocable, royalty-free basis to BMJ Publishing Group Ltd ("BMJ") its licensees and where the relevant Journal is co-owned by BMJ to the co-owners of the Journal, to publish the Work in this journal and any other BMJ products and to exploit all rights, as set out in our licence.

The Submitting Author accepts and understands that any supply made under these terms is made by BMJ to the Submitting Author unless you are acting as an employee on behalf of your employer or a postgraduate student of an affiliated institution which is paying any applicable article publishing charge ("APC") for Open Access articles. Where the Submitting Author wishes to make the Work available on an Open Access basis (and intends to pay the relevant APC), the terms of reuse of such Open Access shall be governed by a Creative Commons licence – details of these licences and which Creative Commons licence will apply to this Work are set out in our licence referred to above.

Other than as permitted in any relevant BMJ Author's Self Archiving Policies, I confirm this Work has not been accepted for publication elsewhere, is not being considered for publication elsewhere and does not duplicate material already published. I confirm all authors consent to publication of this Work and authorise the granting of this licence.

For peer review only

Title Page:

Axial length to corneal radius of curvature ratio and refractive error in 4- to 6-year-old Chinese preschoolers: a retrospective cross-sectional study

Tao Tang[1, 2, 3.4, 5], Heng Zhao[1, 2, 3.4, 5]

, Duanke Liu[1, 2, 3.4, 5], Xuewei Li[1, 2, 3.4, 5], Kai Wang[1, 2, 3.4, 5 *], Yan Li [2,3,4,5 *], & Mingwei Zhao[1, 2, 3.4, 5]

[1]Institute of Medical Technology, Peking University Health Science Center, Beijing, China

[2]Department of Ophthalmology & Clinical Centre of Optometry, Peking University People's Hospital, Beijing, China

[3]College of Optometry, Peking University Health Science Center, Beijing, China

[4]Eye Disease and Optometry Institute, Peking University People's Hospital, Beijing, China

[5]Beijing Key Laboratory of the Diagnosis and Therapy of Retinal and Choroid Diseases, Beijing, China

For peer review only

Author Affiliations:

First author: Tao Tang [1, 2, 3.4, 5]

[1]Institute of Medical Technology, Peking University Health Science Center, Beijing, China

[2]Department of Ophthalmology & Clinical Centre of Optometry, Peking University People's Hospital, Beijing, China

[3]College of Optometry, Peking University Health Science Center, Beijing, China

[4]Eye Disease and Optometry Institute, Peking University People's Hospital, Beijing, China

[5]Beijing Key Laboratory of the Diagnosis and Therapy of Retinal and Choroid Diseases, Beijing, China

Heng Zhao [1,2,3.4,5]

[1]Institute of Medical Technology, Peking University Health Science Center, Beijing, China
[2]Department of Ophthalmology & Clinical Centre of Optometry, Peking University People's Hospital, Beijing, China
[3]College of Optometry, Peking University Health Science Center, Beijing, China
[4]Eye Disease and Optometry Institute, Peking University People's Hospital, Beijing, China
[5]Beijing Key Laboratory of the Diagnosis and Therapy of Retinal and Choroid Diseases, Beijing, China

Duanke Liu [1,2,3.4,5]

[1]Institute of Medical Technology, Peking University Health Science Center, Beijing, China
[2]Department of Ophthalmology & Clinical Centre of Optometry, Peking University People's Hospital, Beijing, China
[3]College of Optometry, Peking University Health Science Center, Beijing, China
[4]Eye Disease and Optometry Institute, Peking University People's Hospital, Beijing, China
[5]Beijing Key Laboratory of the Diagnosis and Therapy of Retinal and Choroid Diseases, Beijing, China

Xuewei Li [1,2,3.4,5]

[1]Institute of Medical Technology, Peking University Health Science Center, Beijing, China
[2]Department of Ophthalmology & Clinical Centre of Optometry, Peking University People's Hospital, Beijing, China
[3]College of Optometry, Peking University Health Science Center, Beijing, China
[4]Eye Disease and Optometry Institute, Peking University People's Hospital, Beijing, China
[5]Beijing Key Laboratory of the Diagnosis and Therapy of Retinal and Choroid Diseases, Beijing, China

Corresponding author: Kai Wang [1,2,3.4,5*]

For peer review only

[1]Institute of Medical Technology, Peking University Health Science Center, Beijing, China
[2]Department of Ophthalmology & Clinical Centre of Optometry, Peking University People's Hospital, Beijing, China
[3]College of Optometry, Peking University Health Science Center, Beijing, China
[4]Eye Disease and Optometry Institute, Peking University People's Hospital, Beijing, China
[5]Beijing Key Laboratory of the Diagnosis and Therapy of Retinal and Choroid Diseases, Beijing, China

Corresponding author: Yan Li [2,3,4,5 *]
[2]Department of Ophthalmology & Clinical Centre of Optometry, Peking University People's Hospital, Beijing, China
[3]College of Optometry, Peking University Health Science Center, Beijing, China
[4]Eye Disease and Optometry Institute, Peking University People's Hospital, Beijing, China
[5]Beijing Key Laboratory of the Diagnosis and Therapy of Retinal and Choroid Diseases, Beijing, China

Mingwei Zhao [1, 2, 3. 4, 5]
[1]Institute of Medical Technology, Peking University Health Science Center, Beijing, China
[2]Department of Ophthalmology & Clinical Centre of Optometry, Peking University People's Hospital, Beijing, China
[3]College of Optometry, Peking University Health Science Center, Beijing, China
[4]Eye Disease and Optometry Institute, Peking University People's Hospital, Beijing, China
[5]Beijing Key Laboratory of the Diagnosis and Therapy of Retinal and Choroid Diseases, Beijing, China

Short Title: AL/CRC ratio and refractive error in Chinese preschoolers

[*]**Corresponding authors:**
Kai Wang, Department of Ophthalmology & Clinical Centre of Optometry, Peking University People' Hospital; College of Optometry, Peking University Health Science Center. Beijing, China.
E-mail: wang_kai@bjmu.edu.cn **Cell:** 0086-13810097881, **Tel.:** 0086-010-88325413.

For peer review only

Yan Li, Department of Ophthalmology & Clinical Centre of Optometry, Peking University People' Hospital; College of Optometry, Peking University Health Science Center. Beijing, China.
E-mail: 13801153660@163.com **Cell:** 0086-13801153660

Number of Table: three tables.
Number of Figure: two figures.
Number of Supplemental Table: three Supplemental Tables
Number of Supplemental Figure: two Supplemental Figure

ABSTRACT

Objective To investigate the associations of axial length to corneal radius of curvature (AL/CRC) ratio with refractive error and to determine the effect of AL/CRC ratio on hyperopia reserve and myopia assessment among Chinese preschoolers.

Methods This was a retrospective cross-sectional study evaluated subjects aged 4-6 years. AL and CRC were obtained using a noncontact ocular biometer. Correlation analysis was performed to explore the associations of AL/CRC ratio with spherical equivalent refractive error (SER). The accuracy of AL/CRC ratio for hyperopia reserve and myopia assessment were analysed using cycloplegic refraction by drawing receiver operating characteristic (ROC) curves.

Results The analysis included 1024 participants (537 boys, 52.4%). The mean AL/CRC ratios in hyperopes, emmetropes and myopes were 2.90±0.06, 2.95±0.05 and 3.08±0.07, respectively. The SER was found to be more strongly correlated with AL/CRC ratio (ρ=-0.66, p<0.001) than either AL or CRC alone (ρ=-0.52, p<0.001; ρ=-0.03, p=0.33, respectively). AL/CRC was correlated with SER in hyperopes (ρ=-0.54, p<0.001), emmetropes (ρ=-0.33, p<0.001) and myopes (r=-0.67, p<0.001).

For peer review only

For low hyperopia reserve assessment, the area under the ROC curves of AL/CRC ratio was 0.861 (95% CI: 0.829-0.892), the optimal cut-off value of the AL/CRC ratio was ≥2.955. For myopia assessment, the area under the ROC curves of AL/CRC ratio was 0.954 (95% CI: 0.925-0.982), the optimal cut-off value of the AL/CRC ratio was ≥2.975.

Conclusions The SER showed a better correlation with AL/CRC ratio than either AL or CRC alone, especially in myopes, among 4- to 6-year-old children. These findings indicate that when cycloplegic refraction is unavailable, AL/CRC ratio could be used as an alternative indicator for identifying low hyperopia reserve and myopia among preschoolers, helping clinicians and parents screen children with low hyperopia reserve before primary school in a timely manner.

Strengths and limitations of this study

1. An advantage of this study was the population-based design, with a large sample size of 4- to 6-year-old children.
2. The hyperopic reserve and myopia were assessed by AL/CRC ratio using ROC curves and AUC.
3. This study is cross-sectional and does not pinpoint the temporal correlations between refractive error and the AL/CRC ratio.
4. The sample size of 4-year-old participants was relatively small, which may result in potential for discrepancy.

Key words: myopia, hyperopia reserve, spherical equivalent refractive error, axial length, axial length to corneal radius of curvature ratio

INTRODUCTION

Myopia has become a global public health problem worldwide, especially in East Asian countries such as China, Singapore and South Korea [1-3]. It is predicted that, by 2050, 49.8% of the global population will have myopia and 19.7% will suffer high myopia [4]. It is worth noting that the incidence of myopia has shown a trend toward increasing in those who are younger in recent years. In 2011, the overall prevalence of myopia among children aged 5-14 years in Beijing was 36.7% [5]. In Hong Kong, myopia was 18.3% in children aged 6 years and increased to 61.5% in those aged 12 years in 2018 [6]. In France, the myopic rate was 19.6% in children aged 0 to 9 years in 2013 [7]. For most myopic patients, refractive error tends to stabilize in adulthood, while for a significant proportion of the myopic population, refraction continues to progress in adulthood and develops into pathologic myopia, resulting in various vision-threatening complications such as retinal detachment, glaucoma and cataracts [8-10]. Early monitoring of children’s refractive development and timely control of myopia progression will reduce the risk of myopia-related

For peer review only

complications in the future life.

Ocular biometrics including axial length, corneal curvature and lens power are among the most important factors affecting refractive status of the eyes [11]. During infancy and young childhood, most children have a physiological hyperopia of approximately +2.00 diopters (D), which is defined as hyperopia reserve [12,13]. Balancing changes in axial length and ocular refractive components, including the cornea and the lens, leads to emmetropization [14]. As children grow older, the refraction of the eye develops, and the hyperopia reserve will gradually decrease. Myopia is an ocular disorder characterized by excessive ocular elongation in relation to total ocular power. It has been widely accepted that the myopia shift in refraction in schoolchildren is due to axial elongation [15,16]. When the rate of axial elongation outpaces the changes in optical power of the cornea and the lens, there is a tendency towards myopia onset [17]. For young children, an early reduction in hyperopic reserve might be a risk factor for future myopia development [18]. Zadnik et al. found that children in the United States in grade 1 with less than +0.75 D of hyperopia are at increased risk of myopia formation [18]. Yue et al. also reported that the cut point for myopia for grade 1 students was +0.31 D in the Chinese population [19]. Myopia develops fastest at the age of 6-7 years, and tends to slow down after 11 years [20]. Thus, to prevent the formation of myopia at an early age, clinicians and parents should pay attention to children with low hyperopia reserve and begin to monitor the development of refractive error before primary school.

Typically, cycloplegic refraction is the gold standard for paediatric refractive error diagnosis [21-23], especially in younger children [22,23], largely due to an active accommodation response. However, considering that the side effects of cycloplegia such as allergic conjunctivitis, photophobia and poor near visual acuity [24-26], cause parents or children to be reluctant to undergo cycloplegic refraction. Furthermore, for clinicians who lack regulatory approval for the use of cycloplegics and some patients who are unable to use cycloplegia, cycloplegic refraction may not be performed in such situations. Effective strategies are needed to examine ocular refractive development and diagnose refractive error in young children. Scheiman M et al. reported that the axial length to corneal radius of curvature (AL/CRC) ratio could be a better marker of myopia progression than AL alone [27]. The correlation between refraction and the AL/CRC ratio was stronger than that between refraction and AL [27-29], and the AL/CRC ratio could explain the total variance in refractive error better than AL alone [30,31]. Moreover, the measurement of AL and CRC is noninvasive, and it is easily accepted by children [29]. Thus, although cycloplegic refraction cannot be replaced by any other measurements for the diagnosis of refractive error in children, as an alternative, the AL/CRC ratio can be used as an approximated surrogate for refractive error estimation.

Although many studies have addressed the correlation between the AL/CRC ratio and refractive error in school-aged children and adult population [17,32-35], few population-based studies to date have been performed to focus on the correlation between the AL/CRC ratio and refractive error and evaluate the effect of the AL/CRC ratio for hyperopia reserve and myopia assessment among preschool children in China. Therefore, in this study, we aimed to report the AL/CRC ratio in a Chinese preschool student population. In addition, we examined the correlation between the AL/CRC ratio and refractive error and evaluated the effectiveness of the AL/CRC ratio for refractive error assessment in 4- to 6-year-old Chinese preschoolers.

For peer review only

For peer review only - http://bmjopen.bmj.com/site/about/guidelines.xhtml

METHODS

Setting and participants

The study consisting of participants who visited the Peking University People's Hospital optometry centre between January 2020 and September 2022 due to refractive error, was conducted to investigate AL/CRC ratio and refractive error. All the subjects underwent a comprehensive ophthalmological examination and 1024 subjects fulfilled the following inclusion criteria were enrolled in the retrospective cross-sectional study: (1) age of 4 to 6 years old; (2) astigmatism and anisometropia of 1.50 D or less; (3) the monocular best corrected visual acuity (VA) of 0.00 logMAR (6/6) or better; (4) the absence of any ocular and systemic diseases, such as cataracts, glaucoma, and strabismus. This study was approved by the institutional research ethics committee of Peking University People's Hospital (2021PHB322-001). All examinations and management were performed in accordance with the Declaration of Helsinki, and written informed consent was obtained from the parents.

Measures

Refraction and ocular biometry examinations were performed for all subjects. First, measurement of ocular biometric parameters was performed with an optical biometer. AL was measured with noncontact partial-coherence laser interferometry (IOL Master; Carl Zeiss Meditec, Oberkochen, Germany). A corneal topography system (the Sirius, Italy) was used to obtain the mean K reading (Kmean) and anterior chamber depth (ACD). Five valid readings were taken and averaged. The measurements were taken by the same experienced technical staff, and the mean value of these measurements was taken for statistical analysis. The ocular parameters presented here are the mean ± standard deviation (SD).

After the biometry measurements, all subjects underwent noncycloplegic refraction with an autorefractor (KP8800; Topcon Corp., Tokyo, Japan). This instrument generated five reliable readings of refraction in both eyes; the median reading was used for analysis. Then, cycloplegic refraction was performed for each subject with three drops of 1% cyclopentolate, the first two drops were administered 5 minutes apart, and the third drop was administered 20 minutes later. Cycloplegia was considered complete if the pupil dilated to 6 mm or more and there was no pupillary reflex. The average of five readings automatically performed by the autorefractor was used for analyses. Slit-lamp examination and fundus examination were performed.

Definitions

Refraction was defined as spherical equivalent refraction (SER; SER = spherical power + cylinder power/2). Myopia was defined as SER$\leq$-0.50 D, emmetropia as –0.50 D$<$SER$\leq$+0.50 D and hyperopia as SER$>$+0.50 D. Signals from the tear film and retinal pigment epithelium (RPE) are used by noncontact partial-coherence laser interferometry (IOL Master; Carl Zeiss Meditec, Oberkochen, Germany) for axial length measurements. Anterior chamber depth was defined as the distance from the anterior corneal surface to the anterior lens surface. The mean K reading (Kmean = (flat K reading + steep K reading)/2) was the average of the steepest and flattest meridians. The corneal radius of curvature (CRC) was converted from the Kmean data using the formula CRC=0.3375/Kmean (D)×1000. The axial length to corneal

For peer review only

radius of curvature ratio (AL/CRC ratio) was defined as the axial length divided by the mean corneal radius of curvature. Lens power was calculated using the Bennett-Rabbetts method [36] with unknown lens thickness, using measured values for SER, ACD, CRC and AL. The equation was as follows by the formula of Bennett.

$$LP = \frac{L(S_{cv} + K) - 1000n}{(L - ACD - c_{BR})\left(\frac{ACD + c_{BR}}{1000n}(S_{CV} + K) - 1\right)}$$

In this formula, LP is the lens power using the Bennett-Rabbetts method, L is the axial length, S is the spherical equivalent refraction at the corneal vertex, S_{CV}=S/(1-0.014S), K is the mean corneal power, n=1.336, and c_{BR}=2.891±0.778 mm.

Analysis

Statistical analysis was performed using the SPSS statistical software package (Version 22.0, IBM Corp., US). Descriptive statistics were calculated. Continuous data are expressed as the mean ± standard deviation (mean±SD). As biometric data for the right and left eyes were highly correlated (Pearson correlation coefficient, r=0.91), analyses were performed using data for the right eye only. The AL, CRC, AL/CRC ratio, and SER were analysed as continuous variables, and the Kolmogorov–Smirnov test was performed to verify the normality of these variables. To show the correlations between SER and AL, CRC, and the AL/CRC ratio, Spearman's correlation analyses were performed. The relationship between the AL/CRC ratio and other variables was assessed using Spearman's correlation. SER, AL, CRC, AL/CRC ratio, ACD and LP were compared between groups using independent sample one-way ANOVA. Separate linear regression models were constructed with SER as the dependent variable and AL/CRC ratio, AL, and CRC as the main covariate, respectively. The sensitivity, specificity, Youden index, true positive rate, and false positive rate of the AL/CRC ratio and AL for hyperopia reserve and myopia assessment were analysed. The effectiveness of the AL/CR ratio, CRC and AL for hyperopia reserve and myopia assessment was evaluated using receiver operating characteristic (ROC) curves and the area under the curve (AUC) of the ROC curves. All p values were two sided, and a p value of < 0.05 was indicated statistical significance.

RESULTS

Demographic characteristics

A total of 1024 children (537 boys and 487 girls) aged 4-6 years (mean 5.77±0.47 years) were included in this study. The average ages of the boys and girls were 5.79

For peer review only

and 5.76 years, respectively. There was no significant difference in sex distribution ($\chi^2 = 2.441$, p=0.118). The mean SER was 0.90±1.11 D, mean AL was 22.59±0.76 mm, mean CRC was 7.74±0.24 mm and mean AL/CR ratio was 2.92±0.08. The detailed demographic characteristics of the subjects are shown in **Supplemental Table 1**.

The histograms of SER, AL, CRC and AL/CRC ratio are shown in **Supplemental Figure 1**. The Kolmogorov–Smirnov test indicated that the frequency distribution was nonnormal for SER, AL, CRC and the AL/CRC ratio (all p<0.05). The overall prevalence rates of myopia and high myopia were 7.3% and 0.1%, respectively. The prevalence rates of myopia in subjects aged 4, 5, and 6 years were 4.2% (1/24), 5.4% (10/186), and 7.9% (64/814), respectively.

Table 1 shows the distribution of ocular biometry and the AL/CRC ratio according to the categories of refractive status of subjects. Axial length was significantly longer (p<0.001) in myopes (23.74 ± 0.65 mm) than in emmetropes (22.86 ± 0.63 mm) and hyperopes (22.42 ± 0.67 mm) (p<0.001). The AL/CRC ratio was also significantly higher in myopes (3.08±0.07) than in emmetropes (2.95±0.05) and hyperopes (2.90±0.06) (p<0.001), while CRC and ACD tended to stabilize in different refractive statuses. Emmetropes have the largest LP, while myopes have the smallest LP.

Table 1. Values of ocular parameters and correlations between AL, CRC, AL/CRC ratio and SER according to refractive status

Parameters	Hyperopes (n=772)	Emmetropes (n=174)	Myopes (n=78)	p value
SER	+1.32±0.73	+0.18±0.30	-1.70±1.05	<0.001
AL	22.42±0.67	22.86±0.63	23.74±0.65	<0.001
CRC	7.74±0.24	7.76±0.26	7.72±0.21	0.678
AL/CRC	2.90±0.06	2.95±0.05	3.08±0.07	<0.001

For peer review only

ACD	3.35±0.27	3.46±0.23	3.30±0.26	0.140
LP	24.18±1.27	24.31±1.32	22.62±1.28	<0.001
Spearman's correlation coefficients				
SER and AL	-0.41*	-0.08	-0.37*	
SER and CRC	-0.06	0.10	-0.15	
SER and AL/CRC	-0.54*	-0.33*	-0.67*	

SER, spherical equivalent refractive error; AL, axial length; CRC, corneal radius of curvature; AL/CRC ratio, axial length to corneal radius of curvature ratio; LP, lens power; ACD, anterior chamber depth; D, diopter. Significant differences are masked by asterisks.

Correlation analysis between AL, CRC, AL/CRC ratio and SER

Figure 1 shows the scatter plots between AL, CRC, AL/CRC ratio and SER. The SER showed a better correlation with the AL/CRC ratio (Spearman's correlation coefficient, $\rho=-0.66$, $p<0.001$) than either AL ($\rho=-0.52$, $p<0.001$) or CRC ($\rho=-0.03$, $p=0.33$) (**Table 2**). The Spearman's correlation coefficient between SER and the AL/CRC ratio was the highest in myopes ($\rho=-0.67$, $p<0.001$), followed by hyperopes ($\rho=-0.54$, $p<0.001$) and emmetropes ($\rho=-0.33$, $p<0.001$). There was a significant correlation between SER and AL in hyperopes ($\rho=-0.41$, $p<0.001$) and myopes ($\rho=-0.37$, $p<0.001$), but not in emmetropes ($\rho=-0.08$, $p=0.28$). There was no statistical significance for the correlation between SER and CRC in all refractive groups (all $p>0.05$). Compared to either AL or CRC, the AL/CRC ratio showed better correlation with SER in all refractive groups (**Table 1**).

Regression analysis between AL, CRC, AL/CRC ratio and SER

Univariate and multiple linear regression analyses were performed in this study (**Table 2**). For SER variance, AL explained 34.5%, whereas the AL/CRC ratio explained 64.5% of the data after adjusting for age and sex. Multiple linear regression adjusted for age and sex showed that a 0.1 increase in the AL/CRC ratio was associated

For peer review only

with a -1.19 D change in SER and a 1 mm increase in AL was associated with a -0.87 D change in SER.

Table 2. Association between AL/CRC ratio, AL, and CRC with SER.

Variables	Correlation coefficient	P value	Univariate OR (95% CI)	P value	Gender and age-adjusted Multivariate OR (95% CI)				
					Unstandardised coefficient	95% CI	Standardised coefficient	P value	R^2
AL	-0.52	<0.001	-0.86 (-0.94 to -0.79)	<0.001	-0.869	-0.942 to -0.795	-0.590	<0.001	0.345
CRC	-0.03	0.33	0.15 (-0.14 to 0.43)	0.31	0.148	-0.135 to 0.430	0.032	0.306	0.002
AL/CRC	-0.66	<0.001	-11.73 (-12.27 to -11.19)	<0.001	-11.876	-12.418 to -11.334	-0.809	<0.001	0.645

SER, spherical equivalent refractive error; AL, axial length; CRC, corneal radius of curvature; AL/CRC ratio, axial length to corneal radius of curvature ratio. OR, odds ratio.

Effectiveness of AL, CRC and the AL/CR ratio for hyperopia reserve assessment and myopia assessment

Taking cycloplegic refraction SER ≤ +0.50 D as the standard for diagnosis of the lack of hyperopia reserve [37], the effectiveness of AL, CRC and AL/CR ratio for myopia assessment were analysed (**Figure 2A**). The ROC curves were drawn using AL, CRC and the AL/CR ratio as the index for hyperopia reserve assessment, and the AUCs of the ROC curves were 0.777 (95% confidence interval, CI: 0.741-0.812), 0.494(95% CI: 0.450-0.539) and 0.861 (95% CI: 0.829-0.892), respectively. If AL was used for hyperopia reserve assessment, the optimal cut-off point of the ROC curve was ≥22.615 mm, with a specificity, sensitivity, Youden index, true positive rate, and false positive rate of 0.785, 0.604, 0.389, 0.604 and 0.215, respectively. If the AL/CR ratio was used for myopia assessment, the optimal cut-off point of the ROC curve was ≥2.955, with a specificity, sensitivity, Youden index, true positive rate, and false positive rate of 0.732, 0.863, 0.595, 0.863, and 0.268, respectively (**Table 3**). Error low hyperopia reserve assessment using optimal cut-off value of AL/CRC ratio was 22.22% (56/252) (**Supplemental Table 2**).

Table 3. The sensitivity, specificity, and Youden index of AL and the AL/CRC ratio for hyperopia reserve assessment and myopia assessment.

	SER	Criterion	Cut-off value	Sensitivity	Specificity	Youden index

For peer review only

Hyperopia reserve assessment	≤ +0.50 D	AL (mm)	22.615	0.785	0.604	0.389
		AL/CRC ratio	2.955	0.732	0.863	0.595
	≤ +1.00 D	AL (mm)	22.515	0.677	0.693	0.370
		AL/CRC ratio	2.925	0.670	0.774	0.444
	≤ +1.50 D	AL (mm)	22.495	0.625	0.778	0.403
		AL/CRC ratio	2.895	0.736	0.736	0.472
Myopia assessment	≤ -0.50 D	AL (mm)	23.235	0.800	0.862	0.662
		AL/CRC ratio	2.975	0.894	0.883	0.777

AL, axial length; AL/CRC ratio, axial length to corneal radius of curvature ratio.

Taking cycloplegic refraction SER ≤ −0.50 D as the gold standard for diagnosis of myopia [19], the effectiveness of AL, CRC and AL/CR ratio for myopia assessment were analysed (**Figure 2B**). The ROC curves were drawn using AL, CRC and the AL/CR ratio as the index for myopia assessment, and the AUCs of the ROC curves were 0.898 (95% CI: 0.864-0.932), 0.493(95% CI: 0.434-0.553) and 0.954 (95% CI: 0.925-0.982), respectively. If AL was used for myopia assessment, the optimal cut-off point of the ROC curve was ≥23.235 mm, with a specificity, sensitivity, Youden index, true positive rate, and false positive rate of 0.862, 0.800, 0.662, 0.800 and 0.138, respectively. If the AL/CR ratio was used for myopia assessment, the optimal cut-off point of the ROC curve was ≥2.975, with a specificity, sensitivity, Youden index, true positive rate, and false positive rate of 0.883, 0.894, 0.777, 0.894, and 0.117, respectively (**Table 3**). Error myopia assessment using optimal cut-off value of AL/CRC ratio was 7.06% (6/85) (**Supplemental Table 2**).

DISCUSSION

This study found that AL/CRC ratio was correlated with SER in hyperopes, emmetropes and myopes, while AL showed correlation with SER in hyperopes and myopes, but not in emmetropes. The SER showed a better correlation with the AL/CRC ratio than either AL or CRC alone in different refractive statuses, especially in myopes, among 4- to 6-year-old children. The effect of the AL/CR ratio for low hyperopia reserve and myopia assessment was higher than that of either AL or CRC alone. These findings indicate that when cycloplegic refraction is unavailable, AL/CRC ratio can be used as an alternative indicator for monitoring low hyperopia reserve and myopia among preschoolers.

Myopia is a common disorder of the eyes and has become a global public health concern [38,39]. During the past several decades, the prevalence of myopia in

For peer review only

children has increased rapidly, and the age at onset of myopia has decreased. Chinese children tend to have a higher prevalence of myopia than children in other countries and of other ethnicities. The prevalence of myopia exceeds 60% among 12 years of age in China after primary school, reaches nearly 80% at 16 years of age after junior high school, and surpasses 90% in university students [1,4,13]. Notably, the incidence of myopia increased among preschool children in recent years. The prevalence of myopia was 17.6% in children aged 6 years in Hong Kong [40]. A study in Shanghai of 7166 Chinese children aged 4 to 6 years reported that the prevalence of myopia ranged from 5.7% to 7.1% [41]. In this study, the prevalence of myopia in preschoolers increased from 4.2% at 4 years of age to 7.9% at 6 years of age. Although myopia tends to stabilize after adulthood, children with a younger age at myopia onset are prone to develop high myopia and have a high risk of suffering from vision-threatening complications. In view of this condition, it is urgent to carry out early vision screening and refractive error assessment among preschool children. Ocular biometrics have been used to estimate the progression of refractive error [29,34]. The AL/CRC ratio summarizes the overall relationship between AL and CRC and minimizes variability in AL and CRC, thus, it has been considered as a more robust index to evaluate the refractive status of the eye [17]. Moreover, the AL/CRC ratio also correlates with refractive error more strongly than AL or CRC alone [28,31]. Therefore, the AL/CRC ratio could be useful in determining refractive error and monitoring myopia progression.

Changes in AL and CRC are important ocular biometrics affecting refractive status [34]. Previous studies have indicated that CRC may be actively modulated to regulate refractive development, and the onset of myopia is due to a failure of corneal compensation for axial elongation [11,33]. In this study, we found that there was no correlation between CRC and refractive error, while AL, or more strikingly, the AL/CRC ratio had a significant correlation with refractive error. This finding suggests that AL has a larger effect on refractive error than CRC. Grosvenor et al. was the first to report the correlation between the AL/CRC ratio and refractive error and studied the role of the AL/CRC ratio in determining the refractive state of the eye [42]. They demonstrated that the AL/CRC ratio was the most important biometric factor in myopia. Some researchers further confirmed Grosvenor's findings [29,43,44]. The distribution of AL/CRC ratio has been reported in different countries and age populations. A summary of AL/CRC ratio in other studies is shown in **Supplemental Table 3**. The average values reported for the AL/CRC ratio range from 2.81 to 3.13. On the one hand, differences in age, race, included subjects, measurement methods and techniques may account for the disagreement in the various studies. On the other hand, we can find that the older age, the greater value of AL/CRC ratio. **Supplemental Figure 2** also confirms this result. The AL/CRC ratio has been proposed as a calculated biometric parameter that correlates better with SER than AL, and the AL/CRC ratio could explain the total variance in SER better than AL alone [31]. This is because the AL/CRC ratio provides a rough measure of the degree of matching between AL and corneal power. In this study, we also found a better correlation between the AL/CRC ratio and SER in children aged 4-6 years compared to that of AL or CRC alone, especially in children with myopia. It is possibly suggested that the AL/CRC ratio is a more useful marker of progress towards myopic refractions than absolute ocular biometric values such as AL or CRC. Moreover, the value of the AL/CRC ratio was negatively correlated with SER. As demonstrated in this study, the AL/CRC ratio was significantly higher in myopes than in emmetropes and hyperopes, which was consistent with studies performed by Foo et al. and Guo et al. [28,31]. This finding indicates that the greater the myopic

For peer review only

refraction, the larger the AL/CRC ratio.

A previous study reported that a high AL/CRC ratio of no less than 3.0 has been considered an indicator for the onset of myopia [42]. High axial length to corneal radius ratio is a risk factor in the development of myopia. When the AL/CRC ratio was higher than 3.0, it was considered that other ocular parameters were not able to compensate for the change in refraction caused by the elongation of AL, thereby inducing myopia. As shown in this study, the area under the ROC of the AL/CR ratio for myopia assessment was 0.954, and the optimal cut-off value is ≥2.975, which was smaller than that in the study performed by Mu et al. [29]. This result indicates that for children aged 4 to 6 years, the risk of myopia is likely to occur when the AL/CRC ratio is greater than 2.975. We also conducted an evaluation of the effectiveness of the AL/CRC ratio, CRC and AL for hyperopia reserve assessment in this population. Previous study has reported that hyperopia reserve is a potential indicator of myopia [19]. There is a progressive shift in mean refraction from roughly + 2.00 D at 3 months of age to approximately + 1.00 D at 6.5 years of age [45]. Then emmetropization continues at a slower rate after 6 years of age. Zadnik et al. revealed that children in the United States in grade 1 with less than +0.75 D of hyperopia are at increased risk of myopia onset [18]. Yue et al. also reported that the cut point for myopia for grade 1 students was +0.31 D in the Chinese population [19]. Li et al.'s [41] findings showed that the 5-year cumulative incidence of myopia was 4.6%, 26.3%, 52.3%, 78.6%, 92.6% and 94.3%, respectively, corresponding to students with a baseline hyperopia reserve of >+2.00 D, +1.50 D to +2.00 D, +1.00 D to +1.50 D, +0.50 D to +1.00 D, 0.00 D to +0.50 D and -0.50 D to 0.00 D, respectively. The lower the hyperopia reserve in young children, the higher the incidence of myopia onset. In our study, taking cycloplegic refraction SER ≤ +0.50 D as the standard for diagnosis of the lack of hyperopia reserve, the area under the ROC curve of the AL/CR ratio for the lack of hyperopia reserve assessment was greater than that of AL and CRC and the optimal cut-off value of the AL/CRC ratio was ≥2.955. Thus, clinicians and parents could monitor the hyperopia reserve of young children by regularly measuring the axial length and corneal curvature when cycloplegic refraction is unavailable. When the AL/CRC ratio is greater than 2.955 among preschool children, clinicians and parents should pay attention to the children with less hyperopia reserve and begin to take measures to prevent myopia formation.

In the present study, AL alone contributed 34.5% of the variance in SER, whereas the AL/CR ratio accounted for 64.5%. Guo et al. study [31] found that AL alone contributed 18.6% of the variance in SER, whereas the AL/CR ratio accounted for 39.8% in the study population of preschoolers aged 3-6 years old. He et al. [30] reported that AL/CR ratio and AL explained 66.4% and 43.1% of the variance in SER, respectively, in a group of Chinese children 6 to 12 years of age. This suggested that the AL/CRC ratio is more closely related to refractive error than AL or CRC alone. Moreover, we found a linear increase in AL/CRC ratio from hyperopia towards myopia. The corresponding increase in SER of -1.1 9D with every 0.1 increase in the AL/CRC ratio in this study is larger than -0.74 D reported by researchers in Singapore [28] and -0.89D reported among the Nigerian population [33], but slightly lower than -1.21 D reported among northern Iranian subjects [17]. The above findings revealed that in addition to AL and CRC, there are other potential factors that influence SER results. Thus, the AL/CRC ratio cannot completely replace cycloplegic refraction in determining refraction in children, avoiding misunderstandings for parents and non-professionals. However, cycloplegic refraction is easily influenced by the cooperation and compliance of the examinees, especially in younger children, which is not feasible for large-scale myopia assessment and long-term

For peer review only

refractive development follow-up. For this reason, the AL/CRC ratio could be used as an alternative indicator for refraction estimation, and it may be more accurate than AL. In addition, measurement of AL and CRC and determination of the AL/CRC ratio are relatively easy and can be conducted by a technician using noncontact, objective, and automated instruments. The application of the AL/CRC ratio reference as an indicator of refractive errors has the effect of simplifying the examination procedure and reducing unnecessary cycloplegia, especially when cycloplegic refraction is difficult to perform in 4- to 6-year-old children.

There were some limitations in this study. First, because this study is cross-sectional and does not pinpoint the temporal correlations between refractive error and the AL/CRC ratio, it is impossible to rule out the possibility of observational and inclusionary biases due to the retrospective study design. Admittedly, longitudinal studies would be needed to conduct to evaluate intraindividual discrepancy. In addition, the data from Beijing may not be representative for the rural areas and other regions in China. Last, the sample size of the 4-year-old participants (N=24) was relatively small. Although our findings are applicable to this population as well, there is potential for discrepancy. We will further increase the sample size of this population to increase the accuracy and reliability of the results of this study.

In conclusion, our study found that SER showed a better correlation with the AL/CRC ratio than either AL or CRC alone in different refractive statuses, especially in myopic children, among 4- to 6-year-old Chinese children. The AL/CRC ratio could explain the total variance in refractive error better than AL alone. The effect of the AL/CR ratio for assessing the lack of hyperopia reserve and for the myopia assessment was higher than that of AL and CRC. These findings indicate that when cycloplegic refraction is unavailable, the AL/CRC ratio could be used as an alternative indicator for identifying low hyperopia reserve and myopia among preschoolers, helping clinicians and parents screen children with low hyperopia reserve before primary school in a timely manner. In the future, longitudinal cohort designs will be conducted for optimal study of the effect of the AL/CRC ratio on hyperopia reserve and myopia assessment in preschool children.

Declarations

Authors' contributions

TT developed the concept, collected data, performed the experiments and data analysis, wrote the main manuscript text and prepared Figures 1-2, Tables 1-3; HZ collected data and prepared Figure 2; DL and XL collected data; KW and YL developed the concept and designed the experiments; MZ developed the study conception. TT wrote the first draft of the manuscript. TT, KW and YL made critical revisions. All authors reviewed and approved the final draft. TT is the guarantor and responsible for the overall content.

For peer review only

Funding

This work was supported by: Capital's Funds for Health Improvement and Research (No. 2022-1G-4083), National Natural Science Foundation of China (Grant Nos. 82171092), and the National Key R&D Program of China (No.2020YFC2008200 and No.2021YFC2702100).

Competing interests

The authors declare that they have no conflicts of interest.

Patient consent for publication

Not applicable.

Ethics approval

This study was approved by the institutional research ethics committee of Peking University People's Hospital (2021PHB322-001). All examinations and management were performed in accordance with the Declaration of Helsinki, and written informed consent was obtained from the parents.

Availability of data and materials

All data generated or analysed during this study are included in this published article.

Acknowledgements

None.

Corresponding author

Correspondence to Kai Wang and Yan Li

Data Availability Statement

All data generated or analysed during this study are included in this article and its online supplementary material. Further inquiries can be directed to the corresponding author.

For peer review only

References

1. Ma Y, Qu X, Zhu X, et al. Age-Specific Prevalence of Visual Impairment and Refractive Error in Children Aged 3-10 Years in Shanghai, China. Invest Ophthalmol Vis Sci. 2016;57(14):6188-6196.
2. Koh V, Yang A, Saw SM, et al. Differences in Prevalence of Refractive Errors in Young Asian Males in Singapore between 1996–1997 and 2009–2010. Ophthalmic Epidemiology. 2014;21(4):247-255.
3. Jung SK, Lee JH, Kakizaki H, Jee D. Prevalence of myopia and its association with body stature and educational level in 19-year-old male conscripts in seoul, South Korea. Invest Ophthalmol Vis Sci. 2012 Aug 15;53(9):5579-83.
4. Holden BA, Fricke TR, Wilson DA, et al. Global Prevalence of Myopia and High Myopia and Temporal Trends from 2000 through 2050. Ophthalmology. 2016;123(5):1036-1042.
5. Lyu Y, Zhang H, Gong Y, et al. Prevalence of and factors associated with myopia in primary school students in the Chaoyang District of Beijing, China. Jpn J Ophthalmol. 2015;59(6):421-429.
6. Choy BNK, You Q, Zhu MM, Lai JSM, Ng ALK, Wong IYH. Prevalence and associations of myopia in Hong Kong primary school students. Jpn J Ophthalmol. 2020;64(4):437-449.
7. Matamoros E, Ingrand P, Pelen F, et al. Prevalence of Myopia in France: A Cross-Sectional Analysis. Medicine (Baltimore). 2015;94(45):e1976.
8. Lee JY, Sung KR, Han S, Na JH. Effect of myopia on the progression of primary open-angle glaucoma. Invest Ophthalmol Vis Sci. 2015;56(3):1775-1781.
9. Ohno-Matsui K, Wu PC, Yamashiro K, et al. IMI Pathologic Myopia. Invest Ophthalmol Vis Sci. 2021;62(5):5.
10. Haarman AEG, Enthoven CA, Tideman JWL, Tedja MS, Verhoeven VJM, Klaver CCW. The Complications of Myopia: A Review and Meta-Analysis. Invest Ophthalmol Vis Sci. 2020;61(4):49.
11. Mutti DO, Mitchell GL, Jones LA, et al. Axial growth and changes in lenticular and corneal power during emmetropization in infants. Invest Ophthalmol Vis Sci. 2005;46(9):3074-3080.
12. Flitcroft DI. Emmetropisation and the aetiology of refractive errors. Eye (Lond). 2014;28(2):169-179.
13. Song Y, Zhu S, Yang B, et al. Accommodation and binocular vision changes after wearing orthokeratology lens in 8- to 14-year-old myopic children. Graefes Arch Clin Exp Ophthalmol. 2021;259(7):2035-2045.
14. Rozema J, Dankert S, Iribarren R. Emmetropization and nonmyopic eye growth. Surv Ophthalmol. 2023; 68(4):759-783.
15. Ojaimi E, Rose KA, Morgan IG, et al. Distribution of ocular biometric parameters and refraction in a population-based study of Australian children. Invest

For peer review only

Ophthalmol Vis Sci. 2005;46(8):2748-2754.

16. Chamberlain P, Lazon de la Jara P, Arumugam B, Bullimore MA. Axial length targets for myopia control. Ophthalmic Physiol Opt. 2021 May;41(3):523-531.

17. Hashemi H, Khabazkhoob M, Miraftab M, Emamian MH, Shariati M, Abdolahi-Nia T, et al. Axial length to corneal radius of curvature ratio and refractive errors. J Ophthalmic Vis Res. 2013 Jul;8(3):220-6.

18. Zadnik K, Sinnott LT, Cotter SA, et al. Prediction of Juvenile-Onset Myopia. JAMA Ophthalmol. 2015;133(6):683-689.

19. Yue Y, Liu X, Yi S, Liu B, Yi H, Li H. High prevalence of myopia and low hyperopia reserve in 4411 Chinese primary school students and associated risk factors. BMC Ophthalmol. 2022;22(1):212.

20. Hyman L, Gwiazda J, Hussein M, Norton TT, Wang Y, Marsh-Tootle W, et al. Relationship of age, sex, and ethnicity with myopia progression and axial elongation in the correction of myopia evaluation trial. Arch Ophthalmol. 2005 Jul;123(7):977-87.

21. Morgan IG, Iribarren R, Fotouhi A, Grzybowski A. Cycloplegic refraction is the gold standard for epidemiological studies. Acta Ophthalmol. 2015;93(6):581-585.

22. Li T, Zhou X, Zhu J, Tang X, Gu X. Effect of cycloplegia on the measurement of refractive error in Chinese children. Clin Exp Optom. 2019;102(2):160-165.

23. Hu YY, Wu JF, Lu TL, et al. Effect of cycloplegia on the refractive status of children: the Shandong children eye study. PLoS One. 2015;10(2):e0117482.

24. Chia A, Chua WH, Cheung YB, et al. Atropine for the treatment of childhood myopia: safety and efficacy of 0.5%, 0.1%, and 0.01% doses (Atropine for the Treatment of Myopia 2). Ophthalmology. 2012;119(2):347-354.

25. Gong Q, Janowski M, Luo M, et al. Efficacy and Adverse Effects of Atropine in Childhood Myopia: A Meta-analysis. JAMA Ophthalmol. 2017;135(6):624-630.

26. Wei S, Li SM, An W, et al. Safety and Efficacy of Low-Dose Atropine Eyedrops for the Treatment of Myopia Progression in Chinese Children: A Randomized Clinical Trial. JAMA Ophthalmol. 2020;138(11):1178-1184.

27. Scheiman M, Gwiazda J, Zhang Q, et al. Longitudinal changes in corneal curvature and its relationship to axial length in the Correction of Myopia Evaluation Trial (COMET) cohort. J Optom. 2016;9(1):13-21.

28. Foo VH, Verkicharla PK, Ikram MK, Chua SY, Cai S, Tan CS, et al. Axial Length/Corneal Radius of Curvature Ratio and Myopia in 3-Year-Old Children. Transl Vis Sci Technol. 2016;5(1):5.

29. Mu J, Zeng D, Fan J, et al. The accuracy of the axial length and axial length/corneal radius ratio for myopia assessment among Chinese children. Front Pediatr. 2022;10:859944.

30. He X, Zou H, Lu L, et al. Axial length/corneal radius ratio: association with refractive state and role on myopia detection combined with visual acuity in

For peer review only

For peer review only - http://bmjopen.bmj.com/site/about/guidelines.xhtml

Chinese schoolchildren. PLoS One. 2015;10(2):e0111766.

31. Guo X, Fu M, Ding X, Morgan IG, Zeng Y, He M. Significant Axial Elongation with Minimal Change in Refraction in 3- to 6-Year-Old Chinese Preschoolers: The Shenzhen Kindergarten Eye Study. Ophthalmology. 2017;124(12):1826-1838.

32. Jong M, Sankaridurg P, Naduvilath TJ, Li W, He M. The Relationship between Progression in Axial Length/Corneal Radius of Curvature Ratio and Spherical Equivalent Refractive Error in Myopia. Optom Vis Sci. 2018;95(10):921-929.

33. Badmus SA, Ajaiyeoba AI, Adegbehingbe BO, Onakpoya OH, Adeoye AO. Axial length/corneal radius of curvature ratio and refractive status in an adult Nigerian population. Niger J Clin Pract. 2017;20(10):1328-1334.

34. Iyamu E, Iyamu J, Obiakor CI. The role of axial length-corneal radius of curvature ratio in refractive state categorization in a nigerian population. ISRN Ophthalmol. 2011;2011:138941.

35. Warrier S, Wu HM, Newland HS, et al. Ocular biometry and determinants of refractive error in rural Myanmar: the Meiktila Eye Study. Br J Ophthalmol. 2008;92(12):1591-1594.

36. Rozema JJ, Atchison DA, Tassignon MJ. Comparing methods to estimate the human lens power. Invest Ophthalmol Vis Sci. 2011;52(11):7937-7942.

37. Li SM, Wei S, Atchison DA, et al. Annual Incidences and Progressions of Myopia and High Myopia in Chinese Schoolchildren Based on a 5-Year Cohort Study. Invest Ophthalmol Vis Sci. 2022;63(1):8.

38. Foster PJ, Jiang Y. Epidemiology of myopia. Eye (Lond). 2014;28(2):202-208.

39. Dolgin E. The myopia boom. Nature. 2015;519(7543):276-8.

40. Lam CS, Lam CH, Cheng SC, Chan LY. Prevalence of myopia among Hong Kong Chinese schoolchildren: changes over two decades. Ophthalmic Physiol Opt. 2012;32(1):17-24.

41. Li T, Zhou X, Chen X, Qi H, Gao Q. Refractive Error in Chinese Preschool Children: The Shanghai Study. Eye Contact Lens. 2019;45(3):182-187.

42. Grosvenor T. High axial length/corneal radius ratio as a risk factor in the development of myopia. Am J Optom Physiol Opt. 1988 Sep;65(9):689-96.

43. Matsumura S, Dannoue K, Kawakami M, et al. Prevalence of Myopia and Its Associated Factors Among Japanese Preschool Children. Front Public Health. 2022;10:901480.

44. Liu S, Chen J, Wang J, Zhu Z, Zhang J, Zhang B, Yang J, Du L, Zhu J, Zou H, He X, Xu X. Cutoff values of axial length/corneal radius ratio for determining myopia vary with age among 3-18 years old children and adolescents. Graefes Arch Clin Exp Ophthalmol. 2023.

45. Mutti DO, Sinnott LT, Lynn Mitchell G, et al. Ocular Component Development during Infancy and Early Childhood. Optom Vis Sci. 2018;95(11):976-985.

For peer review only

Legends:

Figure 1. Correlation analysis between AL, CRC, AL/CRC ratio and SER. SER, spherical equivalent refractive error; AL, axial length; CRC, corneal radius of curvature; AL/CRC ratio, axial length to corneal radius of curvature ratio, D, diopter.

Figure 2. Comparison of the effectiveness of AL, CRC and the AL/CR ratio for hyperopia reserve assessment (A) and myopia assessment (B). AL, axial length; CRC, corneal radius of curvature; AL/CRC ratio, axial length to corneal radius of curvature ratio; AUC, area under curve.

For peer review only

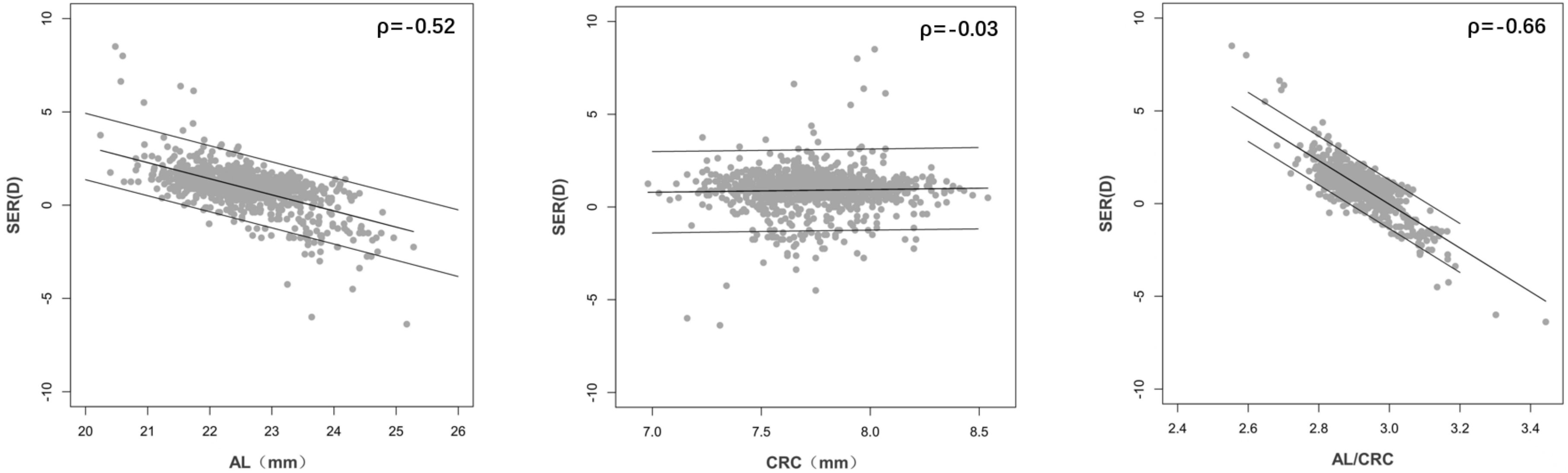

Figure 1. Correlation analysis between AL, CRC, AL/CRC ratio and SER. SER, spherical equivalent refractive error; AL, axial length; CRC, corneal radius of curvature; AL/CRC ratio, axial length to corneal radius of curvature ratio, D, diopter.

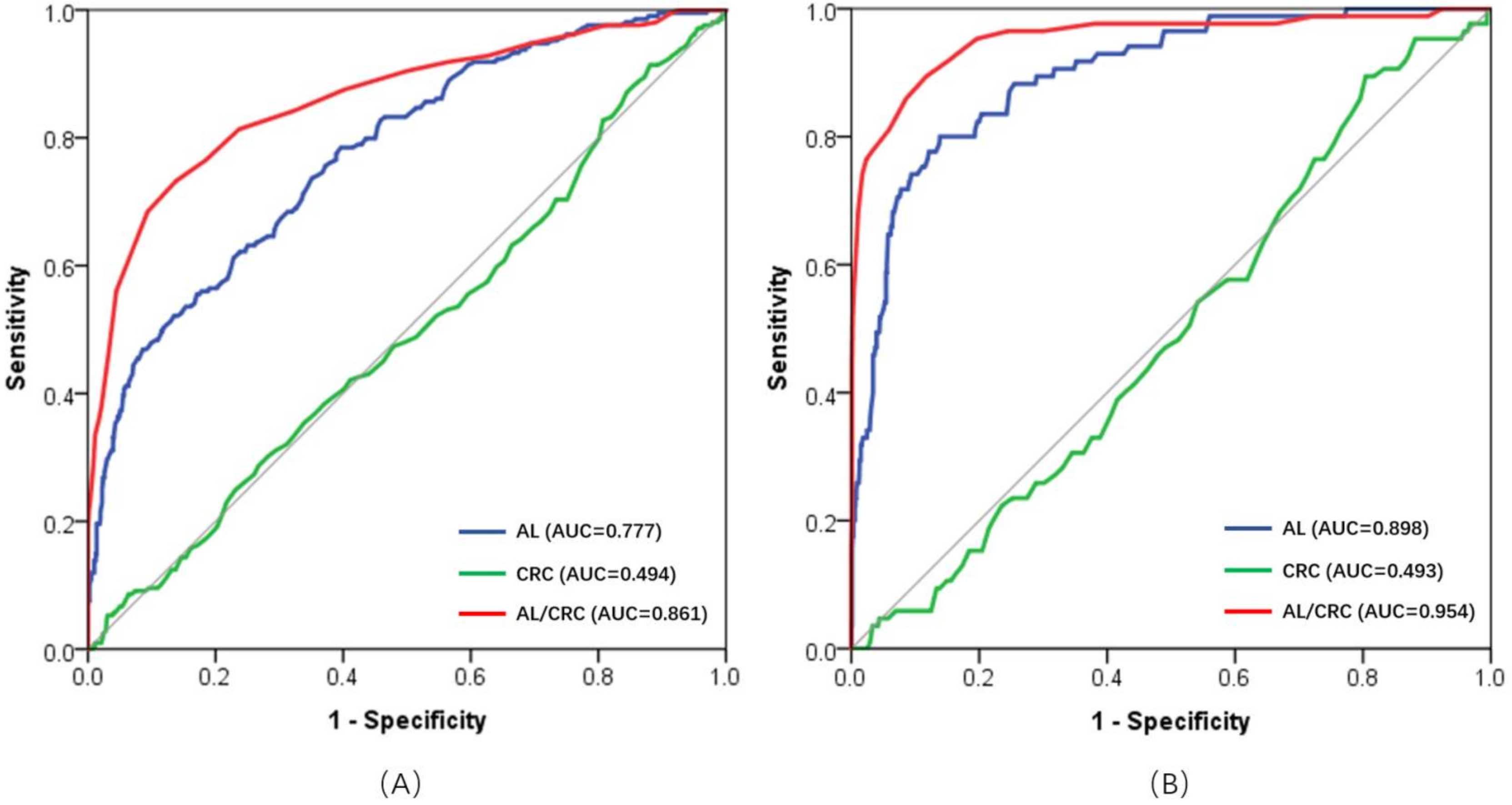

Figure 2. Comparison of the effectiveness of AL, CRC and the AL/CR ratio for hyperopia reserve assessment (A) and myopia assessment (B). AL, axial length; CRC, corneal radius of curvature; AL/CRC ratio, axial length to corneal radius of curvature ratio; AUC, area under curve.

Table 2. Association between AL/CRC ratio, AL, and CRC with SER.

Variables	Correlation coefficient	P value	Univariate OR (95% CI)	P value	Gender and age-adjusted Multivariate OR (95% CI)				
					Unstandardised coefficient	95% CI	Standardised coefficient	P value	R^2
AL	-0.52	<0.001	-0.86 (-0.94 to -0.79)	<0.001	-0.869	-0.942 to -0.795	-0.590	<0.001	0.345
CRC	-0.03	0.33	0.15 (-0.14 to 0.43)	0.31	0.148	-0.135 to 0.430	0.032	0.306	0.002
AL/CRC	-0.66	<0.001	-11.73 (-12.27 to -11.19)	<0.001	-11.876	-12.418 to -11.334	-0.809	<0.001	0.645

SER, spherical equivalent refractive error; AL, axial length; CRC, corneal radius of curvature; AL/CRC ratio, axial length to corneal radius of curvature ratio. OR, odds ratio.

For peer review only

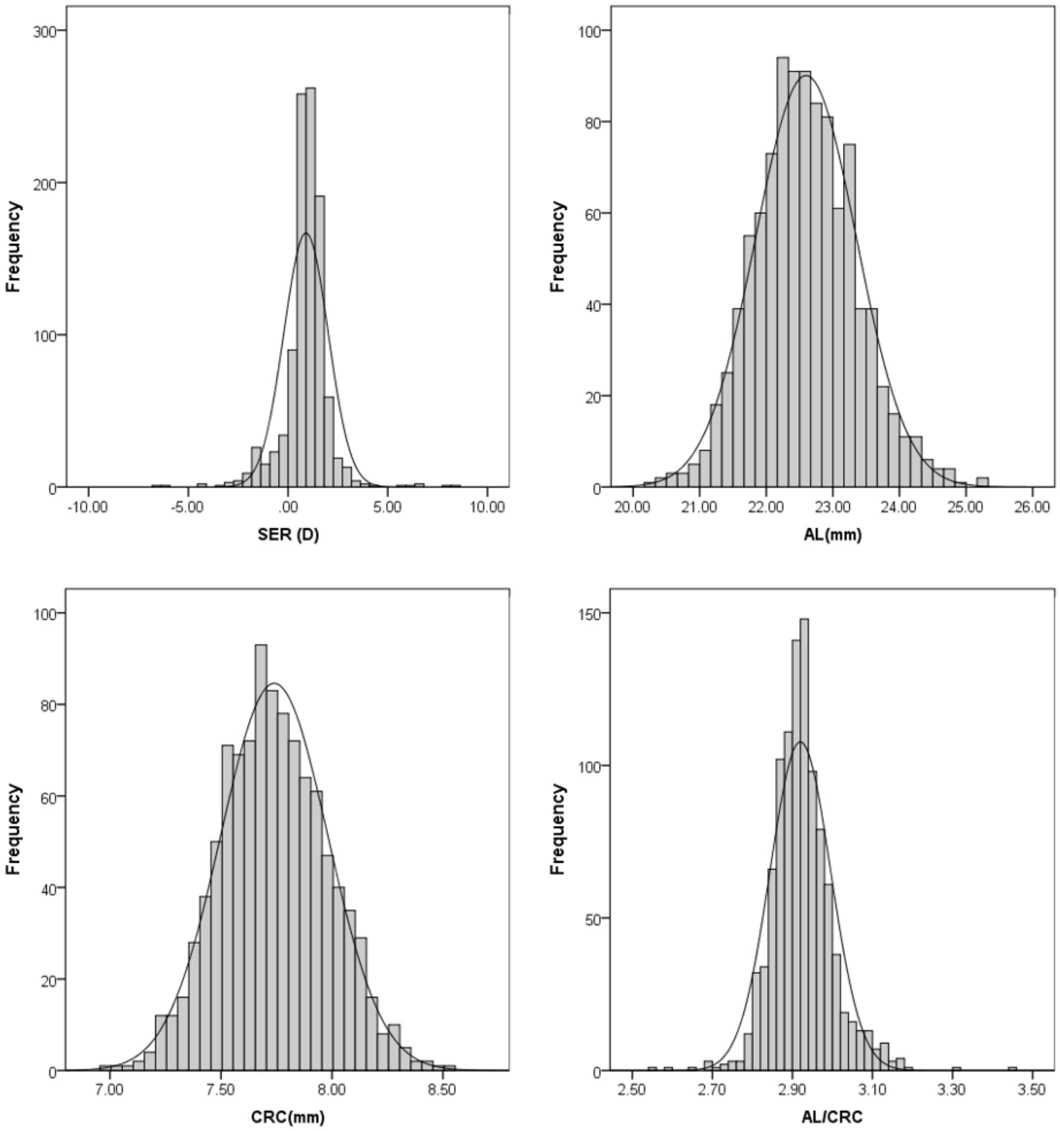

Supplemental Figure 1. Histograms showing distributions of SER, AL, CRC and AL/CRC ratio. SER, spherical equivalent refractive error; AL, axial length; CRC, corneal radius of curvature; AL/CRC ratio, axial length to corneal radius of curvature ratio; D, diopter.

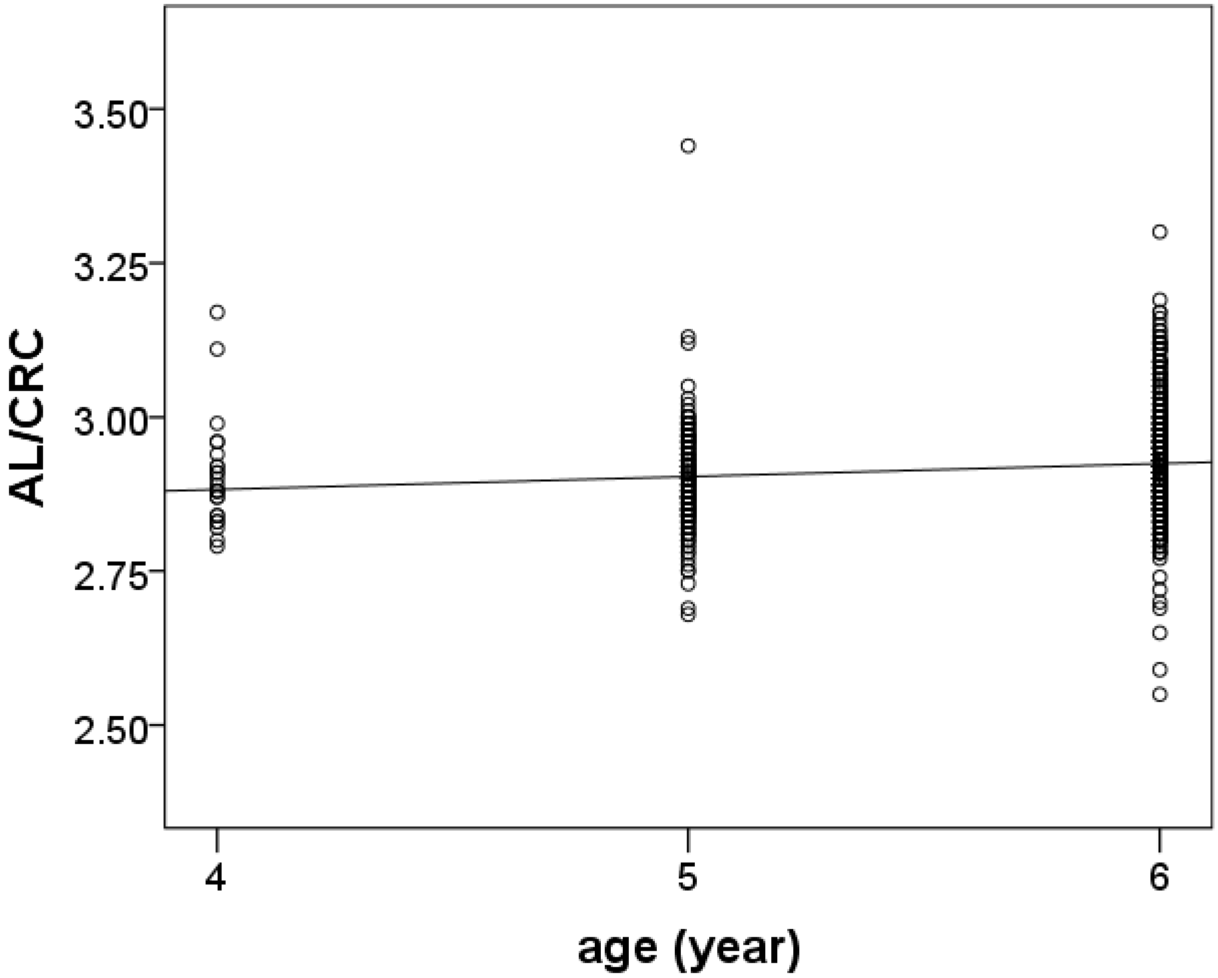

Supplemental Figure 2. Scatterplot of AL/CRC ratio and age. AL/CRC, axial length to corneal radius of curvature ratio.

Supplemental Table 1. Demographic and biometric data of included participants

Parameters	Total, N=1024		Boys, N=537	Girls, N=487	P value
	Mean±SD	Range	Mean±SD	Mean±SD	
Age (years)	5.77±0.47	4-6	5.85±0.35	5.68±0.56	<0.001
SER (D)	0.90±1.11	-6.38 to 8.50	0.90±1.09	0.90±1.13	0.922
AL (mm)	22.59±0.76	20.24 to 25.28	22.62±0.77	22.57±0.74	0.350
CRC (mm)	7.74±0.24	6.98 to 8.54	7.74±0.24	7.74±0.24	0.805
AL/CRC	2.92±0.08	2.65 to 3.17	2.92±0.08	2.92±0.08	0.141
LP (D)	24.09±1.35	19.68 to 28.68	24.02±1.34	24.16±1.35	0.117
ACD (mm)	3.37±0.27	2.09 to 5.37	3.38±0.27	3.34±0.27	0.028

SER, spherical equivalent refractive error; AL, axial length; CRC, corneal radius of curvature; AL/CRC, axial length to corneal radius of curvature ratio; LP, lens power; ACD, anterior chamber depth; D, diopter.

For peer review only

Supplemental Table 2. Effect of optimal cut-off value of AL/CRC ratio for low hyperopia reserve assessment and true myopia assessment.

	Criterion	Total Data	True assessment	Error assessment (%)
Low hyperopia reserve assessment	AL/CRC ratio ≥2.955	252	196	56 (22.22%)
True myopia assessment	AL/CRC ratio ≥2.975	85	79	6 (7.06%)

AL/CRC ratio, axial length to corneal radius of curvature ratio.

For peer review only

Supplemental Table 3. Summary of studies reporting the AL, CRC, and AL/CRC ratio.

Origin	N	Age (years)	SER (D)	AL/CRC	AL	CRC
Australia [15]	1765	5.5-8.4	+1.26	2.906	22.61	7.70
Iran [17]	4820	40-64	-	3.033	23.14	7.63
China [19]	4411	7-10	-0.13	2.97	23.27	7.82
Singapore [28]	349	3	+0.88	2.81	21.73	7.72
Nigerian [34]	70	20-39	-0.24	3.03	23.74	7.84
Myanmar [35]	1498	40-70	-1.22	2.99	22.76	7.62
Japan [43]	457	4-6	0.13	2.88	22.35	7.76
Current study	1024	4-6	+0.90	2.92	22.59	7.74

SER, spherical equivalent refractive error; AL, axial length; CRC, corneal radius of curvature; AL/CRC ratio, axial length to corneal radius of curvature ratio.

For peer review only

STROBE Statement—Checklist of items that should be included in reports of ***cross-sectional studies***

	Item No	Recommendation	Page No
**Title and abstract**	1	(*a*) Indicate the study's design with a commonly used term in the title or the abstract	P1
		(*b*) Provide in the abstract an informative and balanced summary of what was done and what was found	P4-5
**Introduction**			
Background/rationale	2	Explain the scientific background and rationale for the investigation being reported	P5-6
Objectives	3	State specific objectives, including any prespecified hypotheses	P6
**Methods**			
Study design	4	Present key elements of study design early in the paper	P6
Setting	5	Describe the setting, locations, and relevant dates, including periods of recruitment, exposure, follow-up, and data collection	P6-7
Participants	6	(*a*) Give the eligibility criteria, and the sources and methods of selection of participants	P6-7
Variables	7	Clearly define all outcomes, exposures, predictors, potential confounders, and effect modifiers. Give diagnostic criteria, if applicable	P7
Data sources/ measurement	8*	For each variable of interest, give sources of data and details of methods of assessment (measurement). Describe comparability of assessment methods if there is more than one group	*P7*
Bias	9	Describe any efforts to address potential sources of bias	P7
Study size	10	Explain how the study size was arrived at	P7
Quantitative variables	11	Explain how quantitative variables were handled in the analyses. If applicable, describe which groupings were chosen and why	P7
Statistical methods	12	(*a*) Describe all statistical methods, including those used to control for confounding	P8
		(*b*) Describe any methods used to examine subgroups and interactions	
		(*c*) Explain how missing data were addressed	P8
		(*d*) If applicable, describe analytical methods taking account of sampling strategy	
		(*e*) Describe any sensitivity analyses	
**Results**			
Participants	13*	(a) Report numbers of individuals at each stage of study—eg numbers potentially eligible, examined for eligibility, confirmed eligible, included in the study, completing follow-up, and analysed	P8
		(b) Give reasons for non-participation at each stage	
		(c) Consider use of a flow diagram	
Descriptive data	14*	(a) Give characteristics of study participants (eg demographic, clinical, social) and information on exposures and potential confounders	P8
		(b) Indicate number of participants with missing data for each variable of interest	
Outcome data	15*	Report numbers of outcome events or summary measures	P8
Main results	16	(*a*) Give unadjusted estimates and, if applicable, confounder-adjusted estimates and their precision (eg, 95% confidence interval). Make clear which confounders were adjusted for and why they were included	P8-14

For peer review only

		(*b*) Report category boundaries when continuous variables were categorized	
		(*c*) If relevant, consider translating estimates of relative risk into absolute risk for a meaningful time period	
Other analyses	17	Report other analyses done—eg analyses of subgroups and interactions, and sensitivity analyses	
**Discussion**			
Key results	18	Summarise key results with reference to study objectives	P15-17
Limitations	19	Discuss limitations of the study, taking into account sources of potential bias or imprecision. Discuss both direction and magnitude of any potential bias	P17-18
Interpretation	20	Give a cautious overall interpretation of results considering objectives, limitations, multiplicity of analyses, results from similar studies, and other relevant evidence	P18
Generalisability	21	Discuss the generalisability (external validity) of the study results	P18
**Other information**			
Funding	22	Give the source of funding and the role of the funders for the present study and, if applicable, for the original study on which the present article is based	P19

*Give information separately for exposed and unexposed groups.

Note: An Explanation and Elaboration article discusses each checklist item and gives methodological background and published examples of transparent reporting. The STROBE checklist is best used in conjunction with this article (freely available on the Web sites of PLoS Medicine at http://www.plosmedicine.org/, Annals of Internal Medicine at http://www.annals.org/, and Epidemiology at http://www.epidem.com/). Information on the STROBE Initiative is available at www.strobe-statement.org.

For peer review only

For peer review only - http://bmjopen.bmj.com/site/about/guidelines.xhtml

Axial length to corneal radius of curvature ratio and refractive error in 4- to 6-year-old Chinese preschoolers: a retrospective cross-sectional study

Journal:	*BMJ Open*
Manuscript ID	bmjopen-2023-075115.R2
Article Type:	Original research
Date Submitted by the Author:	13-Nov-2023
Complete List of Authors:	Tang, Tao; Peking University, Institute of Medical Technology<br>Zhao, Heng; Peking University, Institute of Medical Technology<br>Liu, Duanke; Peking University, Institute of Medical Technology<br>Li, Xuewei; Peking University, Institute of Medical Technology<br>Wang, Kai; Peking University People's Hospital, Department of Ophthalmology & Clinical Centre of Optometry, Peking University People’s Hospital<br>Li, Yan; Peking University People's Hospital, Department of Ophthalmology & Clinical Centre of Optometry, Peking University People’s Hospital<br>Zhao, Mingwei; Peking University People's Hospital, Department of Ophthalmology & Clinical Centre of Optometry, Peking University People’s Hospital
**Primary Subject Heading**:	Ophthalmology
Secondary Subject Heading:	Paediatrics, Public health
Keywords:	OTOLARYNGOLOGY, Paediatric ophthalmology < OPHTHALMOLOGY, Medical ophthalmology < OPHTHALMOLOGY

I, the Submitting Author has the right to grant and does grant on behalf of all authors of the Work (as defined in the below author licence), an exclusive licence and/or a non-exclusive licence for contributions from authors who are: i) UK Crown employees; ii) where BMJ has agreed a CC-BY licence shall apply, and/or iii) in accordance with the terms applicable for US Federal Government officers or employees acting as part of their official duties; on a worldwide, perpetual, irrevocable, royalty-free basis to BMJ Publishing Group Ltd ("BMJ") its licensees and where the relevant Journal is co-owned by BMJ to the co-owners of the Journal, to publish the Work in this journal and any other BMJ products and to exploit all rights, as set out in our licence.

The Submitting Author accepts and understands that any supply made under these terms is made by BMJ to the Submitting Author unless you are acting as an employee on behalf of your employer or a postgraduate student of an affiliated institution which is paying any applicable article publishing charge ("APC") for Open Access articles. Where the Submitting Author wishes to make the Work available on an Open Access basis (and intends to pay the relevant APC), the terms of reuse of such Open Access shall be governed by a Creative Commons licence – details of these licences and which Creative Commons licence will apply to this Work are set out in our licence referred to above.

Other than as permitted in any relevant BMJ Author's Self Archiving Policies, I confirm this Work has not been accepted for publication elsewhere, is not being considered for publication elsewhere and does not duplicate material already published. I confirm all authors consent to publication of this Work and authorise the granting of this licence.

For peer review only

Title Page:

Axial length to corneal radius of curvature ratio and refractive error in 4- to 6-year-old Chinese preschoolers: a retrospective cross-sectional study

Tao Tang[1,2,3,4,5#] **, Heng Zhao**[1,2,3,4,5]

, Duanke Liu[1,2,3,4,5]**, Xuewei Li**[1,2,3,4,5]**, Kai Wang**[1,2,3,4,5*]**, Yan Li** [2,3,4,5*]**, & Mingwei Zhao**[1,2,3,4,5]

[1] Department of Ophthalmology & Clinical Centre of Optometry, Peking University People's Hospital, Beijing, China

[2] Institute of Medical Technology, Peking University Health Science Center, Beijing, China

[3]College of Optometry, Peking University Health Science Center, Beijing, China

[4]Eye Disease and Optometry Institute, Peking University People's Hospital, Beijing, China

[5]Beijing Key Laboratory of the Diagnosis and Therapy of Retinal and Choroid Diseases, Beijing, China

Author Affiliations:

First author: Tao Tang [1,2,3,4,5#]

[1] Department of Ophthalmology & Clinical Centre of Optometry, Peking University People's Hospital, Beijing, China

[2] Institute of Medical Technology, Peking University Health Science Center, Beijing, China

[3]College of Optometry, Peking University Health Science Center, Beijing, China

[4]Eye Disease and Optometry Institute, Peking University People's Hospital, Beijing, China

[5]Beijing Key Laboratory of the Diagnosis and Therapy of Retinal and Choroid Diseases, Beijing, China

For peer review only

Heng Zhao [1, 2, 3, 4, 5]

[1] Department of Ophthalmology & Clinical Centre of Optometry, Peking University People's Hospital, Beijing, China
[2] Institute of Medical Technology, Peking University Health Science Center, Beijing, China
[3] College of Optometry, Peking University Health Science Center, Beijing, China
[4] Eye Disease and Optometry Institute, Peking University People's Hospital, Beijing, China
[5] Beijing Key Laboratory of the Diagnosis and Therapy of Retinal and Choroid Diseases, Beijing, China

Duanke Liu [1, 2, 3, 4, 5]

[1] Department of Ophthalmology & Clinical Centre of Optometry, Peking University People's Hospital, Beijing, China
[2] Institute of Medical Technology, Peking University Health Science Center, Beijing, China
[3] College of Optometry, Peking University Health Science Center, Beijing, China
[4] Eye Disease and Optometry Institute, Peking University People's Hospital, Beijing, China
[5] Beijing Key Laboratory of the Diagnosis and Therapy of Retinal and Choroid Diseases, Beijing, China

Xuewei Li [1, 2, 3, 4, 5]

[1] Department of Ophthalmology & Clinical Centre of Optometry, Peking University People's Hospital, Beijing, China
[2] Institute of Medical Technology, Peking University Health Science Center, Beijing, China
[3] College of Optometry, Peking University Health Science Center, Beijing, China
[4] Eye Disease and Optometry Institute, Peking University People's Hospital, Beijing, China
[5] Beijing Key Laboratory of the Diagnosis and Therapy of Retinal and Choroid Diseases, Beijing, China

Corresponding author: Kai Wang [1, 2, 3, 4, 5*]

For peer review only

[1] Department of Ophthalmology & Clinical Centre of Optometry, Peking University People's Hospital, Beijing, China
[2] Institute of Medical Technology, Peking University Health Science Center, Beijing, China
[3]College of Optometry, Peking University Health Science Center, Beijing, China
[4]Eye Disease and Optometry Institute, Peking University People's Hospital, Beijing, China
[5]Beijing Key Laboratory of the Diagnosis and Therapy of Retinal and Choroid Diseases, Beijing, China

Corresponding author: Yan Li [2,3,4,5 *]
[1]Department of Ophthalmology & Clinical Centre of Optometry, Peking University People's Hospital, Beijing, China
[3]College of Optometry, Peking University Health Science Center, Beijing, China
[4]Eye Disease and Optometry Institute, Peking University People's Hospital, Beijing, China
[5]Beijing Key Laboratory of the Diagnosis and Therapy of Retinal and Choroid Diseases, Beijing, China

Mingwei Zhao [1 , 2 , 3. 4 , 5]
[1] Department of Ophthalmology & Clinical Centre of Optometry, Peking University People's Hospital, Beijing, China
[2] Institute of Medical Technology, Peking University Health Science Center, Beijing, China
[3]College of Optometry, Peking University Health Science Center, Beijing, China
[4]Eye Disease and Optometry Institute, Peking University People's Hospital, Beijing, China
[5]Beijing Key Laboratory of the Diagnosis and Therapy of Retinal and Choroid Diseases, Beijing, China

Short Title: AL/CRC ratio and refractive error in Chinese preschoolers

[*]Corresponding authors:
Kai Wang, Department of Ophthalmology & Clinical Centre of Optometry, Peking University People' Hospital; College of Optometry, Peking University Health Science Center. Beijing, China.
E-mail: wang_kai@bjmu.edu.cn **Cell:** 0086-13810097881, **Tel.:** 0086-010-88325413.

For peer review only

Yan Li, Department of Ophthalmology & Clinical Centre of Optometry, Peking University People' Hospital; College of Optometry, Peking University Health Science Center. Beijing, China.
E-mail: 13801153660@163.com **Cell:** 0086-13801153660

Number of Table: three tables.
Number of Figure: two figures.
Number of Supplemental Table: three Supplemental Tables
Number of Supplemental Figure: two Supplemental Figures

ABSTRACT

Objective To investigate the associations of axial length to corneal radius of curvature (AL/CRC) ratio with refractive error and to determine the effect of AL/CRC ratio on hyperopia reserve and myopia assessment among Chinese preschoolers.

Methods This was a retrospective cross-sectional study evaluated subjects aged 4-6 years. AL and CRC were obtained using a noncontact ocular biometer. Correlation analysis was performed to explore the associations of AL/CRC ratio with spherical equivalent refractive error (SER). The accuracy of AL/CRC ratio for hyperopia reserve and myopia assessment were analysed using cycloplegic refraction by drawing receiver operating characteristic (ROC) curves.

Results The analysis included 1024 participants (537 boys, 52.4%). The mean AL/CRC ratios in hyperopes, emmetropes and myopes were 2.90±0.06, 2.95±0.05 and 3.08±0.07, respectively. The SER was found to be more strongly correlated with AL/CRC ratio (ρ=-0.66, p<0.001) than either AL or CRC alone (ρ=-0.52, p<0.001; ρ=-0.03, p=0.33, respectively). AL/CRC was correlated with SER in hyperopes (ρ=-0.54, p<0.001), emmetropes (ρ=-0.33, p<0.001) and myopes (r=-0.67, p<0.001).

For peer review only

For low hyperopia reserve assessment, the area under the ROC curves of AL/CRC ratio was 0.861 (95% CI: 0.829-0.892), the optimal cut-off value of the AL/CRC ratio was ≥2.955. For myopia assessment, the area under the ROC curves of AL/CRC ratio was 0.954 (95% CI: 0.925-0.982), the optimal cut-off value of the AL/CRC ratio was ≥2.975.

Conclusions The SER showed a better correlation with AL/CRC ratio than either AL or CRC alone, especially in myopes, among 4- to 6-year-old children. These findings indicate that when cycloplegic refraction is unavailable, AL/CRC ratio could be used as an alternative indicator for identifying low hyperopia reserve and myopia among preschoolers, helping clinicians and parents screen children with low hyperopia reserve before primary school in a timely manner.

For peer review only

Strengths and limitations of this study

1. An advantage of this study was the population-based design, with a large sample size of 4- to 6-year-old children.
2. The hyperopic reserve and myopia were assessed by AL/CRC ratio using ROC curves and AUC.
3. This study is cross-sectional and does not pinpoint the temporal correlations between refractive error and the AL/CRC ratio.
4. The sample size of 4-year-old participants was relatively small, which may result in potential for discrepancy.

Key words: myopia, hyperopia reserve, spherical equivalent refractive error, axial length, axial length to corneal radius of curvature ratio

INTRODUCTION

Myopia has become a global public health problem worldwide, especially in East Asian countries such as China, Singapore and South Korea [1-3]. It is predicted that, by 2050, 49.8% of the global population will have myopia and 19.7% will suffer high myopia [4]. It is worth noting that the incidence of myopia has shown a trend toward increasing in those who are younger in recent years. In 2011, the overall prevalence of myopia among children aged 5-14 years in Beijing was 36.7% [5]. In Hong Kong, myopia was 18.3% in children aged 6 years and increased to 61.5% in those aged 12 years in 2018 [6]. In France, the myopic rate was 19.6% in children aged 0 to 9 years in 2013 [7]. For most myopic patients, refractive error tends to stabilize in adulthood, while for a significant proportion of the myopic population, refraction continues to progress in adulthood and develops into pathologic myopia, resulting in various vision-threatening complications such as retinal detachment, glaucoma and cataracts [8-10]. Early monitoring of children’s refractive development and timely control of myopia progression will reduce the risk of myopia-related

complications in the future life.

Ocular biometrics including axial length, corneal curvature and lens power are among the most important factors affecting refractive status of the eyes [11]. During infancy and young childhood, most children have a physiological hyperopia of approximately +2.00 diopters (D), which is defined as hyperopia reserve [12,13]. Balancing changes in axial length and ocular refractive components, including the cornea and the lens, leads to emmetropization [14]. As children grow older, the refraction of the eye develops, and the hyperopia reserve will gradually decrease. Myopia is an ocular disorder characterized by excessive ocular elongation in relation to total ocular power. It has been widely accepted that the myopia shift in refraction in schoolchildren is due to axial elongation [15,16]. When the rate of axial elongation outpaces the changes in optical power of the cornea and the lens, there is a tendency towards myopia onset [17]. For young children, an early reduction in hyperopic reserve might be a risk factor for future myopia development [18]. Zadnik et al. found that children in the United States in grade 1 with less than +0.75 D of hyperopia are at increased risk of myopia formation [18]. Yue et al. also reported that the cut point for myopia for grade 1 students was +0.31 D in the Chinese population [19]. Myopia develops fastest at the age of 6-7 years, and tends to slow down after 11 years [20]. Thus, to prevent the formation of myopia at an early age, clinicians and parents should pay attention to children with low hyperopia reserve and begin to monitor the development of refractive error before primary school.

Typically, cycloplegic refraction is the gold standard for paediatric refractive error diagnosis [21-23], especially in younger children [22,23], largely due to an active accommodation response. However, considering that the side effects of cycloplegia such as allergic conjunctivitis, photophobia and poor near visual acuity [24-26], cause parents or children to be reluctant to undergo cycloplegic refraction. Furthermore, for clinicians who lack regulatory approval for the use of cycloplegics and some patients who are unable to use cycloplegia, cycloplegic refraction may not be performed in such situations. Effective strategies are needed to examine ocular refractive development and diagnose refractive error in young children. Scheiman M et al. reported that the axial length to corneal radius of curvature (AL/CRC) ratio could be a better marker of myopia progression than AL alone [27]. The correlation between refraction and the AL/CRC ratio was stronger than that between refraction and AL [27-29], and the AL/CRC ratio could explain the total variance in refractive error better than AL alone [30,31]. Moreover, the measurement of AL and CRC is noninvasive, and it is easily accepted by children [29]. Thus, although cycloplegic refraction cannot be replaced by any other measurements for the diagnosis of refractive error in children, as an alternative, the AL/CRC ratio can be used as an approximated surrogate for refractive error estimation.

Although many studies have addressed the correlation between the AL/CRC ratio and refractive error in school-aged children and adult population [17,32-35], few population-based studies to date have been performed to focus on the correlation between the AL/CRC ratio and refractive error and evaluate the effect of the AL/CRC ratio for hyperopia reserve and myopia assessment among preschool children in China. Therefore, in this study, we aimed to report the AL/CRC ratio in a Chinese preschool student population. In addition, we examined the correlation between the AL/CRC ratio and refractive error and evaluated the effectiveness of the AL/CRC ratio for refractive error assessment in 4- to 6-year-old Chinese preschoolers.

For peer review only

METHODS

Setting and participants

The study consisting of participants who visited the Peking University People's Hospital optometry centre between January 2020 and September 2022 due to refractive error, was conducted to investigate AL/CRC ratio and refractive error. All the subjects underwent a comprehensive ophthalmological examination and 1024 subjects fulfilled the following inclusion criteria were enrolled in the retrospective cross-sectional study: (1) age of 4 to 6 years old; (2) astigmatism and anisometropia of 1.50 D or less; (3) the monocular best corrected visual acuity (VA) of 0.00 logMAR (6/6) or better; (4) the absence of any ocular and systemic diseases, such as cataracts, glaucoma, and strabismus; (5) cooperate in completing eye examinations. This study was approved by the institutional research ethics committee of Peking University People's Hospital (2021PHB322-001). All examinations and management were performed in accordance with the Declaration of Helsinki, and written informed consent was obtained from the parents.

Measures

Refraction and ocular biometry examinations were performed for all subjects. First, measurement of ocular biometric parameters was performed with an optical biometer. AL was measured with noncontact partial-coherence laser interferometry (IOL Master; Carl Zeiss Meditec, Oberkochen, Germany). A corneal topography system (the Sirius, Italy) was used to obtain the mean K reading (Kmean) and anterior chamber depth (ACD). Five valid readings were taken and averaged. The measurements were taken by the same experienced technical staff, and the mean value of these measurements was taken for statistical analysis. The ocular parameters presented here are the mean ± standard deviation (SD).

After the biometry measurements, all subjects underwent noncycloplegic refraction with an autorefractor (KP8800; Topcon Corp., Tokyo, Japan). This instrument generated five reliable readings of refraction in both eyes; the median reading was used for analysis. Then, cycloplegic refraction was performed for each subject with three drops of 1% cyclopentolate, the first two drops were administered 5 minutes apart, and the third drop was administered 20 minutes later. Cycloplegia was considered complete if the pupil dilated to 6 mm or more and there was no pupillary reflex. The average of five readings automatically performed by the autorefractor was used for analyses. Slit-lamp examination and fundus examination were performed.

Definitions

Refraction was defined as spherical equivalent refraction (SER; SER = spherical power + cylinder power/2). Myopia was defined as SER≤-0.50 D, emmetropia as –0.50 D<SER≤+0.50 D and hyperopia as SER>+0.50 D. Signals from the tear film and retinal pigment epithelium (RPE) are used by noncontact partial-coherence laser interferometry (IOL Master; Carl Zeiss Meditec, Oberkochen, Germany) for axial length measurements. Anterior chamber depth was defined as the distance from the anterior corneal surface to the anterior lens surface. The mean K reading (Kmean = (flat K reading + steep K reading)/2) was the average of the steepest and flattest

For peer review only

meridians. The corneal radius of curvature (CRC) was converted from the Kmean data using the formula CRC=0.3375/Kmean (D)×1000. The axial length to corneal radius of curvature ratio (AL/CRC ratio) was defined as the axial length divided by the mean corneal radius of curvature. Lens power was calculated using the Bennett-Rabbetts method [36] with unknown lens thickness, using measured values for SER, ACD, CRC and AL. The equation was as follows by the formula of Bennett.

$$LP = \frac{L(S_{cv} + K) - 1000n}{(L - ACD - c_{BR})\left(\frac{ACD + c_{BR}}{1000n}(S_{CV} + K) - 1\right)}$$

In this formula, LP is the lens power using the Bennett-Rabbetts method, L is the axial length, S is the spherical equivalent refraction at the corneal vertex, S_{CV}=S/(1-0.014S), K is the mean corneal power, n=1.336, and c_{BR}=2.891±0.778 mm.

Analysis

Statistical analysis was performed using the SPSS statistical software package (Version 22.0, IBM Corp., US). Descriptive statistics were calculated. Continuous data are expressed as the mean ± standard deviation (mean±SD). As biometric data for the right and left eyes were highly correlated (Pearson correlation coefficient, r=0.91), analyses were performed using data for the right eye only. The AL, CRC, AL/CRC ratio, and SER were analysed as continuous variables, and the Kolmogorov–Smirnov test was performed to verify the normality of these variables. To show the correlations between SER and AL, CRC, and the AL/CRC ratio, Spearman's correlation analyses were performed. The relationship between the AL/CRC ratio and other variables was assessed using Spearman's correlation. SER, AL, CRC, AL/CRC ratio, ACD and LP were compared between groups using independent sample one-way ANOVA. Separate linear regression models were constructed with SER as the dependent variable and AL/CRC ratio, AL, and CRC as the main covariate, respectively. The sensitivity, specificity, Youden index, true positive rate, and false positive rate of the AL/CRC ratio and AL for hyperopia reserve and myopia assessment were analysed. The effectiveness of the AL/CR ratio, CRC and AL for hyperopia reserve and myopia assessment was evaluated using receiver operating characteristic (ROC) curves and the area under the curve (AUC) of the ROC curves. All p values were two sided, and a p value of < 0.05 was indicated statistical significance.

Patient and Public Involvement

None.

For peer review only

RESULTS

Demographic characteristics

A total of 1024 children (537 boys and 487 girls) aged 4-6 years (mean 5.77±0.47 years) were included in this study. The average ages of the boys and girls were 5.79 and 5.76 years, respectively. There was no significant difference in sex distribution ($\chi^2 = 2.441$, p=0.118). The mean SER was 0.90±1.11 D, mean AL was 22.59±0.76 mm, mean CRC was 7.74±0.24 mm and mean AL/CR ratio was 2.92±0.08. The detailed demographic characteristics of the subjects are shown in **Supplemental Table 1**.

The histograms of SER, AL, CRC and AL/CRC ratio are shown in **Supplemental Figure 1**. The Kolmogorov–Smirnov test indicated that the frequency distribution was nonnormal for SER, AL, CRC and the AL/CRC ratio (all $p<0.05$). The overall prevalence rates of myopia and high myopia were 7.3% and 0.1%, respectively. The prevalence rates of myopia in subjects aged 4, 5, and 6 years were 4.2% (1/24), 5.4% (10/186), and 7.9% (64/814), respectively.

Table 1 shows the distribution of ocular biometry and the AL/CRC ratio according to the categories of refractive status of subjects. Axial length was significantly longer ($p<0.001$) in myopes (23.74 ± 0.65 mm) than in emmetropes (22.86 ± 0.63 mm) and hyperopes (22.42 ± 0.67 mm) ($p<0.001$). The AL/CRC ratio was also significantly higher in myopes (3.08±0.07) than in emmetropes (2.95±0.05) and hyperopes (2.90±0.06) ($p<0.001$), while CRC and ACD tended to stabilize in different refractive statuses. Emmetropes have the largest LP, while myopes have the smallest LP. **Supplemental Figure 2** shows that AL/CRC ratio correlates with age, and the older the age, the greater the value of AL/CRC ratio.

Table 1. Values of ocular parameters and correlations between AL, CRC, AL/CRC ratio and SER according to refractive status

Parameters	Hyperopes (n=772)	Emmetropes (n=174)	Myopes (n=78)	p value
SER	+1.32±0.73	+0.18±0.30	-1.70±1.05	<0.001

For peer review only

For peer review only - http://bmjopen.bmj.com/site/about/guidelines.xhtml

AL	22.42±0.67	22.86±0.63	23.74±0.65	<0.001
CRC	7.74±0.24	7.76±0.26	7.72±0.21	0.678
AL/CRC	2.90±0.06	2.95±0.05	3.08±0.07	<0.001
ACD	3.35±0.27	3.46±0.23	3.30±0.26	0.140
LP	24.18±1.27	24.31±1.32	22.62±1.28	<0.001
Spearman's correlation coefficients				
SER and AL	-0.41*	-0.08	-0.37*	
SER and CRC	-0.06	0.10	-0.15	
SER and AL/CRC	-0.54*	-0.33*	-0.67*	

SER, spherical equivalent refractive error; AL, axial length; CRC, corneal radius of curvature; AL/CRC ratio, axial length to corneal radius of curvature ratio; LP, lens power; ACD, anterior chamber depth; D, diopter. Significant differences are masked by asterisks.

Correlation analysis between AL, CRC, AL/CRC ratio and SER

Figure 1 shows the scatter plots between AL, CRC, AL/CRC ratio and SER. The SER showed a better correlation with the AL/CRC ratio (Spearman's correlation coefficient, ρ=-0.66, p<0.001) than either AL (ρ=-0.52, p<0.001) or CRC (ρ=-0.03, p=0.33) (**Table 2**). The Spearman's correlation coefficient between SER and the AL/CRC ratio was the highest in myopes (ρ=-0.67, p<0.001), followed by hyperopes (ρ=-0.54, p<0.001) and emmetropes (ρ=-0.33, p<0.001). There was a significant correlation between SER and AL in hyperopes (ρ=-0.41, p<0.001) and myopes (ρ=-0.37, p<0.001), but not in emmetropes (ρ=-0.08, p=0.28). There was no statistical

For peer review only

significance for the correlation between SER and CRC in all refractive groups (all p>0.05). Compared to either AL or CRC, the AL/CRC ratio showed better correlation with SER in all refractive groups (**Table 1**).

Regression analysis between AL, CRC, AL/CRC ratio and SER

Univariate and multiple linear regression analyses were performed in this study (**Table 2**). For SER variance, AL explained 34.5%, whereas the AL/CRC ratio explained 64.5% of the data after adjusting for age and sex. Multiple linear regression adjusted for age and sex showed that a 0.1 increase in the AL/CRC ratio was associated with a -1.19 D change in SER and a 1 mm increase in AL was associated with a -0.87 D change in SER.

Table 2. Association between AL/CRC ratio, AL, and CRC with SER.

Variables	Correlation coefficient	P value	Univariate OR (95% CI)	P value	Gender and age-adjusted Multivariate OR (95% CI)				
					Unstandardised coefficient	95% CI	Standardised coefficient	P value	R^2
AL	-0.52	<0.001	-0.86 (-0.94 to -0.79)	<0.001	-0.869	-0.942 to -0.795	-0.590	<0.001	0.345
CRC	-0.03	0.33	0.15 (-0.14 to 0.43)	0.31	0.148	-0.135 to 0.430	0.032	0.306	0.002
AL/CRC	-0.66	<0.001	-11.73 (-12.27 to -11.19)	<0.001	-11.876	-12.418 to -11.334	-0.809	<0.001	0.645

SER, spherical equivalent refractive error; AL, axial length; CRC, corneal radius of curvature; AL/CRC ratio, axial length to corneal radius of curvature ratio. OR, odds ratio.

Effectiveness of AL, CRC and the AL/CR ratio for hyperopia reserve assessment and myopia assessment

Taking cycloplegic refraction SER ≤ +0.50 D as the standard for diagnosis of the lack of hyperopia reserve [37], the effectiveness of AL, CRC and AL/CR ratio for myopia assessment were analysed (**Figure 2A**). The ROC curves were drawn using AL, CRC and the AL/CR ratio as the index for hyperopia reserve assessment, and the AUCs of the ROC curves were 0.777 (95% confidence interval, CI: 0.741-0.812), 0.494(95% CI: 0.450-0.539) and 0.861 (95% CI: 0.829-0.892), respectively. If AL was used for hyperopia reserve assessment, the optimal cut-off point of the ROC curve was ≥22.615 mm, with a specificity, sensitivity, Youden index, true positive

For peer review only

rate, and false positive rate of 0.785, 0.604, 0.389, 0.604 and 0.215, respectively. If the AL/CR ratio was used for myopia assessment, the optimal cut-off point of the ROC curve was ≥2.955, with a specificity, sensitivity, Youden index, true positive rate, and false positive rate of 0.732, 0.863, 0.595, 0.863, and 0.268, respectively (**Table 3**). When optimal cut-off value of AL/CRC ratio was used to assess the lack of hyperopic reserve, 196 of the 252 participants with low hyperopic reserve were correctly classified using the optimal AL/CRC threshold. Error lack of hyperopia reserve assessment using optimal cut-off value of AL/CRC ratio was 22.22% (56/252) (**Supplemental Table 2**).

Table 3. The sensitivity, specificity, and Youden index of AL and the AL/CRC ratio for hyperopia reserve assessment and myopia assessment.

	SER	Criterion	Cut-off value	Sensitivity	Specificity	Youden index
Hyperopia reserve assessment	≤ +0.50 D	AL (mm)	22.615	0.785	0.604	0.389
		AL/CRC ratio	2.955	0.732	0.863	0.595
	≤ +1.00 D	AL (mm)	22.515	0.677	0.693	0.370
		AL/CRC ratio	2.925	0.670	0.774	0.444
	≤ +1.50 D	AL (mm)	22.495	0.625	0.778	0.403
		AL/CRC ratio	2.895	0.736	0.736	0.472
Myopia assessment	≤ -0.50 D	AL (mm)	23.235	0.800	0.862	0.662
		AL/CRC ratio	2.975	0.894	0.883	0.777

AL, axial length; AL/CRC ratio, axial length to corneal radius of curvature ratio.

Taking cycloplegic refraction SER ≤ −0.50 D as the gold standard for diagnosis of myopia [19], the effectiveness of AL, CRC and AL/CR ratio for myopia assessment were analysed (**Figure 2B**). The ROC curves were drawn using AL, CRC and the AL/CR ratio as the index for myopia assessment, and the AUCs of the ROC curves were 0.898 (95% CI: 0.864-0.932), 0.493(95% CI: 0.434-0.553) and 0.954 (95% CI: 0.925-0.982), respectively. If AL was used for myopia assessment, the optimal cut-off point of the ROC curve was ≥23.235 mm, with a specificity, sensitivity, Youden index, true positive rate, and false positive rate of 0.862, 0.800, 0.662, 0.800 and 0.138, respectively. If the AL/CR ratio was used for myopia assessment, the optimal cut-off point of the ROC curve was ≥2.975, with a specificity, sensitivity, Youden index, true positive rate, and false positive rate of 0.883, 0.894, 0.777, 0.894, and 0.117, respectively (**Table 3**). When optimal cut-off value of AL/CRC ratio was used to assess true myopia, 79 of the 85 participants with myopia were correctly classified using the optimal AL/CRC threshold. Error true myopia assessment using optimal cut-off value of AL/CRC ratio was 7.06% (6/85) (**Supplemental Table 2**).

For peer review only

For peer review only - http://bmjopen.bmj.com/site/about/guidelines.xhtml

DISCUSSION

This study found that AL/CRC ratio was correlated with SER in hyperopes, emmetropes and myopes, while AL showed correlation with SER in hyperopes and myopes, but not in emmetropes. The SER showed a better correlation with the AL/CRC ratio than either AL or CRC alone in different refractive statuses, especially in myopes, among 4- to 6-year-old children. The effect of the AL/CR ratio for low hyperopia reserve and myopia assessment was higher than that of either AL or CRC alone. These findings indicate that when cycloplegic refraction is unavailable, AL/CRC ratio can be used as an alternative indicator for monitoring low hyperopia reserve and myopia among preschoolers.

Myopia is a common disorder of the eyes and has become a global public health concern [38,39]. During the past several decades, the prevalence of myopia in children has increased rapidly, and the age at onset of myopia has decreased. Chinese children tend to have a higher prevalence of myopia than children in other countries and of other ethnicities. The prevalence of myopia exceeds 60% among 12 years of age in China after primary school, reaches nearly 80% at 16 years of age after junior high school, and surpasses 90% in university students [1,4,13]. Notably, the incidence of myopia increased among preschool children in recent years. The prevalence of myopia was 17.6% in children aged 6 years in Hong Kong [40]. A study in Shanghai of 7166 Chinese children aged 4 to 6 years reported that the prevalence of myopia ranged from 5.7% to 7.1% [41]. In this study, the prevalence of myopia in preschoolers increased from 4.2% at 4 years of age to 7.9% at 6 years of age. Although myopia tends to stabilize after adulthood, children with a younger age at myopia onset are prone to develop high myopia and have a high risk of suffering from vision-threatening complications. In view of this condition, it is urgent to carry out early vision screening and refractive error assessment among preschool children. Ocular biometrics have been used to estimate the progression of refractive error [29,34]. The AL/CRC ratio summarizes the overall relationship between AL and CRC and minimizes variability in AL and CRC, thus, it has been considered as a more robust index to evaluate the refractive status of the eye [17]. Moreover, the AL/CRC ratio also correlates with refractive error more strongly than AL or CRC alone [28,31]. Therefore, the AL/CRC ratio could be useful in determining refractive error and monitoring myopia progression.

Changes in AL and CRC are important ocular biometrics affecting refractive status [34]. Previous studies have indicated that CRC may be actively modulated to regulate refractive development, and the onset of myopia is due to a failure of corneal compensation for axial elongation [11,33]. In this study, we found that there was no correlation between CRC and refractive error, while AL, or more strikingly, the AL/CRC ratio had a significant correlation with refractive error. This finding suggests that AL has a larger effect on refractive error than CRC. Grosvenor et al. was the first to report the correlation between the AL/CRC ratio and refractive error and studied the role of the AL/CRC ratio in determining the refractive state of the eye [42]. They demonstrated that the AL/CRC ratio was the most important biometric factor in myopia. Some researchers further confirmed Grosvenor’s findings [29,43,44]. The distribution of AL/CRC ratio has been reported in different countries and

For peer review only - http://bmjopen.bmj.com/site/about/guidelines.xhtml

age populations. A summary of AL/CRC ratio in other studies is shown in **Supplemental Table 3**. The average values reported for the AL/CRC ratio range from 2.81 to 3.13. On the one hand, differences in age, race, included subjects, measurement methods and techniques may account for the disagreement in the various studies. On the other hand, we can find that the older age, the greater value of AL/CRC ratio. **Supplemental Figure 2** also confirms this result. This finding indicates that, in addition to refraction, age also affects the value of AL/CRC ratio. The AL/CRC ratio has been proposed as a calculated biometric parameter that correlates better with SER than AL, and the AL/CRC ratio could explain the total variance in SER better than AL alone [31]. This is because the AL/CRC ratio provides a rough measure of the degree of matching between AL and corneal power. In this study, we also found a better correlation between the AL/CRC ratio and SER in children aged 4-6 years compared to that of AL or CRC alone, especially in children with myopia. It is possibly suggested that the AL/CRC ratio is a more useful marker of progress towards myopic refractions than absolute ocular biometric values such as AL or CRC. Moreover, the value of the AL/CRC ratio was negatively correlated with SER. As demonstrated in this study, the AL/CRC ratio was significantly higher in myopes than in emmetropes and hyperopes, which was consistent with studies performed by Foo et al. and Guo et al. [28,31]. This finding indicates that the greater the myopic refraction, the larger the AL/CRC ratio.

A previous study reported that a high AL/CRC ratio of no less than 3.0 has been considered an indicator for the onset of myopia [42]. High axial length to corneal radius ratio is a risk factor in the development of myopia. When the AL/CRC ratio was higher than 3.0, it was considered that other ocular parameters were not able to compensate for the change in refraction caused by the elongation of AL, thereby inducing myopia. As shown in this study, the area under the ROC of the AL/CR ratio for myopia assessment was 0.954, and the optimal cut-off value is ≥2.975, which was smaller than that in the study performed by Mu et al. [29]. This result indicates that for children aged 4 to 6 years, the risk of myopia is likely to occur when the AL/CRC ratio is greater than 2.975. However, it is worth noting that this AL/CRC threshold is still subject to some degree of error assessment in the evaluation of true myopia. We also conducted an evaluation of the effectiveness of the AL/CRC ratio, CRC and AL for hyperopia reserve assessment in this population. Previous study has reported that hyperopia reserve is a potential indicator of myopia [19]. There is a progressive shift in mean refraction from roughly + 2.00 D at 3 months of age to approximately + 1.00 D at 6.5 years of age [45]. Then emmetropization continues at a slower rate after 6 years of age. Zadnik et al. revealed that children in the United States in grade 1 with less than +0.75 D of hyperopia are at increased risk of myopia onset [18]. Yue et al. also reported that the cut point for myopia for grade 1 students was +0.31 D in the Chinese population [19]. Li et al.'s [41] findings showed that the 5-year cumulative incidence of myopia was 4.6%, 26.3%, 52.3%, 78.6%, 92.6% and 94.3%, respectively, corresponding to students with a baseline hyperopia reserve of >+2.00 D, +1.50 D to +2.00 D, +1.00 D to +1.50 D, +0.50 D to +1.00 D, 0.00 D to +0.50 D and -0.50 D to 0.00 D, respectively. The lower the hyperopia reserve in young children, the higher the incidence of myopia onset. In our study, taking cycloplegic refraction SER ≤ +0.50 D as the standard for diagnosis of the lack of hyperopia reserve, the area under the ROC curve of the AL/CR ratio for the lack of hyperopia reserve assessment was greater than that of AL and CRC and the optimal cut-off value of the AL/CRC ratio was ≥2.955. Although this optimal AL/CRC threshold may have error assessment for preschoolers, clinicians and parents could monitor the hyperopia reserve of young children by regularly measuring the axial length and corneal curvature when cycloplegic refraction is unavailable.

For peer review only - http://bmjopen.bmj.com/site/about/guidelines.xhtml

When the AL/CRC ratio is greater than 2.955 among preschool children, clinicians and parents should pay attention to the children with less hyperopia reserve and begin to take measures to prevent myopia formation.

In the present study, AL alone contributed 34.5% of the variance in SER, whereas the AL/CR ratio accounted for 64.5%. Guo et al. study [31] found that AL alone contributed 18.6% of the variance in SER, whereas the AL/CR ratio accounted for 39.8% in the study population of preschoolers aged 3-6 years old. He et al. [30] reported that AL/CR ratio and AL explained 66.4% and 43.1% of the variance in SER, respectively, in a group of Chinese children 6 to 12 years of age. This suggested that the AL/CRC ratio is more closely related to refractive error than AL or CRC alone. Moreover, we found a linear increase in AL/CRC ratio from hyperopia towards myopia. The corresponding increase in SER of -1.1 9D with every 0.1 increase in the AL/CRC ratio in this study is larger than -0.74 D reported by researchers in Singapore [28] and -0.89D reported among the Nigerian population [33], but slightly lower than -1.21 D reported among northern Iranian subjects [17]. The above findings revealed that in addition to AL and CRC, there are other potential factors that influence SER results. Thus, the AL/CRC ratio cannot completely replace cycloplegic refraction in determining refraction in children. However, cycloplegic refraction is easily influenced by the cooperation and compliance of the examinees, especially in younger children, which is not feasible for large-scale myopia assessment and long-term refractive development follow-up. For this reason, the AL/CRC ratio could be used as an alternative indicator for refraction estimation, and it may be more accurate than AL. In addition, measurement of AL and CRC and determination of the AL/CRC ratio are relatively easy and can be conducted by a technician using noncontact, objective, and automated instruments. The application of the AL/CRC ratio reference as an indicator of refractive errors has the effect of simplifying the examination procedure and reducing unnecessary cycloplegia, especially when cycloplegic refraction is difficult to perform in 4- to 6-year-old children.

There were some limitations in this study. First, because this study is cross-sectional and does not pinpoint the temporal correlations between refractive error and the AL/CRC ratio, it is impossible to rule out the possibility of observational and inclusionary biases due to the retrospective study design. Admittedly, longitudinal studies would be needed to conduct to evaluate intraindividual discrepancy. In addition, the data from Beijing may not be representative for the rural areas and other regions in China. Last, the sample size of the 4-year-old participants (N=24) was relatively small. Although our findings are applicable to this population as well, there is potential for discrepancy. We will further increase the sample size of this population to increase the accuracy and reliability of the results of this study.

In conclusion, our study found that SER showed a better correlation with the AL/CRC ratio than either AL or CRC alone in different refractive statuses, especially in myopic children, among 4- to 6-year-old Chinese children. The AL/CRC ratio could explain the total variance in refractive error better than AL alone. The effect of the AL/CR ratio for assessing the lack of hyperopia reserve and for the myopia assessment was higher than that of AL and CRC. These findings indicate that when cycloplegic refraction is unavailable, the AL/CRC ratio could be used as an alternative indicator for identifying low hyperopia reserve and myopia among preschoolers, helping clinicians and parents screen children with low hyperopia reserve before primary school in a timely manner. In the future, longitudinal cohort designs will be conducted for optimal study of the effect of the AL/CRC ratio on hyperopia reserve and myopia assessment in preschool children.

For peer review only

Declarations

Authors' contributions

TT developed the concept, collected data, performed the experiments and data analysis, wrote the main manuscript text and prepared Figures 1-2, Tables 1-3, Supplemental Figures 1-2, Supplemental Tables 1-3; HZ collected data and prepared Figure 2; DL and XL collected data; KW and YL developed the concept and designed the experiments; MZ developed the study conception. TT wrote the first draft of the manuscript. TT, KW and YL made critical revisions. All authors reviewed and approved the final draft. TT is the guarantor and responsible for the overall content.

Funding

This work was supported by: National Natural Science Foundation of China (Grant Nos. 82371087 and 82171092), Capital's Funds for Health Improvement and Research (No. 2022-1G-4083), the National Key R&D Program of China (No.2020YFC2008200 and No.2021YFC2702100).

Competing interests

The authors declare that they have no conflicts of interest.

Patient consent for publication

Not applicable.

Ethics approval

This study was approved by the institutional research ethics committee of Peking University People's Hospital (2021PHB322-001). All examinations and management were performed in accordance with the Declaration of Helsinki, and written informed consent was obtained from the parents.

Availability of data and materials

All data generated or analysed during this study are included in this published article.

For peer review only

Acknowledgements

None.

Corresponding author

Correspondence to Kai Wang and Yan Li

Data Availability Statement

All data generated or analysed during this study are included in this article and its online supplementary material. Further inquiries can be directed to the corresponding author.

References

1. Ma Y, Qu X, Zhu X, et al. Age-Specific Prevalence of Visual Impairment and Refractive Error in Children Aged 3-10 Years in Shanghai, China. Invest Ophthalmol Vis Sci. 2016;57(14):6188-6196.
2. Koh V, Yang A, Saw SM, et al. Differences in Prevalence of Refractive Errors in Young Asian Males in Singapore between 1996–1997 and 2009–2010. Ophthalmic Epidemiology. 2014;21(4):247-255.
3. Jung SK, Lee JH, Kakizaki H, Jee D. Prevalence of myopia and its association with body stature and educational level in 19-year-old male conscripts in seoul, South Korea. Invest Ophthalmol Vis Sci. 2012 Aug 15;53(9):5579-83.
4. Holden BA, Fricke TR, Wilson DA, et al. Global Prevalence of Myopia and High Myopia and Temporal Trends from 2000 through 2050. Ophthalmology. 2016;123(5):1036-1042.
5. Lyu Y, Zhang H, Gong Y, et al. Prevalence of and factors associated with myopia in primary school students in the Chaoyang District of Beijing, China. Jpn J Ophthalmol. 2015;59(6):421-429.
6. Choy BNK, You Q, Zhu MM, Lai JSM, Ng ALK, Wong IYH. Prevalence and associations of myopia in Hong Kong primary school students. Jpn J Ophthalmol. 2020;64(4):437-449.
7. Matamoros E, Ingrand P, Pelen F, et al. Prevalence of Myopia in France: A Cross-Sectional Analysis. Medicine (Baltimore). 2015;94(45):e1976.
8. Lee JY, Sung KR, Han S, Na JH. Effect of myopia on the progression of primary open-angle glaucoma. Invest Ophthalmol Vis Sci. 2015;56(3):1775-1781.
9. Ohno-Matsui K, Wu PC, Yamashiro K, et al. IMI Pathologic Myopia. Invest Ophthalmol Vis Sci. 2021;62(5):5.
10. Haarman AEG, Enthoven CA, Tideman JWL, Tedja MS, Verhoeven VJM, Klaver CCW. The Complications of Myopia: A Review and Meta-Analysis. Invest

For peer review only

Ophthalmol Vis Sci. 2020;61(4):49.

11. Mutti DO, Mitchell GL, Jones LA, et al. Axial growth and changes in lenticular and corneal power during emmetropization in infants. Invest Ophthalmol Vis Sci. 2005;46(9):3074-3080.

12. Flitcroft DI. Emmetropisation and the aetiology of refractive errors. Eye (Lond). 2014;28(2):169-179.

13. Song Y, Zhu S, Yang B, et al. Accommodation and binocular vision changes after wearing orthokeratology lens in 8- to 14-year-old myopic children. Graefes Arch Clin Exp Ophthalmol. 2021;259(7):2035-2045.

14. Rozema J, Dankert S, Iribarren R. Emmetropization and nonmyopic eye growth. Surv Ophthalmol. 2023; 68(4):759-783.

15. Ojaimi E, Rose KA, Morgan IG, et al. Distribution of ocular biometric parameters and refraction in a population-based study of Australian children. Invest Ophthalmol Vis Sci. 2005;46(8):2748-2754.

16. Chamberlain P, Lazon de la Jara P, Arumugam B, Bullimore MA. Axial length targets for myopia control. Ophthalmic Physiol Opt. 2021 May;41(3):523-531.

17. Hashemi H, Khabazkhoob M, Miraftab M, Emamian MH, Shariati M, Abdolahi-Nia T, et al. Axial length to corneal radius of curvature ratio and refractive errors. J Ophthalmic Vis Res. 2013 Jul;8(3):220-6.

18. Zadnik K, Sinnott LT, Cotter SA, et al. Prediction of Juvenile-Onset Myopia. JAMA Ophthalmol. 2015;133(6):683-689.

19. Yue Y, Liu X, Yi S, Liu B, Yi H, Li H. High prevalence of myopia and low hyperopia reserve in 4411 Chinese primary school students and associated risk factors. BMC Ophthalmol. 2022;22(1):212.

20. Hyman L, Gwiazda J, Hussein M, Norton TT, Wang Y, Marsh-Tootle W, et al. Relationship of age, sex, and ethnicity with myopia progression and axial elongation in the correction of myopia evaluation trial. Arch Ophthalmol. 2005 Jul;123(7):977-87.

21. Morgan IG, Iribarren R, Fotouhi A, Grzybowski A. Cycloplegic refraction is the gold standard for epidemiological studies. Acta Ophthalmol. 2015;93(6):581-585.

22. Li T, Zhou X, Zhu J, Tang X, Gu X. Effect of cycloplegia on the measurement of refractive error in Chinese children. Clin Exp Optom. 2019;102(2):160-165.

23. Hu YY, Wu JF, Lu TL, et al. Effect of cycloplegia on the refractive status of children: the Shandong children eye study. PLoS One. 2015;10(2):e0117482.

24. Chia A, Chua WH, Cheung YB, et al. Atropine for the treatment of childhood myopia: safety and efficacy of 0.5%, 0.1%, and 0.01% doses (Atropine for the Treatment of Myopia 2). Ophthalmology. 2012;119(2):347-354.

25. Gong Q, Janowski M, Luo M, et al. Efficacy and Adverse Effects of Atropine in Childhood Myopia: A Meta-analysis. JAMA Ophthalmol. 2017;135(6):624-630.

26. Wei S, Li SM, An W, et al. Safety and Efficacy of Low-Dose Atropine Eyedrops for the Treatment of Myopia Progression in Chinese Children: A Randomized

For peer review only

Clinical Trial. JAMA Ophthalmol. 2020;138(11):1178-1184.

27. Scheiman M, Gwiazda J, Zhang Q, et al. Longitudinal changes in corneal curvature and its relationship to axial length in the Correction of Myopia Evaluation Trial (COMET) cohort. J Optom. 2016;9(1):13-21.
28. Foo VH, Verkicharla PK, Ikram MK, Chua SY, Cai S, Tan CS, et al. Axial Length/Corneal Radius of Curvature Ratio and Myopia in 3-Year-Old Children. Transl Vis Sci Technol. 2016;5(1):5.
29. Mu J, Zeng D, Fan J, et al. The accuracy of the axial length and axial length/corneal radius ratio for myopia assessment among Chinese children. Front Pediatr. 2022;10:859944.
30. He X, Zou H, Lu L, et al. Axial length/corneal radius ratio: association with refractive state and role on myopia detection combined with visual acuity in Chinese schoolchildren. PLoS One. 2015;10(2):e0111766.
31. Guo X, Fu M, Ding X, Morgan IG, Zeng Y, He M. Significant Axial Elongation with Minimal Change in Refraction in 3- to 6-Year-Old Chinese Preschoolers: The Shenzhen Kindergarten Eye Study. Ophthalmology. 2017;124(12):1826-1838.
32. Jong M, Sankaridurg P, Naduvilath TJ, Li W, He M. The Relationship between Progression in Axial Length/Corneal Radius of Curvature Ratio and Spherical Equivalent Refractive Error in Myopia. Optom Vis Sci. 2018;95(10):921-929.
33. Badmus SA, Ajaiyeoba AI, Adegbehingbe BO, Onakpoya OH, Adeoye AO. Axial length/corneal radius of curvature ratio and refractive status in an adult Nigerian population. Niger J Clin Pract. 2017;20(10):1328-1334.
34. Iyamu E, Iyamu J, Obiakor CI. The role of axial length-corneal radius of curvature ratio in refractive state categorization in a nigerian population. ISRN Ophthalmol. 2011;2011:138941.
35. Warrier S, Wu HM, Newland HS, et al. Ocular biometry and determinants of refractive error in rural Myanmar: the Meiktila Eye Study. Br J Ophthalmol. 2008;92(12):1591-1594.
36. Rozema JJ, Atchison DA, Tassignon MJ. Comparing methods to estimate the human lens power. Invest Ophthalmol Vis Sci. 2011;52(11):7937-7942.
37. Li SM, Wei S, Atchison DA, et al. Annual Incidences and Progressions of Myopia and High Myopia in Chinese Schoolchildren Based on a 5-Year Cohort Study. Invest Ophthalmol Vis Sci. 2022;63(1):8.
38. Foster PJ, Jiang Y. Epidemiology of myopia. Eye (Lond). 2014;28(2):202-208.
39. Dolgin E. The myopia boom. Nature. 2015;519(7543):276-8.
40. Lam CS, Lam CH, Cheng SC, Chan LY. Prevalence of myopia among Hong Kong Chinese schoolchildren: changes over two decades. Ophthalmic Physiol Opt. 2012;32(1):17-24.

For peer review only

For peer review only - http://bmjopen.bmj.com/site/about/guidelines.xhtml

41. Li T, Zhou X, Chen X, Qi H, Gao Q. Refractive Error in Chinese Preschool Children: The Shanghai Study. Eye Contact Lens. 2019;45(3):182-187.
42. Grosvenor T. High axial length/corneal radius ratio as a risk factor in the development of myopia. Am J Optom Physiol Opt. 1988 Sep;65(9):689-96.
43. Matsumura S, Dannoue K, Kawakami M, et al. Prevalence of Myopia and Its Associated Factors Among Japanese Preschool Children. Front Public Health. 2022;10:901480.
44. Liu S, Chen J, Wang J, Zhu Z, Zhang J, Zhang B, Yang J, Du L, Zhu J, Zou H, He X, Xu X. Cutoff values of axial length/corneal radius ratio for determining myopia vary with age among 3-18 years old children and adolescents. Graefes Arch Clin Exp Ophthalmol. 2023.
45. Mutti DO, Sinnott LT, Lynn Mitchell G, et al. Ocular Component Development during Infancy and Early Childhood. Optom Vis Sci. 2018;95(11):976-985.

Legends:

Figure 1. Correlation analysis between AL, CRC, AL/CRC ratio and SER. SER, spherical equivalent refractive error; AL, axial length; CRC, corneal radius of curvature; AL/CRC ratio, axial length to corneal radius of curvature ratio, D, diopter.

Figure 2. Comparison of the effectiveness of AL, CRC and the AL/CR ratio for hyperopia reserve assessment (A) and myopia assessment (B). AL, axial length; CRC, corneal radius of curvature; AL/CRC ratio, axial length to corneal radius of curvature ratio; AUC, area under curve.

For peer review only

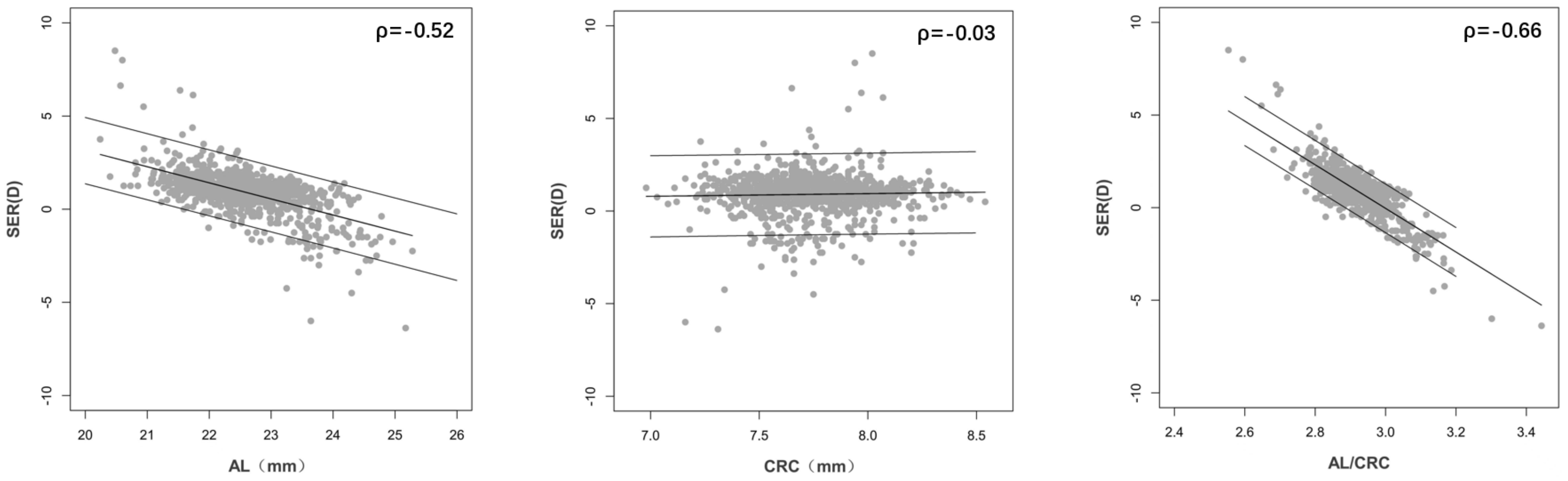

Figure 1. Correlation analysis between AL, CRC, AL/CRC ratio and SER. SER, spherical equivalent refractive error; AL, axial length; CRC, corneal radius of curvature; AL/CRC ratio, axial length to corneal radius of curvature ratio, D, diopter.

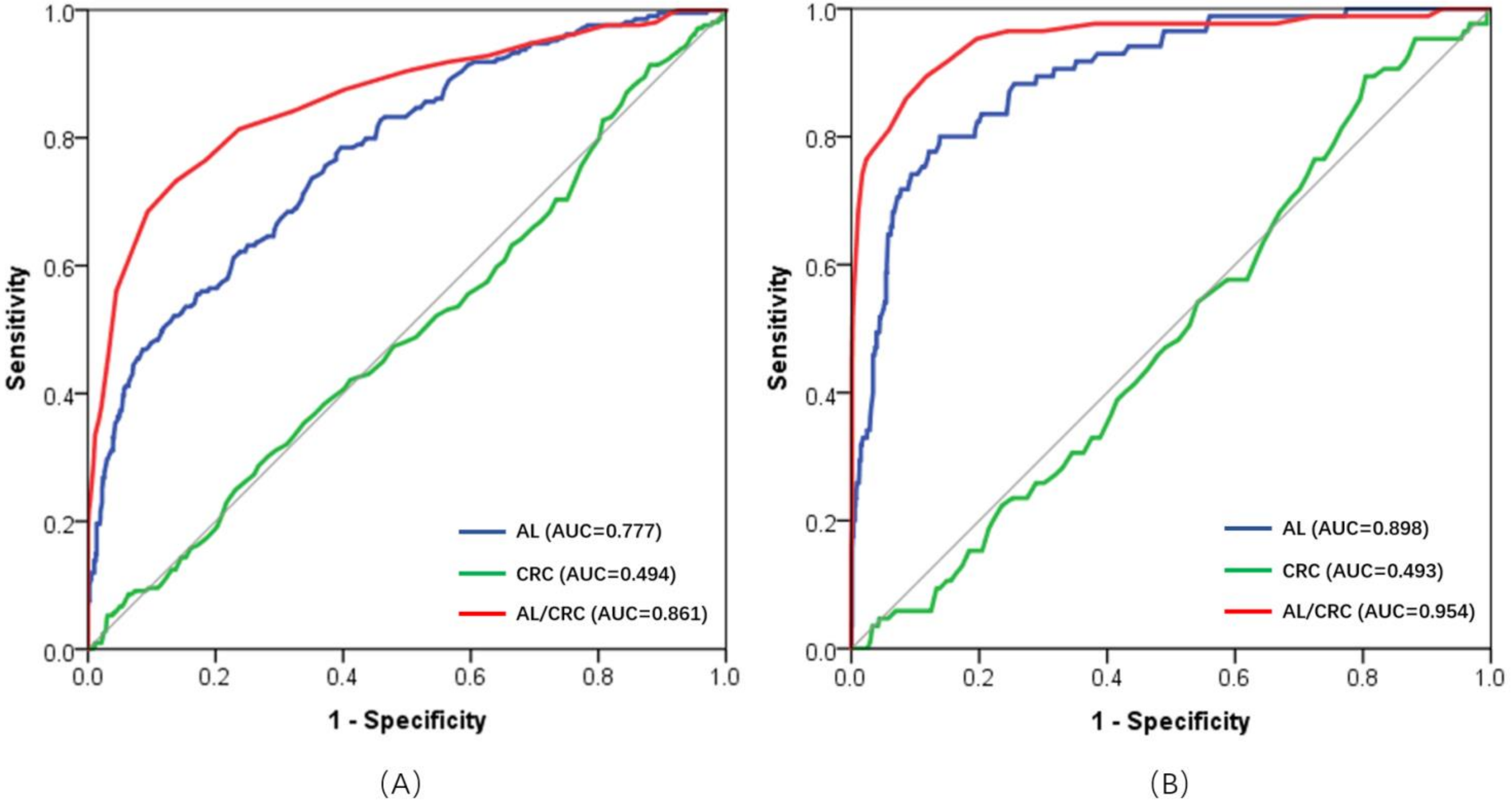

Figure 2. Comparison of the effectiveness of AL, CRC and the AL/CR ratio for hyperopia reserve assessment (A) and myopia assessment (B). AL, axial length; CRC, corneal radius of curvature; AL/CRC ratio, axial length to corneal radius of curvature ratio; AUC, area under curve.

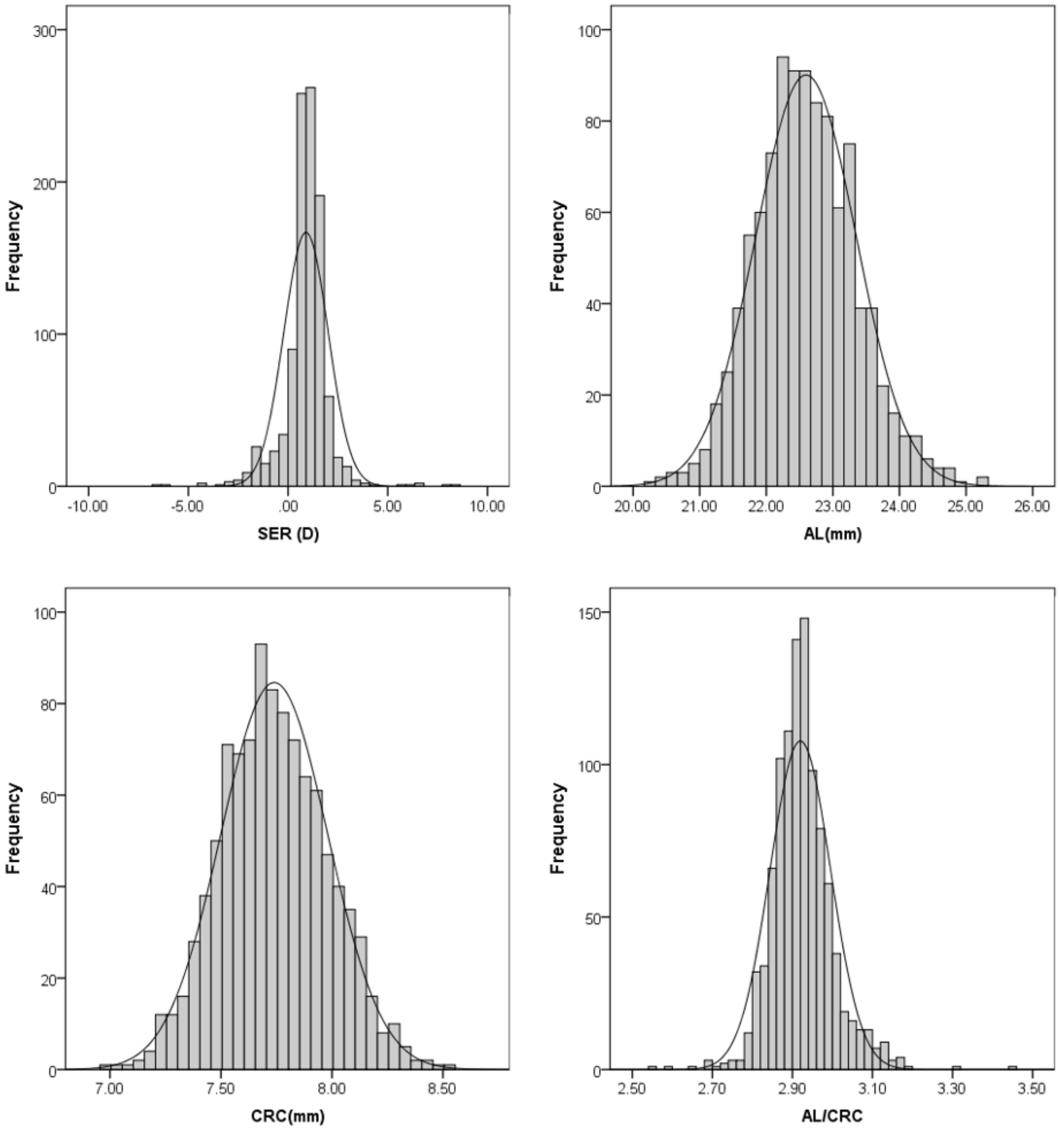

Supplemental Figure 1. Histograms showing distributions of SER, AL, CRC and AL/CRC ratio. SER, spherical equivalent refractive error; AL, axial length; CRC, corneal radius of curvature; AL/CRC ratio, axial length to corneal radius of curvature ratio; D, diopter.

For peer review only - http://bmjopen.bmj.com/site/about/guidelines.xhtml

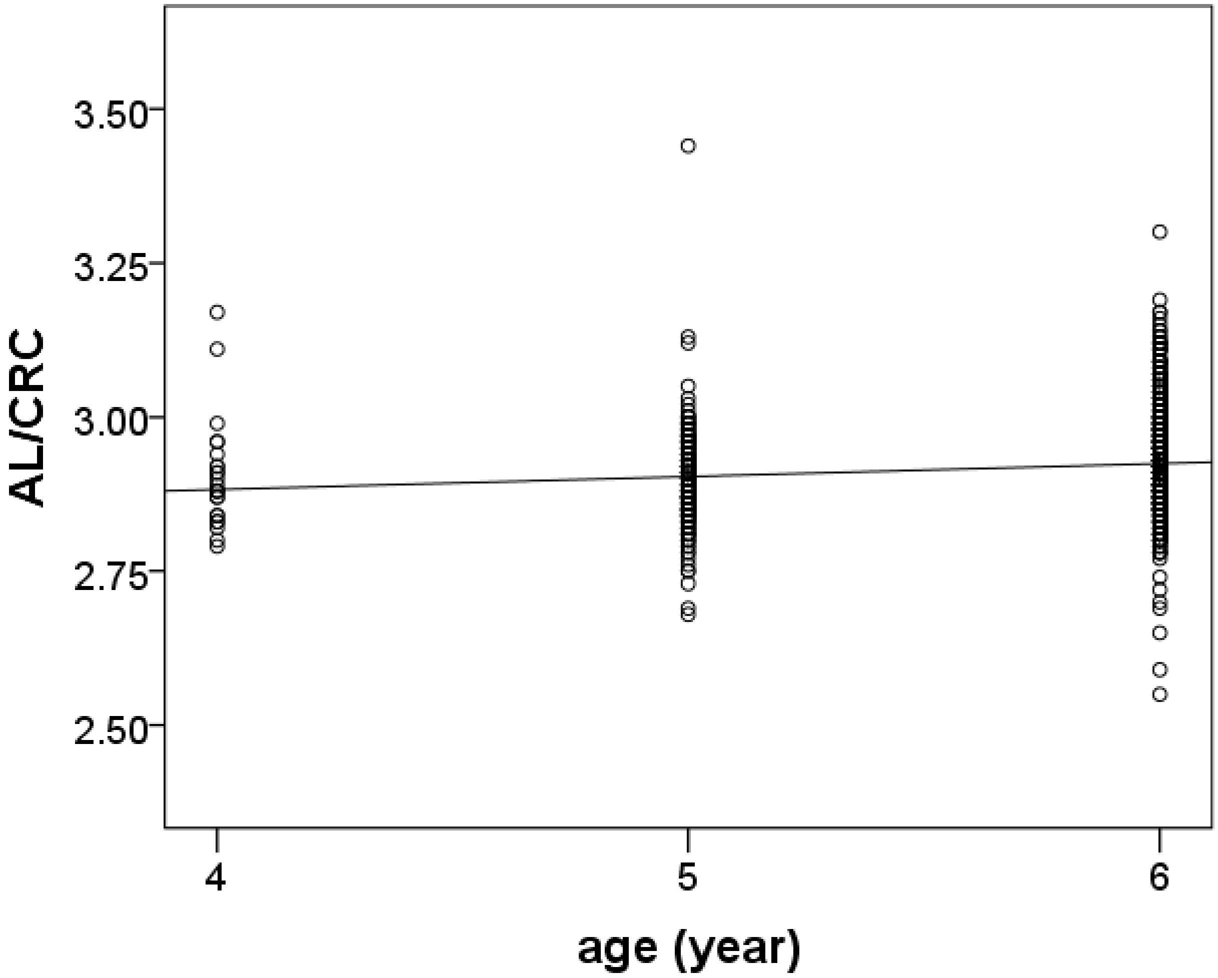

Supplemental Figure 2. Scatterplot of AL/CRC ratio and age. AL/CRC, axial length to corneal radius of curvature ratio.

Supplemental Table 1. Demographic and biometric data of included participants

Parameters	Total, N=1024		Boys, N=537	Girls, N=487	P value
	Mean±SD	Range	Mean±SD	Mean±SD	
Age (years)	5.77±0.47	4-6	5.85±0.35	5.68±0.56	<0.001
SER (D)	0.90±1.11	-6.38 to 8.50	0.90±1.09	0.90±1.13	0.922
AL (mm)	22.59±0.76	20.24 to 25.28	22.62±0.77	22.57±0.74	0.350
CRC (mm)	7.74±0.24	6.98 to 8.54	7.74±0.24	7.74±0.24	0.805
AL/CRC	2.92±0.08	2.65 to 3.17	2.92±0.08	2.92±0.08	0.141
LP (D)	24.09±1.35	19.68 to 28.68	24.02±1.34	24.16±1.35	0.117
ACD (mm)	3.37±0.27	2.09 to 5.37	3.38±0.27	3.34±0.27	0.028

SER, spherical equivalent refractive error; AL, axial length; CRC, corneal radius of curvature; AL/CRC, axial length to corneal radius of curvature ratio; LP, lens power; ACD, anterior chamber depth; D, diopter.

For peer review only

Supplemental Table 2. Effect of optimal cut-off value of AL/CRC ratio for the lack of hyperopia reserve assessment and true myopia assessment.

	Criterion	Total Data	True assessment	Error assessment (%)
Lack of hyperopia reserve assessment	AL/CRC ratio ≥2.955	252	196	56 (22.22%)
True myopia assessment	AL/CRC ratio ≥2.975	85	79	6 (7.06%)

AL/CRC ratio, axial length to corneal radius of curvature ratio. Error assessment (%), using optimal cut-off value of AL/CRC ratio to assess the probability of errors in lack of hyperopia reserve or true myopia.

For peer review only

Supplemental Table 3. Summary of studies reporting the AL, CRC, and AL/CRC ratio.

Origin	N	Age (years)	SER (D)	AL/CRC	AL	CRC
Australia [15]	1765	5.5-8.4	+1.26	2.906	22.61	7.70
Iran [17]	4820	40-64	-	3.033	23.14	7.63
China [19]	4411	7-10	-0.13	2.97	23.27	7.82
Singapore [28]	349	3	+0.88	2.81	21.73	7.72
Nigerian [34]	70	20-39	-0.24	3.03	23.74	7.84
Myanmar [35]	1498	40-70	-1.22	2.99	22.76	7.62
Japan [43]	457	4-6	0.13	2.88	22.35	7.76
Current study	1024	4-6	+0.90	2.92	22.59	7.74

SER, spherical equivalent refractive error; AL, axial length; CRC, corneal radius of curvature; AL/CRC ratio, axial length to corneal radius of curvature ratio.

For peer review only

STROBE Statement—Checklist of items that should be included in reports of ***cross-sectional studies***

	Item No	Recommendation	Page No
**Title and abstract**	1	(*a*) Indicate the study's design with a commonly used term in the title or the abstract	P1
		(*b*) Provide in the abstract an informative and balanced summary of what was done and what was found	P4-5
**Introduction**			
Background/rationale	2	Explain the scientific background and rationale for the investigation being reported	P5-6
Objectives	3	State specific objectives, including any prespecified hypotheses	P6
**Methods**			
Study design	4	Present key elements of study design early in the paper	P6
Setting	5	Describe the setting, locations, and relevant dates, including periods of recruitment, exposure, follow-up, and data collection	P6-7
Participants	6	(*a*) Give the eligibility criteria, and the sources and methods of selection of participants	P6-7
Variables	7	Clearly define all outcomes, exposures, predictors, potential confounders, and effect modifiers. Give diagnostic criteria, if applicable	P7
Data sources/ measurement	8*	For each variable of interest, give sources of data and details of methods of assessment (measurement). Describe comparability of assessment methods if there is more than one group	*P7*
Bias	9	Describe any efforts to address potential sources of bias	P7
Study size	10	Explain how the study size was arrived at	P7
Quantitative variables	11	Explain how quantitative variables were handled in the analyses. If applicable, describe which groupings were chosen and why	P7
Statistical methods	12	(*a*) Describe all statistical methods, including those used to control for confounding	P8
		(*b*) Describe any methods used to examine subgroups and interactions	
		(*c*) Explain how missing data were addressed	P8
		(*d*) If applicable, describe analytical methods taking account of sampling strategy	
		(*e*) Describe any sensitivity analyses	
**Results**			
Participants	13*	(a) Report numbers of individuals at each stage of study—eg numbers potentially eligible, examined for eligibility, confirmed eligible, included in the study, completing follow-up, and analysed	P8
		(b) Give reasons for non-participation at each stage	
		(c) Consider use of a flow diagram	
Descriptive data	14*	(a) Give characteristics of study participants (eg demographic, clinical, social) and information on exposures and potential confounders	P8
		(b) Indicate number of participants with missing data for each variable of interest	
Outcome data	15*	Report numbers of outcome events or summary measures	P8
Main results	16	(*a*) Give unadjusted estimates and, if applicable, confounder-adjusted estimates and their precision (eg, 95% confidence interval). Make clear which confounders were adjusted for and why they were included	P8-14

For peer review only - http://bmjopen.bmj.com/site/about/guidelines.xhtml

		(*b*) Report category boundaries when continuous variables were categorized	
		(*c*) If relevant, consider translating estimates of relative risk into absolute risk for a meaningful time period	
Other analyses	17	Report other analyses done—eg analyses of subgroups and interactions, and sensitivity analyses	
**Discussion**			
Key results	18	Summarise key results with reference to study objectives	P15-17
Limitations	19	Discuss limitations of the study, taking into account sources of potential bias or imprecision. Discuss both direction and magnitude of any potential bias	P17-18
Interpretation	20	Give a cautious overall interpretation of results considering objectives, limitations, multiplicity of analyses, results from similar studies, and other relevant evidence	P18
Generalisability	21	Discuss the generalisability (external validity) of the study results	P18
**Other information**			
Funding	22	Give the source of funding and the role of the funders for the present study and, if applicable, for the original study on which the present article is based	P19

*Give information separately for exposed and unexposed groups.

Note: An Explanation and Elaboration article discusses each checklist item and gives methodological background and published examples of transparent reporting. The STROBE checklist is best used in conjunction with this article (freely available on the Web sites of PLoS Medicine at http://www.plosmedicine.org/, Annals of Internal Medicine at http://www.annals.org/, and Epidemiology at http://www.epidem.com/). Information on the STROBE Initiative is available at www.strobe-statement.org.

For peer review only

BMJ Open

Axial length to corneal radius of curvature ratio and refractive error in 4- to 6-year-old Chinese preschoolers: a retrospective cross-sectional study

Journal:	*BMJ Open*
Manuscript ID	bmjopen-2023-075115.R3
Article Type:	Original research
Date Submitted by the Author:	20-Nov-2023

Complete List of Authors:	Tang, Tao; Peking University, Institute of Medical Technology<br>Zhao, Heng; Peking University, Institute of Medical Technology<br>Liu, Duanke; Peking University, Institute of Medical Technology<br>Li, Xuewei; Peking University, Institute of Medical Technology<br>Wang, Kai; Peking University People's Hospital, Department of Ophthalmology & Clinical Centre of Optometry, Peking University People’s Hospital<br>Li, Yan; Peking University People's Hospital, Department of Ophthalmology & Clinical Centre of Optometry, Peking University People’s Hospital<br>Zhao, Mingwei; Peking University People's Hospital, Department of Ophthalmology & Clinical Centre of Optometry, Peking University People’s Hospital

**Primary Subject Heading**:	Ophthalmology
Secondary Subject Heading:	Paediatrics, Public health
Keywords:	OTOLARYNGOLOGY, Paediatric ophthalmology < OPHTHALMOLOGY, Medical ophthalmology < OPHTHALMOLOGY

SCHOLARONE™
Manuscripts

I, the Submitting Author has the right to grant and does grant on behalf of all authors of the Work (as defined in the below author licence), an exclusive licence and/or a non-exclusive licence for contributions from authors who are: i) UK Crown employees; ii) where BMJ has agreed a CC-BY licence shall apply, and/or iii) in accordance with the terms applicable for US Federal Government officers or employees acting as part of their official duties; on a worldwide, perpetual, irrevocable, royalty-free basis to BMJ Publishing Group Ltd ("BMJ") its licensees and where the relevant Journal is co-owned by BMJ to the co-owners of the Journal, to publish the Work in this journal and any other BMJ products and to exploit all rights, as set out in our licence.

The Submitting Author accepts and understands that any supply made under these terms is made by BMJ to the Submitting Author unless you are acting as an employee on behalf of your employer or a postgraduate student of an affiliated institution which is paying any applicable article publishing charge ("APC") for Open Access articles. Where the Submitting Author wishes to make the Work available on an Open Access basis (and intends to pay the relevant APC), the terms of reuse of such Open Access shall be governed by a Creative Commons licence – details of these licences and which Creative Commons licence will apply to this Work are set out in our licence referred to above.

Other than as permitted in any relevant BMJ Author's Self Archiving Policies, I confirm this Work has not been accepted for publication elsewhere, is not being considered for publication elsewhere and does not duplicate material already published. I confirm all authors consent to publication of this Work and authorise the granting of this licence.

For peer review only

Title Page:

Axial length to corneal radius of curvature ratio and refractive error in 4- to 6-year-old Chinese preschoolers: a retrospective cross-sectional study

Tao Tang[1,2,3.4,5#], Heng Zhao[1,2,3.4,5]

, Duanke Liu[1,2,3.4,5], Xuewei Li[1,2,3.4,5], Kai Wang[1,2,3.4,5*], Yan Li[2,3,4,5*], & Mingwei Zhao[1,2,3.4,5]

[1] Department of Ophthalmology & Clinical Centre of Optometry, Peking University People's Hospital, Beijing, China
[2] Institute of Medical Technology, Peking University Health Science Center, Beijing, China
[3]College of Optometry, Peking University Health Science Center, Beijing, China
[4]Eye Disease and Optometry Institute, Peking University People's Hospital, Beijing, China
[5]Beijing Key Laboratory of the Diagnosis and Therapy of Retinal and Choroid Diseases, Beijing, China

For peer review only

Author Affiliations:

First author: Tao Tang [1,2,3.4,5#]

[1] Department of Ophthalmology & Clinical Centre of Optometry, Peking University People's Hospital, Beijing, China
[2] Institute of Medical Technology, Peking University Health Science Center, Beijing, China
[3]College of Optometry, Peking University Health Science Center, Beijing, China
[4]Eye Disease and Optometry Institute, Peking University People's Hospital, Beijing, China
[5]Beijing Key Laboratory of the Diagnosis and Therapy of Retinal and Choroid Diseases, Beijing, China

Heng Zhao [1, 2, 3, 4, 5]

[1] Department of Ophthalmology & Clinical Centre of Optometry, Peking University People's Hospital, Beijing, China
[2] Institute of Medical Technology, Peking University Health Science Center, Beijing, China
[3]College of Optometry, Peking University Health Science Center, Beijing, China
[4]Eye Disease and Optometry Institute, Peking University People's Hospital, Beijing, China
[5]Beijing Key Laboratory of the Diagnosis and Therapy of Retinal and Choroid Diseases, Beijing, China

Duanke Liu [1, 2, 3, 4, 5]

[1] Department of Ophthalmology & Clinical Centre of Optometry, Peking University People's Hospital, Beijing, China
[2] Institute of Medical Technology, Peking University Health Science Center, Beijing, China
[3]College of Optometry, Peking University Health Science Center, Beijing, China
[4]Eye Disease and Optometry Institute, Peking University People's Hospital, Beijing, China
[5]Beijing Key Laboratory of the Diagnosis and Therapy of Retinal and Choroid Diseases, Beijing, China

Xuewei Li [1, 2, 3, 4, 5]

[1] Department of Ophthalmology & Clinical Centre of Optometry, Peking University People's Hospital, Beijing, China
[2] Institute of Medical Technology, Peking University Health Science Center, Beijing, China
[3]College of Optometry, Peking University Health Science Center, Beijing, China
[4]Eye Disease and Optometry Institute, Peking University People's Hospital, Beijing, China
[5]Beijing Key Laboratory of the Diagnosis and Therapy of Retinal and Choroid Diseases, Beijing, China

Corresponding author: Kai Wang [1, 2, 3, 4, 5*]

For peer review only

[1] Department of Ophthalmology & Clinical Centre of Optometry, Peking University People's Hospital, Beijing, China
[2] Institute of Medical Technology, Peking University Health Science Center, Beijing, China
[3]College of Optometry, Peking University Health Science Center, Beijing, China
[4]Eye Disease and Optometry Institute, Peking University People's Hospital, Beijing, China
[5]Beijing Key Laboratory of the Diagnosis and Therapy of Retinal and Choroid Diseases, Beijing, China

Corresponding author: Yan Li [2,3,4,5 *]
[1]Department of Ophthalmology & Clinical Centre of Optometry, Peking University People's Hospital, Beijing, China
[3]College of Optometry, Peking University Health Science Center, Beijing, China
[4]Eye Disease and Optometry Institute, Peking University People's Hospital, Beijing, China
[5]Beijing Key Laboratory of the Diagnosis and Therapy of Retinal and Choroid Diseases, Beijing, China

Mingwei Zhao [1 , 2 , 3. 4 , 5]
[1] Department of Ophthalmology & Clinical Centre of Optometry, Peking University People's Hospital, Beijing, China
[2] Institute of Medical Technology, Peking University Health Science Center, Beijing, China
[3]College of Optometry, Peking University Health Science Center, Beijing, China
[4]Eye Disease and Optometry Institute, Peking University People's Hospital, Beijing, China
[5]Beijing Key Laboratory of the Diagnosis and Therapy of Retinal and Choroid Diseases, Beijing, China

Short Title: AL/CRC ratio and refractive error in Chinese preschoolers

[*]**Corresponding authors:**
Kai Wang, Department of Ophthalmology & Clinical Centre of Optometry, Peking University People' Hospital; College of Optometry, Peking University Health Science Center. Beijing, China.
E-mail: wang_kai@bjmu.edu.cn **Cell:** 0086-13810097881, **Tel.:** 0086-010-88325413.

For peer review only

Yan Li, Department of Ophthalmology & Clinical Centre of Optometry, Peking University People' Hospital; College of Optometry, Peking University Health Science Center. Beijing, China.

E-mail: 13801153660@163.com **Cell:** 0086-13801153660

Number of Table: two tables.
Number of Figure: two figures.
Number of Supplemental Table: four Supplemental Tables
Number of Supplemental Figure: two Supplemental Figures

For peer review only

ABSTRACT

Objective To investigate the associations of axial length to corneal radius of curvature (AL/CRC) ratio with refractive error and to determine the effect of AL/CRC ratio on hyperopia reserve and myopia assessment among Chinese preschoolers.

Methods This was a retrospective cross-sectional study evaluated subjects aged 4-6 years. AL and CRC were obtained using a noncontact ocular biometer. Correlation analysis was performed to explore the associations of AL/CRC ratio with spherical equivalent refractive error (SER). The accuracy of AL/CRC ratio for hyperopia reserve and myopia assessment were analysed using cycloplegic refraction by drawing receiver operating characteristic (ROC) curves.

Results The analysis included 1024 participants (537 boys, 52.4%). The mean AL/CRC ratios in hyperopes, emmetropes and myopes were 2.90±0.06, 2.95±0.05 and 3.08±0.07, respectively. The SER was found to be more strongly correlated with AL/CRC ratio (ρ=-0.66, p<0.001) than either AL or CRC alone (ρ=-0.52, p<0.001; ρ=-0.03, p=0.33, respectively). AL/CRC was correlated with SER in hyperopes (ρ=-0.54, p<0.001), emmetropes (ρ=-0.33, p<0.001) and myopes (r=-0.67, p<0.001).

For peer review only - http://bmjopen.bmj.com/site/about/guidelines.xhtml

For low hyperopia reserve assessment, the area under the ROC curves of AL/CRC ratio was 0.861 (95% CI: 0.829-0.892), the optimal cut-off value of the AL/CRC ratio was ≥2.955. For myopia assessment, the area under the ROC curves of AL/CRC ratio was 0.954 (95% CI: 0.925-0.982), the optimal cut-off value of the AL/CRC ratio was ≥2.975.

Conclusions The SER showed a better correlation with AL/CRC ratio than either AL or CRC alone, especially in myopes, among 4- to 6-year-old children. These findings indicate that when cycloplegic refraction is unavailable, AL/CRC ratio could be used as an alternative indicator for identifying low hyperopia reserve and myopia among preschoolers, helping clinicians and parents screen children with low hyperopia reserve before primary school in a timely manner.

Strengths and limitations of this study

1. An advantage of this study was the population-based design, with a large sample size of 4- to 6-year-old children.
2. The hyperopic reserve and myopia were assessed by AL/CRC ratio using ROC curves and AUC.
3. This study is cross-sectional and does not pinpoint the temporal correlations between refractive error and the AL/CRC ratio.
4. The sample size of 4-year-old participants was relatively small, which may result in potential for discrepancy.

Key words: myopia, hyperopia reserve, spherical equivalent refractive error, axial length, axial length to corneal radius of curvature ratio

INTRODUCTION

Myopia has become a global public health problem worldwide, especially in East Asian countries such as China, Singapore and South Korea [1-3]. It is predicted that, by 2050, 49.8% of the global population will have myopia and 19.7% will suffer high myopia [4]. It is worth noting that the incidence of myopia has shown a trend toward increasing in those who are younger in recent years. In 2011, the overall prevalence of myopia among children aged 5-14 years in Beijing was 36.7% [5]. In Hong Kong, myopia was 18.3% in children aged 6 years and increased to 61.5% in those aged 12 years in 2018 [6]. In France, the myopic rate was 19.6% in children aged 0 to 9 years in 2013 [7]. For most myopic patients, refractive error tends to stabilize in adulthood, while for a significant proportion of the myopic population, refraction continues to progress in adulthood and develops into pathologic myopia, resulting in various vision-threatening complications such as retinal detachment, glaucoma and cataracts [8-10]. Early monitoring of children's refractive development and timely control of myopia progression will reduce the risk of myopia-related

For peer review only

complications in the future life.

Ocular biometrics including axial length, corneal curvature and lens power are among the most important factors affecting refractive status of the eyes [11]. During infancy and young childhood, most children have a physiological hyperopia of approximately +2.00 diopters (D), which is defined as hyperopia reserve [12,13]. Balancing changes in axial length and ocular refractive components, including the cornea and the lens, leads to emmetropization [14]. As children grow older, the refraction of the eye develops, and the hyperopia reserve will gradually decrease. Myopia is an ocular disorder characterized by excessive ocular elongation in relation to total ocular power. It has been widely accepted that the myopia shift in refraction in schoolchildren is due to axial elongation [15,16]. When the rate of axial elongation outpaces the changes in optical power of the cornea and the lens, there is a tendency towards myopia onset [17]. For young children, an early reduction in hyperopic reserve might be a risk factor for future myopia development [18]. Zadnik et al. found that children in the United States in grade 1 with less than +0.75 D of hyperopia are at increased risk of myopia formation [18]. Yue et al. also reported that the cut point for myopia for grade 1 students was +0.31 D in the Chinese population [19]. Myopia develops fastest at the age of 6-7 years, and tends to slow down after 11 years [20]. Thus, to prevent the formation of myopia at an early age, clinicians and parents should pay attention to children with low hyperopia reserve and begin to monitor the development of refractive error before primary school.

Typically, cycloplegic refraction is the gold standard for paediatric refractive error diagnosis [21-23], especially in younger children [22,23], largely due to an active accommodation response. However, considering that the side effects of cycloplegia such as allergic conjunctivitis, photophobia and poor near visual acuity [24-26], cause parents or children to be reluctant to undergo cycloplegic refraction. Furthermore, for clinicians who lack regulatory approval for the use of cycloplegics and some patients who are unable to use cycloplegia, cycloplegic refraction may not be performed in such situations. Effective strategies are needed to examine ocular refractive development and diagnose refractive error in young children. Scheiman M et al. reported that the axial length to corneal radius of curvature (AL/CRC) ratio could be a better marker of myopia progression than AL alone [27]. The correlation between refraction and the AL/CRC ratio was stronger than that between refraction and AL [27-29], and the AL/CRC ratio could explain the total variance in refractive error better than AL alone [30,31]. Moreover, the measurement of AL and CRC is noninvasive, and it is easily accepted by children [29]. Thus, although cycloplegic refraction cannot be replaced by any other measurements for the diagnosis of refractive error in children, as an alternative, the AL/CRC ratio can be used as an approximated surrogate for refractive error estimation.

Although many studies have addressed the correlation between the AL/CRC ratio and refractive error in school-aged children and adult population [17,32-35], few population-based studies to date have been performed to focus on the correlation between the AL/CRC ratio and refractive error and evaluate the effect of the AL/CRC ratio for hyperopia reserve and myopia assessment among preschool children in China. Therefore, in this study, we aimed to report the AL/CRC ratio in a Chinese preschool student population. In addition, we examined the correlation between the AL/CRC ratio and refractive error and evaluated the effectiveness of the AL/CRC ratio for refractive error assessment in 4- to 6-year-old Chinese preschoolers.

For peer review only - http://bmjopen.bmj.com/site/about/guidelines.xhtml

METHODS

Setting and participants

The study consisting of participants who visited the Peking University People's Hospital optometry centre between January 2020 and September 2022 due to refractive error, was conducted to investigate AL/CRC ratio and refractive error. All the subjects underwent a comprehensive ophthalmological examination and 1024 subjects fulfilled the following inclusion criteria were enrolled in the retrospective cross-sectional study: (1) age of 4 to 6 years old; (2) astigmatism and anisometropia of 1.50 D or less; (3) the monocular best corrected visual acuity (VA) of 0.00 logMAR (6/6) or better; (4) the absence of any ocular and systemic diseases, such as cataracts, glaucoma, and strabismus; (5) cooperate in completing eye examinations. This study was approved by the institutional research ethics committee of Peking University People's Hospital (2021PHB322-001). All examinations and management were performed in accordance with the Declaration of Helsinki, and written informed consent was obtained from the parents.

Measures

Refraction and ocular biometry examinations were performed for all subjects. First, measurement of ocular biometric parameters was performed with an optical biometer. AL was measured with noncontact partial-coherence laser interferometry (IOL Master; Carl Zeiss Meditec, Oberkochen, Germany). A corneal topography system (the Sirius, Italy) was used to obtain the mean K reading (Kmean) and anterior chamber depth (ACD). Five valid readings were taken and averaged. The measurements were taken by the same experienced technical staff, and the mean value of these measurements was taken for statistical analysis. The ocular parameters presented here are the mean ± standard deviation (SD).

After the biometry measurements, all subjects underwent noncycloplegic refraction with an autorefractor (KP8800; Topcon Corp., Tokyo, Japan). This instrument generated five reliable readings of refraction in both eyes; the median reading was used for analysis. Then, cycloplegic refraction was performed for each subject with three drops of 1% cyclopentolate, the first two drops were administered 5 minutes apart, and the third drop was administered 20 minutes later. Cycloplegia was considered complete if the pupil dilated to 6 mm or more and there was no pupillary reflex. The average of five readings automatically performed by the autorefractor was used for analyses. Slit-lamp examination and fundus examination were performed.

Definitions

Refraction was defined as spherical equivalent refraction (SER; SER = spherical power + cylinder power/2). Myopia was defined as SER≤-0.50 D, emmetropia as –0.50 D<SER≤+0.50 D and hyperopia as SER>+0.50 D. Signals from the tear film and retinal pigment epithelium (RPE) are used by noncontact partial-coherence laser interferometry (IOL Master; Carl Zeiss Meditec, Oberkochen, Germany) for axial length measurements. Anterior chamber depth was defined as the distance from the anterior corneal surface to the anterior lens surface. The mean K reading (Kmean = (flat K reading + steep K reading)/2) was the average of the steepest and flattest

For peer review only

meridians. The corneal radius of curvature (CRC) was converted from the Kmean data using the formula CRC=0.3375/Kmean (D)×1000. The axial length to corneal radius of curvature ratio (AL/CRC ratio) was defined as the axial length divided by the mean corneal radius of curvature. Lens power was calculated using the Bennett-Rabbetts method [36] with unknown lens thickness, using measured values for SER, ACD, CRC and AL. The equation was as follows by the formula of Bennett.

$$LP = \frac{L(S_{cv} + K) - 1000n}{(L - ACD - c_{BR})\left(\frac{ACD + c_{BR}}{1000n}(S_{CV} + K) - 1\right)}$$

In this formula, LP is the lens power using the Bennett-Rabbetts method, L is the axial length, S is the spherical equivalent refraction at the corneal vertex, S_{CV}=S/(1-0.014S), K is the mean corneal power, n=1.336, and c_{BR}=2.891±0.778 mm.

Analysis

Statistical analysis was performed using the SPSS statistical software package (Version 22.0, IBM Corp., US). Descriptive statistics were calculated. Continuous data are expressed as the mean ± standard deviation (mean±SD). As biometric data for the right and left eyes were highly correlated (Pearson correlation coefficient, r=0.91), analyses were performed using data for the right eye only. The AL, CRC, AL/CRC ratio, and SER were analysed as continuous variables, and the Kolmogorov–Smirnov test was performed to verify the normality of these variables. To show the correlations between SER and AL, CRC, and the AL/CRC ratio, Spearman's correlation analyses were performed. The relationship between the AL/CRC ratio and other variables was assessed using Spearman's correlation. SER, AL, CRC, AL/CRC ratio, ACD and LP were compared between groups using independent sample one-way ANOVA. Separate linear regression models were constructed with SER as the dependent variable and AL/CRC ratio, AL, and CRC as the main covariate, respectively. The sensitivity, specificity, Youden index, true positive rate, and false positive rate of the AL/CRC ratio and AL for hyperopia reserve and myopia assessment were analysed. The effectiveness of the AL/CR ratio, CRC and AL for hyperopia reserve and myopia assessment was evaluated using receiver operating characteristic (ROC) curves and the area under the curve (AUC) of the ROC curves. All p values were two sided, and a p value of < 0.05 was indicated statistical significance.

Patient and Public Involvement

None.

For peer review only

RESULTS

Demographic characteristics

A total of 1024 children (537 boys and 487 girls) aged 4-6 years (mean 5.77±0.47 years) were included in this study. The average ages of the boys and girls were 5.79 and 5.76 years, respectively. There was no significant difference in sex distribution ($\chi^2 = 2.441$, p=0.118). The mean SER was 0.90±1.11 D, mean AL was 22.59±0.76 mm, mean CRC was 7.74±0.24 mm and mean AL/CR ratio was 2.92±0.08. The detailed demographic characteristics of the subjects are shown in **Supplemental Table 1**.

The histograms of SER, AL, CRC and AL/CRC ratio are shown in **Supplemental Figure 1**. The Kolmogorov–Smirnov test indicated that the frequency distribution was nonnormal for SER, AL, CRC and the AL/CRC ratio (all p<0.05). The overall prevalence rates of myopia and high myopia were 7.3% and 0.1%, respectively. The prevalence rates of myopia in subjects aged 4, 5, and 6 years were 4.2% (1/24), 5.4% (10/186), and 7.9% (64/814), respectively.

Table 1 shows the distribution of ocular biometry and the AL/CRC ratio according to the categories of refractive status of subjects. Axial length was significantly longer (p<0.001) in myopes (23.74 ± 0.65 mm) than in emmetropes (22.86 ± 0.63 mm) and hyperopes (22.42 ± 0.67 mm) (p<0.001). The AL/CRC ratio was also significantly higher in myopes (3.08±0.07) than in emmetropes (2.95±0.05) and hyperopes (2.90±0.06) (p<0.001), while CRC and ACD tended to stabilize in different refractive statuses. Emmetropes have the largest LP, while myopes have the smallest LP. **Supplemental Figure 2** shows that AL/CRC ratio correlates with age, and the older the age, the greater the value of AL/CRC ratio.

Table 1. Values of ocular parameters and correlations between AL, CRC, AL/CRC ratio and SER according to refractive status

Parameters	Hyperopes (n=772)	Emmetropes (n=174)	Myopes (n=78)	p value
SER	+1.32±0.73	+0.18±0.30	-1.70±1.05	<0.001

For peer review only

AL	22.42±0.67	22.86±0.63	23.74±0.65	<0.001
CRC	7.74±0.24	7.76±0.26	7.72±0.21	0.678
AL/CRC	2.90±0.06	2.95±0.05	3.08±0.07	<0.001
ACD	3.35±0.27	3.46±0.23	3.30±0.26	0.140
LP	24.18±1.27	24.31±1.32	22.62±1.28	<0.001
Spearman's correlation coefficients				
SER and AL	-0.41*	-0.08	-0.37*	
SER and CRC	-0.06	0.10	-0.15	
SER and AL/CRC	-0.54*	-0.33*	-0.67*	

SER, spherical equivalent refractive error; AL, axial length; CRC, corneal radius of curvature; AL/CRC ratio, axial length to corneal radius of curvature ratio; LP, lens power; ACD, anterior chamber depth; D, diopter. Significant differences are masked by asterisks.

Correlation analysis between AL, CRC, AL/CRC ratio and SER

Figure 1 shows the scatter plots between AL, CRC, AL/CRC ratio and SER. The SER showed a better correlation with the AL/CRC ratio (Spearman's correlation coefficient, ρ=-0.66, p<0.001) than either AL (ρ=-0.52, p<0.001) or CRC (ρ=-0.03, p=0.33) (**Supplemental Table 2**). The Spearman's correlation coefficient between SER and the AL/CRC ratio was the highest in myopes (ρ=-0.67, p<0.001), followed by hyperopes (ρ=-0.54, p<0.001) and emmetropes (ρ=-0.33, p<0.001). There was a significant correlation between SER and AL in hyperopes (ρ=-0.41, p<0.001) and myopes (ρ=-0.37, p<0.001), but not in emmetropes (ρ=-0.08, p=0.28). There was

For peer review only

no statistical significance for the correlation between SER and CRC in all refractive groups (all p>0.05). Compared to either AL or CRC, the AL/CRC ratio showed better correlation with SER in all refractive groups (**Table 1**).

Regression analysis between AL, CRC, AL/CRC ratio and SER

Univariate and multiple linear regression analyses were performed in this study (**Supplemental Table 2**). For SER variance, AL explained 34.5%, whereas the AL/CRC ratio explained 64.5% of the data after adjusting for age and sex. Multiple linear regression adjusted for age and sex showed that a 0.1 increase in the AL/CRC ratio was associated with a -1.19 D change in SER and a 1 mm increase in AL was associated with a -0.87 D change in SER.

Effectiveness of AL, CRC and the AL/CR ratio for hyperopia reserve assessment and myopia assessment

Taking cycloplegic refraction SER ≤ +0.50 D as the standard for diagnosis of the lack of hyperopia reserve [37], the effectiveness of AL, CRC and AL/CR ratio for myopia assessment were analysed (**Figure 2A**). The ROC curves were drawn using AL, CRC and the AL/CR ratio as the index for hyperopia reserve assessment, and the AUCs of the ROC curves were 0.777 (95% confidence interval, CI: 0.741-0.812), 0.494(95% CI: 0.450-0.539) and 0.861 (95% CI: 0.829-0.892), respectively. If AL was used for hyperopia reserve assessment, the optimal cut-off point of the ROC curve was ≥22.615 mm, with a specificity, sensitivity, Youden index, true positive rate, and false positive rate of 0.785, 0.604, 0.389, 0.604 and 0.215, respectively. If the AL/CR ratio was used for myopia assessment, the optimal cut-off point of the ROC curve was ≥2.955, with a specificity, sensitivity, Youden index, true positive rate, and false positive rate of 0.732, 0.863, 0.595, 0.863, and 0.268, respectively (**Table 2**). When optimal cut-off value of AL/CRC ratio was used to assess the lack of hyperopic reserve, 196 of the 252 participants with low hyperopic reserve were correctly classified using the optimal AL/CRC threshold. Using the optimal cut-off value of AL/CRC ratio, 22.22% (56/252) of participants with a lack of hyperopia reserve were not correctly classified (**Supplemental Table 3**).

Table 2. The sensitivity, specificity, and Youden index of AL and the AL/CRC ratio for hyperopia reserve assessment and myopia assessment.

	SER	Criterion	Cut-off value	Sensitivity	Specificity	Youden index
Hyperopia reserve assessment	≤ +0.50 D	AL (mm)	22.615	0.785	0.604	0.389
		AL/CRC ratio	2.955	0.732	0.863	0.595
	≤ +1.00 D	AL (mm)	22.515	0.677	0.693	0.370
		AL/CRC ratio	2.925	0.670	0.774	0.444
	≤ +1.50 D	AL (mm)	22.495	0.625	0.778	0.403

For peer review only

		AL/CRC ratio	2.895	0.736	0.736	0.472
Myopia assessment	≤ -0.50 D	AL (mm)	23.235	0.800	0.862	0.662
		AL/CRC ratio	2.975	0.894	0.883	0.777

AL, axial length; AL/CRC ratio, axial length to corneal radius of curvature ratio.

Taking cycloplegic refraction SER ≤ −0.50 D as the gold standard for diagnosis of myopia [19], the effectiveness of AL, CRC and AL/CR ratio for myopia assessment were analysed (**Figure 2B**). The ROC curves were drawn using AL, CRC and the AL/CR ratio as the index for myopia assessment, and the AUCs of the ROC curves were 0.898 (95% CI: 0.864-0.932), 0.493(95% CI: 0.434-0.553) and 0.954 (95% CI: 0.925-0.982), respectively. If AL was used for myopia assessment, the optimal cut-off point of the ROC curve was ≥23.235 mm, with a specificity, sensitivity, Youden index, true positive rate, and false positive rate of 0.862, 0.800, 0.662, 0.800 and 0.138, respectively. If the AL/CR ratio was used for myopia assessment, the optimal cut-off point of the ROC curve was ≥2.975, with a specificity, sensitivity, Youden index, true positive rate, and false positive rate of 0.883, 0.894, 0.777, 0.894, and 0.117, respectively (**Table 2**). When optimal cut-off value of AL/CRC ratio was used to assess true myopia, 79 of the 85 participants with myopia were correctly classified using the optimal AL/CRC threshold. Using the optimal cut-off value of AL/CRC ratio, 7.06% (6/85) of participants with myopia were not correctly classified (**Supplemental Table 3**).

DISCUSSION

This study found that AL/CRC ratio was correlated with SER in hyperopes, emmetropes and myopes, while AL showed correlation with SER in hyperopes and myopes, but not in emmetropes. The SER showed a better correlation with the AL/CRC ratio than either AL or CRC alone in different refractive statuses, especially in myopes, among 4- to 6-year-old children. The effect of the AL/CR ratio for low hyperopia reserve and myopia assessment was higher than that of either AL or CRC alone. These findings indicate that when cycloplegic refraction is unavailable, AL/CRC ratio can be used as an alternative indicator for monitoring low hyperopia reserve and myopia among preschoolers.

Myopia is a common disorder of the eyes and has become a global public health concern [38,39]. During the past several decades, the prevalence of myopia in children has increased rapidly, and the age at onset of myopia has decreased. Chinese children tend to have a higher prevalence of myopia than children in other countries and of other ethnicities. The prevalence of myopia exceeds 60% among 12 years of age in China after primary school, reaches nearly 80% at 16 years of age after junior high school, and surpasses 90% in university students [1,4,13]. Notably, the incidence of myopia increased among preschool children in recent years. The prevalence of myopia was 17.6% in children aged 6 years in Hong Kong [40]. A study in Shanghai of 7166 Chinese children aged 4 to 6 years reported that the

For peer review only

prevalence of myopia ranged from 5.7% to 7.1% [41]. In this study, the prevalence of myopia in preschoolers increased from 4.2% at 4 years of age to 7.9% at 6 years of age. Although myopia tends to stabilize after adulthood, children with a younger age at myopia onset are prone to develop high myopia and have a high risk of suffering from vision-threatening complications. In view of this condition, it is urgent to carry out early vision screening and refractive error assessment among preschool children. Ocular biometrics have been used to estimate the progression of refractive error [29,34]. The AL/CRC ratio summarizes the overall relationship between AL and CRC and minimizes variability in AL and CRC, thus, it has been considered as a more robust index to evaluate the refractive status of the eye [17]. Moreover, the AL/CRC ratio also correlates with refractive error more strongly than AL or CRC alone [28,31]. Therefore, the AL/CRC ratio could be useful in determining refractive error and monitoring myopia progression.

Changes in AL and CRC are important ocular biometrics affecting refractive status [34]. Previous studies have indicated that CRC may be actively modulated to regulate refractive development, and the onset of myopia is due to a failure of corneal compensation for axial elongation [11,33]. In this study, we found that there was no correlation between CRC and refractive error, while AL, or more strikingly, the AL/CRC ratio had a significant correlation with refractive error. This finding suggests that AL has a larger effect on refractive error than CRC. Grosvenor et al. was the first to report the correlation between the AL/CRC ratio and refractive error and studied the role of the AL/CRC ratio in determining the refractive state of the eye [42]. They demonstrated that the AL/CRC ratio was the most important biometric factor in myopia. Some researchers further confirmed Grosvenor's findings [29,43,44]. The distribution of AL/CRC ratio has been reported in different countries and age populations. A summary of AL/CRC ratio in other studies is shown in **Supplemental Table 4**. The average values reported for the AL/CRC ratio range from 2.81 to 3.13. On the one hand, differences in age, race, included subjects, measurement methods and techniques may account for the disagreement in the various studies. On the other hand, we can find that the older age, the greater value of AL/CRC ratio. **Supplemental Figure 2** also confirms this result. This finding indicates that, in addition to refraction, age also affects the value of AL/CRC ratio. The AL/CRC ratio has been proposed as a calculated biometric parameter that correlates better with SER than AL, and the AL/CRC ratio could explain the total variance in SER better than AL alone [31]. This is because the AL/CRC ratio provides a rough measure of the degree of matching between AL and corneal power. In this study, we also found a better correlation between the AL/CRC ratio and SER in children aged 4-6 years compared to that of AL or CRC alone, especially in children with myopia. It is possibly suggested that the AL/CRC ratio is a more useful marker of progress towards myopic refractions than absolute ocular biometric values such as AL or CRC. Moreover, the value of the AL/CRC ratio was negatively correlated with SER. As demonstrated in this study, the AL/CRC ratio was significantly higher in myopes than in emmetropes and hyperopes, which was consistent with studies performed by Foo et al. and Guo et al. [28,31]. This finding indicates that the greater the myopic refraction, the larger the AL/CRC ratio.

A previous study reported that a high AL/CRC ratio of no less than 3.0 has been considered an indicator for the onset of myopia [42]. High axial length to corneal radius ratio is a risk factor in the development of myopia. When the AL/CRC ratio was higher than 3.0, it was considered that other ocular parameters were not able to compensate for the change in refraction caused by the elongation of AL, thereby inducing myopia. As shown in this study, the area under the ROC of the AL/CR ratio

For peer review only - http://bmjopen.bmj.com/site/about/guidelines.xhtml

for myopia assessment was 0.954, and the optimal cut-off value is ≥2.975, which was smaller than that in the study performed by Mu et al. [29]. This result indicates that for children aged 4 to 6 years, the risk of myopia is likely to occur when the AL/CRC ratio is greater than 2.975. However, it is worth noting that this AL/CRC threshold is still subject to some degree of error assessment in the evaluation of true myopia. We also conducted an evaluation of the effectiveness of the AL/CRC ratio, CRC and AL for hyperopia reserve assessment in this population. Previous study has reported that hyperopia reserve is a potential indicator of myopia [19]. There is a progressive shift in mean refraction from roughly + 2.00 D at 3 months of age to approximately + 1.00 D at 6.5 years of age [45]. Then emmetropization continues at a slower rate after 6 years of age. Zadnik et al. revealed that children in the United States in grade 1 with less than +0.75 D of hyperopia are at increased risk of myopia onset [18]. Yue et al. also reported that the cut point for myopia for grade 1 students was +0.31 D in the Chinese population [19]. Li et al.'s [41] findings showed that the 5-year cumulative incidence of myopia was 4.6%, 26.3%, 52.3%, 78.6%, 92.6% and 94.3%, respectively, corresponding to students with a baseline hyperopia reserve of >+2.00 D, +1.50 D to +2.00 D, +1.00 D to +1.50 D, +0.50 D to +1.00 D, 0.00 D to +0.50 D and -0.50 D to 0.00 D, respectively. The lower the hyperopia reserve in young children, the higher the incidence of myopia onset. In our study, taking cycloplegic refraction SER ≤ +0.50 D as the standard for diagnosis of the lack of hyperopia reserve, the area under the ROC curve of the AL/CR ratio for the lack of hyperopia reserve assessment was greater than that of AL and CRC and the optimal cut-off value of the AL/CRC ratio was ≥2.955. Although this optimal AL/CRC threshold may have error assessment for preschoolers, clinicians and parents could monitor the hyperopia reserve of young children by regularly measuring the axial length and corneal curvature when cycloplegic refraction is unavailable. When the AL/CRC ratio is greater than 2.955 among preschool children, clinicians and parents should pay attention to the children with less hyperopia reserve and begin to take measures to prevent myopia formation.

In the present study, AL alone contributed 34.5% of the variance in SER, whereas the AL/CR ratio accounted for 64.5%. Guo et al. study [31] found that AL alone contributed 18.6% of the variance in SER, whereas the AL/CR ratio accounted for 39.8% in the study population of preschoolers aged 3-6 years old. He et al. [30] reported that AL/CR ratio and AL explained 66.4% and 43.1% of the variance in SER, respectively, in a group of Chinese children 6 to 12 years of age. This suggested that the AL/CRC ratio is more closely related to refractive error than AL or CRC alone. Moreover, we found a linear increase in AL/CRC ratio from hyperopia towards myopia. The corresponding increase in SER of -1.1 9D with every 0.1 increase in the AL/CRC ratio in this study is larger than -0.74 D reported by researchers in Singapore [28] and -0.89D reported among the Nigerian population [33], but slightly lower than -1.21 D reported among northern Iranian subjects [17]. The above findings revealed that in addition to AL and CRC, there are other potential factors that influence SER results. Thus, the AL/CRC ratio cannot completely replace cycloplegic refraction in determining refraction in children. However, cycloplegic refraction is easily influenced by the cooperation and compliance of the examinees, especially in younger children, which is not feasible for large-scale myopia assessment and long-term refractive development follow-up. For this reason, the AL/CRC ratio could be used as an alternative indicator for refraction estimation, and it may be more accurate than AL. In addition, measurement of AL and CRC and determination of the AL/CRC ratio are relatively easy and can be conducted by a technician using noncontact, objective, and automated instruments. The application

For peer review only

of the AL/CRC ratio reference as an indicator of refractive errors has the effect of simplifying the examination procedure and reducing unnecessary cycloplegia, especially when cycloplegic refraction is difficult to perform in 4- to 6-year-old children.

There were some limitations in this study. First, because this study is cross-sectional and does not pinpoint the temporal correlations between refractive error and the AL/CRC ratio, it is impossible to rule out the possibility of observational and inclusionary biases due to the retrospective study design. Admittedly, longitudinal studies would be needed to conduct to evaluate intraindividual discrepancy. In addition, the data from Beijing may not be representative for the rural areas and other regions in China. Last, the sample size of the 4-year-old participants (N=24) was relatively small. Although our findings are applicable to this population as well, there is potential for discrepancy. We will further increase the sample size of this population to increase the accuracy and reliability of the results of this study.

In conclusion, our study found that SER showed a better correlation with the AL/CRC ratio than either AL or CRC alone in different refractive statuses, especially in myopic children, among 4- to 6-year-old Chinese children. The AL/CRC ratio could explain the total variance in refractive error better than AL alone. The effect of the AL/CR ratio for assessing the lack of hyperopia reserve and for the myopia assessment was higher than that of AL and CRC. These findings indicate that when cycloplegic refraction is unavailable, the AL/CRC ratio could be used as an alternative indicator for identifying low hyperopia reserve and myopia among preschoolers, helping clinicians and parents screen children with low hyperopia reserve before primary school in a timely manner. In the future, longitudinal cohort designs will be conducted for optimal study of the effect of the AL/CRC ratio on hyperopia reserve and myopia assessment in preschool children.

Declarations

Authors' contributions

TT developed the concept, collected data, performed the experiments and data analysis, wrote the main manuscript text and prepared Figures 1-2, Tables 1-2, Supplemental Figures 1-2, Supplemental Tables 1-4; HZ collected data and prepared Figure 2; DL and XL collected data; KW and YL developed the concept and designed the experiments; MZ developed the study conception. TT wrote the first draft of the manuscript. TT, KW and YL made critical revisions. All authors reviewed and approved the final draft. TT is the guarantor and responsible for the overall content.

Funding

This work was supported by: National Natural Science Foundation of China (Grant Nos. 82371087 and 82171092), Capital’s Funds for Health Improvement and Research (No. 2022-1G-4083), the National Key R&D Program of China (No.2020YFC2008200 and No.2021YFC2702100).

For peer review only

Competing interests

The authors declare that they have no conflicts of interest.

Patient consent for publication

Not applicable.

Ethics approval

This study was approved by the institutional research ethics committee of Peking University People's Hospital (2021PHB322-001). All examinations and management were performed in accordance with the Declaration of Helsinki, and written informed consent was obtained from the parents.

Availability of data and materials

All data generated or analysed during this study are included in this published article.

Acknowledgements

None.

Corresponding author

Correspondence to Kai Wang and Yan Li

Data Availability Statement

Data are available upon reasonable request.

References

1. Ma Y, Qu X, Zhu X, et al. Age-Specific Prevalence of Visual Impairment and Refractive Error in Children Aged 3-10 Years in Shanghai, China. Invest Ophthalmol Vis Sci. 2016;57(14):6188-6196.
2. Koh V, Yang A, Saw SM, et al. Differences in Prevalence of Refractive Errors in Young Asian Males in Singapore between 1996–1997 and 2009–2010.

For peer review only

Ophthalmic Epidemiology. 2014;21(4):247-255.

3. Jung SK, Lee JH, Kakizaki H, Jee D. Prevalence of myopia and its association with body stature and educational level in 19-year-old male conscripts in seoul, South Korea. Invest Ophthalmol Vis Sci. 2012 Aug 15;53(9):5579-83.

4. Holden BA, Fricke TR, Wilson DA, et al. Global Prevalence of Myopia and High Myopia and Temporal Trends from 2000 through 2050. Ophthalmology. 2016;123(5):1036-1042.

5. Lyu Y, Zhang H, Gong Y, et al. Prevalence of and factors associated with myopia in primary school students in the Chaoyang District of Beijing, China. Jpn J Ophthalmol. 2015;59(6):421-429.

6. Choy BNK, You Q, Zhu MM, Lai JSM, Ng ALK, Wong IYH. Prevalence and associations of myopia in Hong Kong primary school students. Jpn J Ophthalmol. 2020;64(4):437-449.

7. Matamoros E, Ingrand P, Pelen F, et al. Prevalence of Myopia in France: A Cross-Sectional Analysis. Medicine (Baltimore). 2015;94(45):e1976.

8. Lee JY, Sung KR, Han S, Na JH. Effect of myopia on the progression of primary open-angle glaucoma. Invest Ophthalmol Vis Sci. 2015;56(3):1775-1781.

9. Ohno-Matsui K, Wu PC, Yamashiro K, et al. IMI Pathologic Myopia. Invest Ophthalmol Vis Sci. 2021;62(5):5.

10. Haarman AEG, Enthoven CA, Tideman JWL, Tedja MS, Verhoeven VJM, Klaver CCW. The Complications of Myopia: A Review and Meta-Analysis. Invest Ophthalmol Vis Sci. 2020;61(4):49.

11. Mutti DO, Mitchell GL, Jones LA, et al. Axial growth and changes in lenticular and corneal power during emmetropization in infants. Invest Ophthalmol Vis Sci. 2005;46(9):3074-3080.

12. Flitcroft DI. Emmetropisation and the aetiology of refractive errors. Eye (Lond). 2014;28(2):169-179.

13. Song Y, Zhu S, Yang B, et al. Accommodation and binocular vision changes after wearing orthokeratology lens in 8- to 14-year-old myopic children. Graefes Arch Clin Exp Ophthalmol. 2021;259(7):2035-2045.

14. Rozema J, Dankert S, Iribarren R. Emmetropization and nonmyopic eye growth. Surv Ophthalmol. 2023; 68(4):759-783.

15. Ojaimi E, Rose KA, Morgan IG, et al. Distribution of ocular biometric parameters and refraction in a population-based study of Australian children. Invest Ophthalmol Vis Sci. 2005;46(8):2748-2754.

16. Chamberlain P, Lazon de la Jara P, Arumugam B, Bullimore MA. Axial length targets for myopia control. Ophthalmic Physiol Opt. 2021 May;41(3):523-531.

17. Hashemi H, Khabazkhoob M, Miraftab M, Emamian MH, Shariati M, Abdolahi-Nia T, et al. Axial length to corneal radius of curvature ratio and refractive errors. J Ophthalmic Vis Res. 2013 Jul;8(3):220-6.

18. Zadnik K, Sinnott LT, Cotter SA, et al. Prediction of Juvenile-Onset Myopia. JAMA Ophthalmol. 2015;133(6):683-689.

For peer review only

19. Yue Y, Liu X, Yi S, Liu B, Yi H, Li H. High prevalence of myopia and low hyperopia reserve in 4411 Chinese primary school students and associated risk factors. BMC Ophthalmol. 2022;22(1):212.

20. Hyman L, Gwiazda J, Hussein M, Norton TT, Wang Y, Marsh-Tootle W, et al. Relationship of age, sex, and ethnicity with myopia progression and axial elongation in the correction of myopia evaluation trial. Arch Ophthalmol. 2005 Jul;123(7):977-87.

21. Morgan IG, Iribarren R, Fotouhi A, Grzybowski A. Cycloplegic refraction is the gold standard for epidemiological studies. Acta Ophthalmol. 2015;93(6):581-585.

22. Li T, Zhou X, Zhu J, Tang X, Gu X. Effect of cycloplegia on the measurement of refractive error in Chinese children. Clin Exp Optom. 2019;102(2):160-165.

23. Hu YY, Wu JF, Lu TL, et al. Effect of cycloplegia on the refractive status of children: the Shandong children eye study. PLoS One. 2015;10(2):e0117482.

24. Chia A, Chua WH, Cheung YB, et al. Atropine for the treatment of childhood myopia: safety and efficacy of 0.5%, 0.1%, and 0.01% doses (Atropine for the Treatment of Myopia 2). Ophthalmology. 2012;119(2):347-354.

25. Gong Q, Janowski M, Luo M, et al. Efficacy and Adverse Effects of Atropine in Childhood Myopia: A Meta-analysis. JAMA Ophthalmol. 2017;135(6):624-630.

26. Wei S, Li SM, An W, et al. Safety and Efficacy of Low-Dose Atropine Eyedrops for the Treatment of Myopia Progression in Chinese Children: A Randomized Clinical Trial. JAMA Ophthalmol. 2020;138(11):1178-1184.

27. Scheiman M, Gwiazda J, Zhang Q, et al. Longitudinal changes in corneal curvature and its relationship to axial length in the Correction of Myopia Evaluation Trial (COMET) cohort. J Optom. 2016;9(1):13-21.

28. Foo VH, Verkicharla PK, Ikram MK, Chua SY, Cai S, Tan CS, et al. Axial Length/Corneal Radius of Curvature Ratio and Myopia in 3-Year-Old Children. Transl Vis Sci Technol. 2016;5(1):5.

29. Mu J, Zeng D, Fan J, et al. The accuracy of the axial length and axial length/corneal radius ratio for myopia assessment among Chinese children. Front Pediatr. 2022;10:859944.

30. He X, Zou H, Lu L, et al. Axial length/corneal radius ratio: association with refractive state and role on myopia detection combined with visual acuity in Chinese schoolchildren. PLoS One. 2015;10(2):e0111766.

31. Guo X, Fu M, Ding X, Morgan IG, Zeng Y, He M. Significant Axial Elongation with Minimal Change in Refraction in 3- to 6-Year-Old Chinese Preschoolers: The Shenzhen Kindergarten Eye Study. Ophthalmology. 2017;124(12):1826-1838.

32. Jong M, Sankaridurg P, Naduvilath TJ, Li W, He M. The Relationship between Progression in Axial Length/Corneal Radius of Curvature Ratio and Spherical Equivalent Refractive Error in Myopia. Optom Vis Sci. 2018;95(10):921-929.

For peer review only

33. Badmus SA, Ajaiyeoba AI, Adegbehingbe BO, Onakpoya OH, Adeoye AO. Axial length/corneal radius of curvature ratio and refractive status in an adult Nigerian population. Niger J Clin Pract. 2017;20(10):1328-1334.

34. Iyamu E, Iyamu J, Obiakor CI. The role of axial length-corneal radius of curvature ratio in refractive state categorization in a nigerian population. ISRN Ophthalmol. 2011;2011:138941.

35. Warrier S, Wu HM, Newland HS, et al. Ocular biometry and determinants of refractive error in rural Myanmar: the Meiktila Eye Study. Br J Ophthalmol. 2008;92(12):1591-1594.

36. Rozema JJ, Atchison DA, Tassignon MJ. Comparing methods to estimate the human lens power. Invest Ophthalmol Vis Sci. 2011;52(11):7937-7942.

37. Li SM, Wei S, Atchison DA, et al. Annual Incidences and Progressions of Myopia and High Myopia in Chinese Schoolchildren Based on a 5-Year Cohort Study. Invest Ophthalmol Vis Sci. 2022;63(1):8.

38. Foster PJ, Jiang Y. Epidemiology of myopia. Eye (Lond). 2014;28(2):202-208.

39. Dolgin E. The myopia boom. Nature. 2015;519(7543):276-8.

40. Lam CS, Lam CH, Cheng SC, Chan LY. Prevalence of myopia among Hong Kong Chinese schoolchildren: changes over two decades. Ophthalmic Physiol Opt. 2012;32(1):17-24.

41. Li T, Zhou X, Chen X, Qi H, Gao Q. Refractive Error in Chinese Preschool Children: The Shanghai Study. Eye Contact Lens. 2019;45(3):182-187.

42. Grosvenor T. High axial length/corneal radius ratio as a risk factor in the development of myopia. Am J Optom Physiol Opt. 1988 Sep;65(9):689-96.

43. Matsumura S, Dannoue K, Kawakami M, et al. Prevalence of Myopia and Its Associated Factors Among Japanese Preschool Children. Front Public Health. 2022;10:901480.

44. Liu S, Chen J, Wang J, Zhu Z, Zhang J, Zhang B, Yang J, Du L, Zhu J, Zou H, He X, Xu X. Cutoff values of axial length/corneal radius ratio for determining myopia vary with age among 3-18 years old children and adolescents. Graefes Arch Clin Exp Ophthalmol. 2023.

45. Mutti DO, Sinnott LT, Lynn Mitchell G, et al. Ocular Component Development during Infancy and Early Childhood. Optom Vis Sci. 2018;95(11):976-985.

Legends:

Figure 1. Correlation analysis between AL, CRC, AL/CRC ratio and SER. SER, spherical equivalent refractive error; AL, axial length; CRC, corneal radius of curvature; AL/CRC ratio, axial length to corneal radius of curvature ratio, D, diopter.

Figure 2. Comparison of the effectiveness of AL, CRC and the AL/CR ratio for hyperopia reserve assessment (A) and myopia assessment (B). AL, axial length; CRC, corneal radius of curvature; AL/CRC ratio, axial length to corneal radius of curvature ratio; AUC, area under curve.

For peer review only

For peer review only

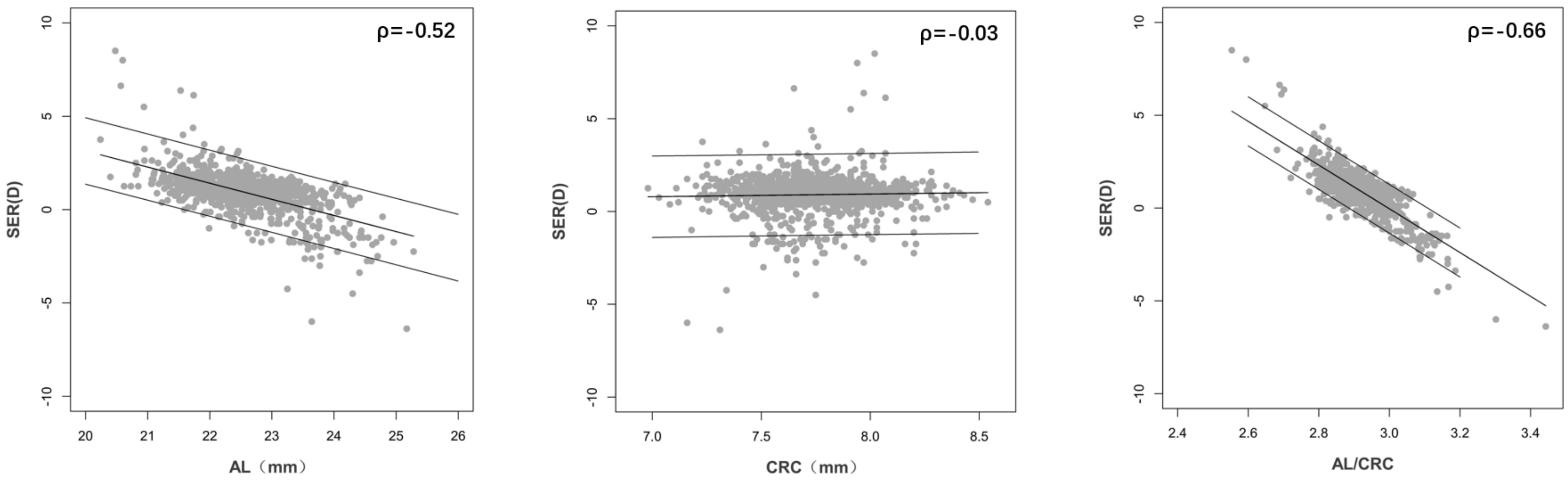

Figure 1. Correlation analysis between AL, CRC, AL/CRC ratio and SER. SER, spherical equivalent refractive error; AL, axial length; CRC, corneal radius of curvature; AL/CRC ratio, axial length to corneal radius of curvature ratio, D, diopter.

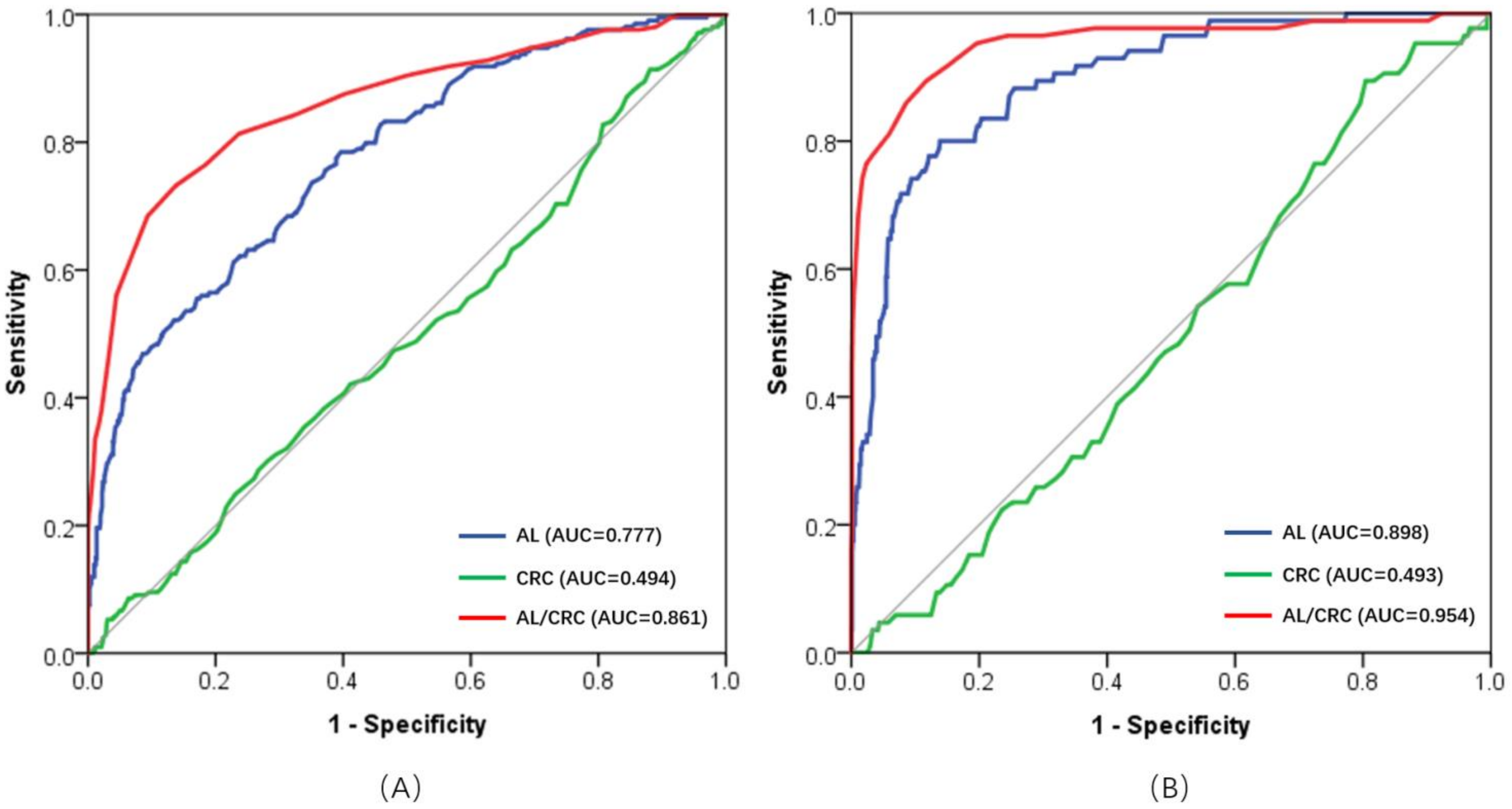

Figure 2. Comparison of the effectiveness of AL, CRC and the AL/CR ratio for hyperopia reserve assessment (A) and myopia assessment (B). AL, axial length; CRC, corneal radius of curvature; AL/CRC ratio, axial length to corneal radius of curvature ratio; AUC, area under curve.

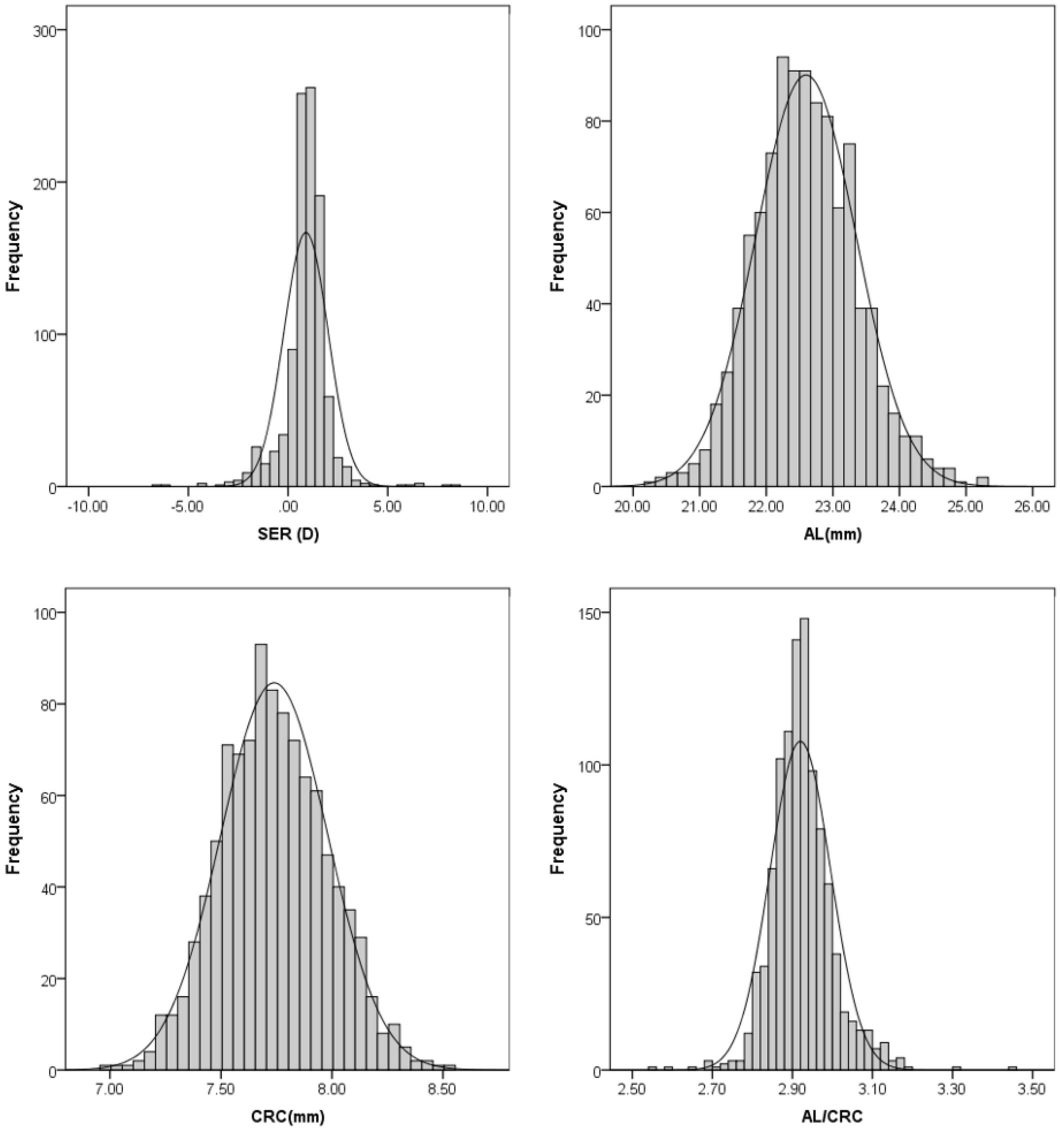

Supplemental Figure 1. Histograms showing distributions of SER, AL, CRC and AL/CRC ratio. SER, spherical equivalent refractive error; AL, axial length; CRC, corneal radius of curvature; AL/CRC ratio, axial length to corneal radius of curvature ratio; D, diopter.

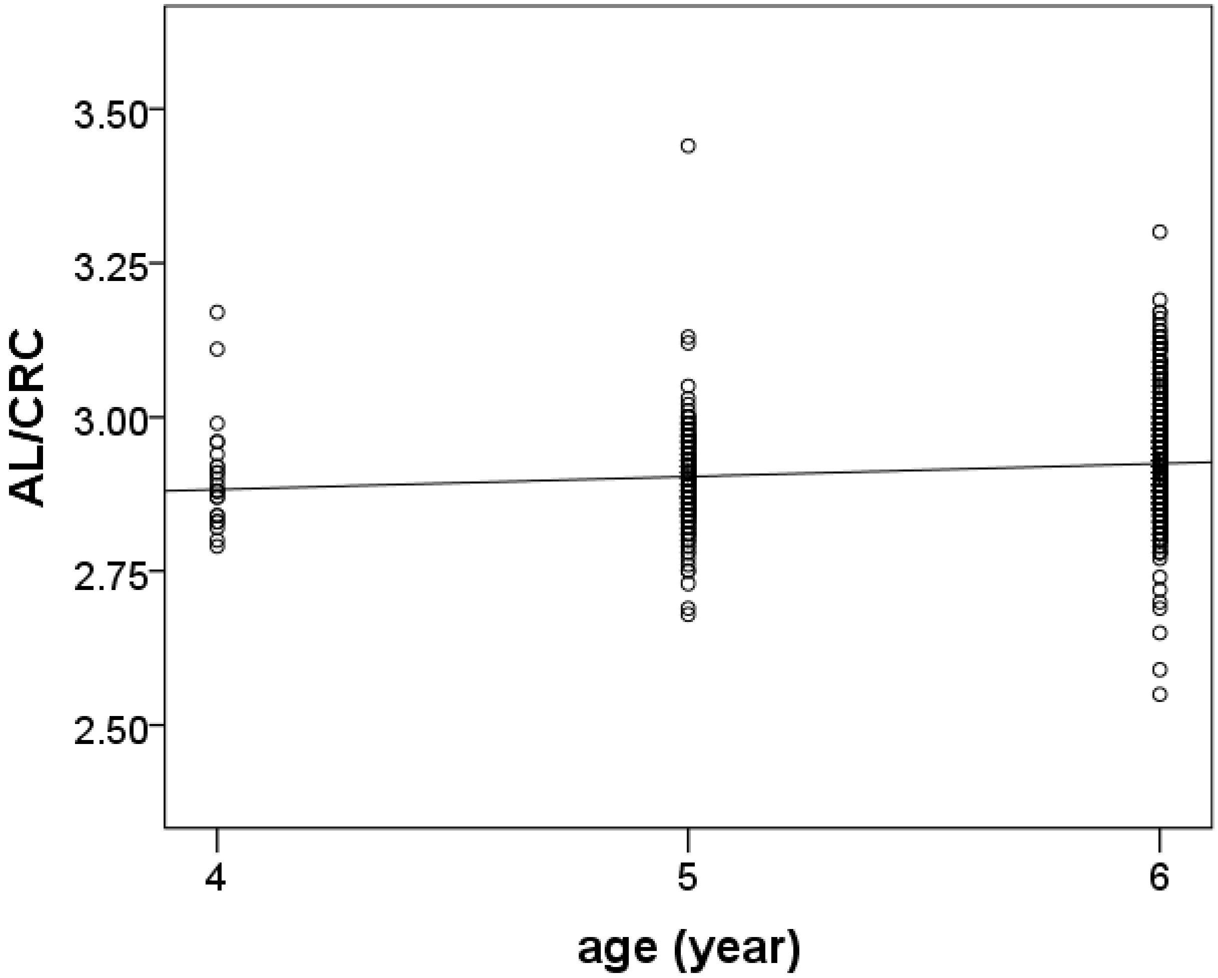

Supplemental Figure 2. Scatterplot of AL/CRC ratio and age. AL/CRC, axial length to corneal radius of curvature ratio.

Supplemental Table 1. Demographic and biometric data of included participants

Parameters	Total, N=1024		Boys, N=537	Girls, N=487	P value
	Mean±SD	Range	Mean±SD	Mean±SD	
Age (years)	5.77±0.47	4-6	5.85±0.35	5.68±0.56	<0.001
SER (D)	0.90±1.11	-6.38 to 8.50	0.90±1.09	0.90±1.13	0.922
AL (mm)	22.59±0.76	20.24 to 25.28	22.62±0.77	22.57±0.74	0.350
CRC (mm)	7.74±0.24	6.98 to 8.54	7.74±0.24	7.74±0.24	0.805
AL/CRC	2.92±0.08	2.65 to 3.17	2.92±0.08	2.92±0.08	0.141
LP (D)	24.09±1.35	19.68 to 28.68	24.02±1.34	24.16±1.35	0.117
ACD (mm)	3.37±0.27	2.09 to 5.37	3.38±0.27	3.34±0.27	0.028

SER, spherical equivalent refractive error; AL, axial length; CRC, corneal radius of curvature; AL/CRC, axial length to corneal radius of curvature ratio; LP, lens power; ACD, anterior chamber depth; D, diopter.

For peer review only

Supplemental Table 2. Association between AL/CRC ratio, AL, and CRC with SER.

Variables	Correlation coefficient	P value	Univariate OR (95% CI)	P value	Gender and age-adjusted Multivariate OR (95% CI)				
					Unstandardised coefficient	95% CI	Standardised coefficient	P value	R^2
AL	-0.52	<0.001	-0.86 (-0.94 to -0.79)	<0.001	-0.869	-0.942 to -0.795	-0.590	<0.001	0.345
CRC	-0.03	0.33	0.15 (-0.14 to 0.43)	0.31	0.148	-0.135 to 0.430	0.032	0.306	0.002
AL/CRC	-0.66	<0.001	-11.73 (-12.27 to -11.19)	<0.001	-11.876	-12.418 to -11.334	-0.809	<0.001	0.645

SER, spherical equivalent refractive error; AL, axial length; CRC, corneal radius of curvature; AL/CRC ratio, axial length to corneal radius of curvature ratio. OR, odds ratio.

For peer review only

Supplemental Table 3. Effect of optimal cut-off value of AL/CRC ratio for the lack of hyperopia reserve assessment and true myopia assessment.

	Criterion	Total Data	True assessment	Error assessment (%)
Lack of hyperopia reserve assessment	AL/CRC ratio ≥2.955	252	196	56 (22.22%)
True myopia assessment	AL/CRC ratio ≥2.975	85	79	6 (7.06%)

AL/CRC ratio, axial length to corneal radius of curvature ratio. Error assessment (%), using optimal cut-off value of AL/CRC ratio to assess the probability of errors in lack of hyperopia reserve or true myopia.

For peer review only

Supplemental Table 4. Summary of studies reporting the AL, CRC, and AL/CRC ratio.

Origin	N	Age (years)	SER (D)	AL/CRC	AL	CRC
Australia [15]	1765	5.5-8.4	+1.26	2.906	22.61	7.70
Iran [17]	4820	40-64	-	3.033	23.14	7.63
China [19]	4411	7-10	-0.13	2.97	23.27	7.82
Singapore [28]	349	3	+0.88	2.81	21.73	7.72
Nigerian [34]	70	20-39	-0.24	3.03	23.74	7.84
Myanmar [35]	1498	40-70	-1.22	2.99	22.76	7.62
Japan [43]	457	4-6	+0.13	2.88	22.35	7.76
Current study	1024	4-6	+0.90	2.92	22.59	7.74

SER, spherical equivalent refractive error; AL, axial length; CRC, corneal radius of curvature; AL/CRC ratio, axial length to corneal radius of curvature ratio.

For peer review only

STROBE Statement—Checklist of items that should be included in reports of ***cross-sectional studies***

	Item No	Recommendation	Page No
**Title and abstract**	1	(*a*) Indicate the study's design with a commonly used term in the title or the abstract	P1
		(*b*) Provide in the abstract an informative and balanced summary of what was done and what was found	P4-5
**Introduction**			
Background/rationale	2	Explain the scientific background and rationale for the investigation being reported	P5-6
Objectives	3	State specific objectives, including any prespecified hypotheses	P6
**Methods**			
Study design	4	Present key elements of study design early in the paper	P6
Setting	5	Describe the setting, locations, and relevant dates, including periods of recruitment, exposure, follow-up, and data collection	P6-7
Participants	6	(*a*) Give the eligibility criteria, and the sources and methods of selection of participants	P6-7
Variables	7	Clearly define all outcomes, exposures, predictors, potential confounders, and effect modifiers. Give diagnostic criteria, if applicable	P7
Data sources/ measurement	8*	For each variable of interest, give sources of data and details of methods of assessment (measurement). Describe comparability of assessment methods if there is more than one group	*P7*
Bias	9	Describe any efforts to address potential sources of bias	P7
Study size	10	Explain how the study size was arrived at	P7
Quantitative variables	11	Explain how quantitative variables were handled in the analyses. If applicable, describe which groupings were chosen and why	P7
Statistical methods	12	(*a*) Describe all statistical methods, including those used to control for confounding	P8
		(*b*) Describe any methods used to examine subgroups and interactions	
		(*c*) Explain how missing data were addressed	P8
		(*d*) If applicable, describe analytical methods taking account of sampling strategy	
		(*e*) Describe any sensitivity analyses	
**Results**			
Participants	13*	(a) Report numbers of individuals at each stage of study—eg numbers potentially eligible, examined for eligibility, confirmed eligible, included in the study, completing follow-up, and analysed	P8
		(b) Give reasons for non-participation at each stage	
		(c) Consider use of a flow diagram	
Descriptive data	14*	(a) Give characteristics of study participants (eg demographic, clinical, social) and information on exposures and potential confounders	P8
		(b) Indicate number of participants with missing data for each variable of interest	
Outcome data	15*	Report numbers of outcome events or summary measures	P8
Main results	16	(*a*) Give unadjusted estimates and, if applicable, confounder-adjusted estimates and their precision (eg, 95% confidence interval). Make clear which confounders were adjusted for and why they were included	P8-14

For peer review only - http://bmjopen.bmj.com/site/about/guidelines.xhtml

		(*b*) Report category boundaries when continuous variables were categorized	
		(*c*) If relevant, consider translating estimates of relative risk into absolute risk for a meaningful time period	
Other analyses	17	Report other analyses done—eg analyses of subgroups and interactions, and sensitivity analyses	
**Discussion**			
Key results	18	Summarise key results with reference to study objectives	P15-17
Limitations	19	Discuss limitations of the study, taking into account sources of potential bias or imprecision. Discuss both direction and magnitude of any potential bias	P17-18
Interpretation	20	Give a cautious overall interpretation of results considering objectives, limitations, multiplicity of analyses, results from similar studies, and other relevant evidence	P18
Generalisability	21	Discuss the generalisability (external validity) of the study results	P18
**Other information**			
Funding	22	Give the source of funding and the role of the funders for the present study and, if applicable, for the original study on which the present article is based	P19

*Give information separately for exposed and unexposed groups.

Note: An Explanation and Elaboration article discusses each checklist item and gives methodological background and published examples of transparent reporting. The STROBE checklist is best used in conjunction with this article (freely available on the Web sites of PLoS Medicine at http://www.plosmedicine.org/, Annals of Internal Medicine at http://www.annals.org/, and Epidemiology at http://www.epidem.com/). Information on the STROBE Initiative is available at www.strobe-statement.org.

For peer review only

For peer review only - http://bmjopen.bmj.com/site/about/guidelines.xhtml
